# Integrative metagenomic and metabolomic analyses reveal the role of gut microbiota in antibody-mediated renal allograft rejection

**DOI:** 10.1186/s12967-022-03825-6

**Published:** 2022-12-23

**Authors:** Xin Li, Ruoying Li, Bingqing Ji, Lili Zhao, Junpeng Wang, Tianzhong Yan

**Affiliations:** 1 Department of Pathophysiology, School of Basic Medical Sciences, Zhengzhou University, Zhengzhou, 450001 Henan China; 2 Collaborative Innovation Center of Henan Province for Cancer Chemoprevention, Zhengzhou University, Zhengzhou, 450001 Henan China; 3 State Key Laboratory of Esophageal Cancer Prevention and Treatment, Zhengzhou University, Zhengzhou, 450052 Henan China; 4 Department of Urology, Henan Provincial People’s Hospital, Zhengzhou University People’s Hospital, Henan University People’s Hospital, Zhengzhou, 450003 Henan China; 5 Department of Organ Transplantation, Zhujiang Hospital, Southern Medical University, Guangzhou, 510280 Guangdong China

**Keywords:** Antibody-mediated rejection, Gut microbiota, Metagenomics, Fecal metabolites, Kidney transplantation

## Abstract

**Background:**

Antibody-mediated rejection (AMR) remains one of the major barriers for graft survival after kidney transplantation. Our previous study suggested a gut microbiota dysbiosis in kidney transplantation recipients with AMR. However, alternations in gut microbial function and structure at species level have not been identified. In the present study, we investigated the metagenomic and metabolic patterns of gut microbiota in AMR patients to provide a comprehensive and in-depth understanding of gut microbiota dysbiosis in AMR.

**Methods:**

We enrolled 60 kidney transplantation recipients, 28 showed AMR and 32 were non-AMR controls with stable post-transplant renal functions. Shotgun sequencing and untargeted LC/MS metabolomic profiling of fecal samples were performed in kidney transplantation recipients with AMR and controls.

**Results:**

Totally, we identified 311 down-regulated and 27 up-regulated gut microbial species associated with AMR after kidney transplantation, resulting in the altered expression levels of 437 genes enriched in 22 pathways, of which 13 were related to metabolism. Moreover, 32 differential fecal metabolites were found in recipients with AMR. Among them, alterations in 3b-hydroxy-5-cholenoic acid, l-pipecolic acid, taurocholate, and 6k-PGF1alpha-d4 directly correlated with changes in gut microbial species and functions. Specific differential fecal species and metabolites were strongly associated with clinical indexes (Cr, BUN, etc.), and could distinguish the recipients with AMR from controls as potential biomarkers.

**Conclusions:**

Altogether, our findings provided a comprehensive and in-depth understanding of the correlation between AMR and gut microbiota, which is important for the etiological and diagnostic study of AMR after kidney transplantation.

**Supplementary Information:**

The online version contains supplementary material available at 10.1186/s12967-022-03825-6.

## Background

Kidney transplantation is considered to be the preferred therapeutic option for patients with end-stage renal disease, as it can significantly prolong the lifespan and improve the quality of life in patients [1]. However, long-term graft survival still remains unsatisfactory. Antibody mediated rejection (AMR) is the major contributor to post-transplant rejection risk and allograft loss, accounting for 30–50% of acute rejection episodes in kidney transplantation [2–4]. AMR is mainly associated with donor-specific antibodies (DSAs) targeting mismatched HLA molecules. The DSAs can destroy allografts through complement-dependent cytotoxicity (CDC) and antibody-dependent cellular cytotoxicity (ADCC) pathways [5, 6]. Current therapies with conventional immunosuppression frequently fail to control DSA production and AMR [7, 8]. Thus, it’s necessary to understand the underlying mechanism and develop novel therapeutic strategies for its efficient treatment.

Increasing evidence showed a relationship between gut microbiota and solid organ allograft rejection. Gut microbiota is thought to be a microbial marker or therapeutic target for the predication and intervention of allograft rejection. Alterations in gut microbiota could impact the host immune system, and are closely associated with acute and chronic allograft rejection in small bowel transplantation (SBT) [9]. In the skin-grafted mice model, differences in the resident microbiome in healthy donors have been suggested to translate into distinct kinetics of graft rejection [10]. Additionally, gut microbiota has been reported to impact chronic murine lung allograft rejection [11]. Our previous study has revealed significant differences in the gut microbial composition between recipients with AMR and the controls with stable renal functions, using 16S rRNA gene sequencing [12]. Specific taxa such as *Clostridiales* could be potentially used as biomarkers to distinguish the recipients with AMR from the controls [12]. However, due to the limitations of 16S rRNA gene sequencing, alternations in gut microbial function and structure at species level have not been identified.

In order to provide direct evidence and comprehensive understanding of gut microbiota dysbiosis associated with antibody-mediated renal allograft rejection, we performed integrative metagenomic and metabolomic analyses of fecal samples in recipients with AMR after kidney transplantation. Overall, we identified 311 down-regulated and 27 up-regulated gut microbial species associated with AMR after kidney transplantation, resulting in the altered expression levels of 437 genes enriched in 22 pathways, of which 13 were related to metabolism. Furthermore, 32 differential fecal metabolites were detected in recipients with AMR. Alterations in fecal metabolites such as 3b-hydroxy-5-cholenoic acid and l-pipecolic acid, directly correlated with changes in gut microbial composition and function. Specific differential fecal species and metabolites could distinguish the recipients with AMR from controls as potential biomarkers.

## Methods

### Study cohort and sample collection.

Totally, 60 kidney transplantation recipients from Henan Provincial People’s Hospital affiliated to Zhengzhou University were enrolled in this study, 28 of which showed AMR (AMR group) and 32 of which were with stable post-transplant renal functions (control group). This study was performed according to ethical guidelines of Henan Provincial People’s Hospital affiliated to Zhengzhou University. AMR was diagnosed with the Banff 2019 criteria [13]. Recipients were excluded if there was a recent history of infection, non-infectious diarrhea, antibiotic usage, or gastric/colon resection. Patients were asked to provide the fecal samples within 24 h after AMR diagnosis. Fecal samples from kidney transplantation recipients with stable renal functions were collected as controls. Fresh stool samples collected from each subject were immediately frozen at − 80 °C until they were processed.

### Shotgun metagenome sequencing

About 100 mg of fecal content were used for DNA extraction using the DNeasy PowerSoil Kit (QIAGEN, Netherlands) following manufacturer’s instructions. The quantity and quality of extracted DNA were checked with a NanoDrop ND-1000 spectrophotometer (Thermo Fisher Scientific, Waltham, MA, USA). Metagenome shotgun libraries with insert sizes of 400 bp were constructed for Illumina sequencers using a TruSeq Nano DNA LT Library Preparation Kit (Illumina) based on manufacturer’s protocols. Sequencing of 2 × 150-bp paired-end reads was performed on an Illumina HiSeq X-ten platform (Illumina, USA) at Personal Biotechnology Co., Ltd. (Shanghai, China).

### Metagenomic data processing and analysis

To obtain high-quality reads for further analysis, raw reads were firstly processed using Cutadapt (v1.2.1) to trim sequencing adapters and low-quality bases, and then mapped to the host genome using BWA (http://bio-bwa.sourceforge.net/) to remove host contamination [14]. The quality-filtered reads were de novo assembled to construct the metagenome for each sample by MEGAHIT (https://hku-bal.github.io/megabox/) [15]. The metagenomic scaffolds longer than 200 bp were used for ORF prediction by MetaGeneMark (http://exon.gatech.edu/GeneMark/metagenome) [16]. All ORFs were clustered by CD-HIT to construct a non-redundant gene catalog (identity > 90%) [17].

Gene abundance in each sample was estimated by soap.coverage (http://soap.genomics.org.cn/) based on the number of aligned reads. For taxa analysis, genes were searched with the lowest common ancestor (LCA) approach against NCBI-NT database by BLASTN (e value < 0.001). The abundance of a taxonomic group was calculated by summing its matching genes. For functional annotation, gene catalogs were annotated using DIAMOND against KEGG databases [18]. Antibiotic resistance and virulence genes of microbiota were identified using Antibiotic Resistance Database (CARD) and Virulence Factor Database (VFDB), respectively [19, 20].

### Metabolomic analyses based on LC/MS

About 100 mg of fecal sample was used for metabolite extraction. Subsequently, 1 mL of ice-cold chloroform/methanol/water (1:2:1, v/v/v) was added to each fecal sample. The homogenates were then incubated at 4 °C for 2 h and centrifuged at 13,000 rpm at 4 °C for 15 min. The supernatant was further filtered using a 0.22 μm membrane filter, blown dry with nitrogen and stored at − 80 °C until use. Meanwhile, an equal aliquot from each sample was mixed to prepare the quality control (QC) samples. A solution of isopropanol/acetonitrile/water (1:1:2, v/v/v) was used to reconstitute samples before LC/MS analysis.

LC/MS Chromatographic separation was performed on an ultra-high-performance liquid chromatography (UHPLC) DIONEX UltiMate_3000 system (Thermo Fisher Scientific, San Jose, CA, USA) equipped with a C18 column, 1.7 µm, 2.1 × 100 mm (Waters Corp., Milford, MA, USA). The flow rate was 0.35 mL/min, the injection volume was 3 μL, and the column temperature was 45 °C. Mobile Phase A consisted of water with 0.1% formic acid (FA), and Mobile Phase B consisted of acetonitrile with 0.1% FA. The gradient elution used was started from 98% A for 0.5 min, linearly decreased to 2% A for 14.5 min, held for 3 min, and finally linearly increased to 98% A to re-equilibrate for 3 min. The QC samples were inserted into the analytical queue to monitor and evaluate the system stability and data reliability. Samples were analyzed by liquid chromatography-tandem mass spectrometry (LC/MS) using UHPLC coupled to a QExactive mass spectrometer (Thermo Fisher, Bremen, Germany). Electrospray ionization (ESI) was performed in positive and negative ion modes. The conditions of the ESI source were as follows: spray voltage, 3.5 kV (ESI+) or 3.2 kV (ESI−); source temperature, 320 °C; sheath gas flow rate, 45 Arb; aux gas flow rate, 15 Arb; mass range, 80–1200 m/z; full ms resolution, 70,000; MS/MS resolution, 17,500; TopN, 10; stepped NCE, (20,40,60); duty cycle, ~ 1.2 s.

Peak alignment, retention time correction, and peak area extraction were performed using the Compound Discoverer 3.0 program [21]. Accurate mass number matching (< 25 ppm) and second-level spectrogram matching were used to retrieve the MZcloud database [11]. Orthogonal partial least-squares-discriminant analysis (OPLS-DA) analysis was performed using the Pareto scaling method and SIMCA-P software [22]. Metabolites with both multidimensional statistical analysis VIP > 1 and univariate statistical analysis *P* value < 0.05 were selected as metabolites with significant differences. The sample preparation and subsequent metabolomic analysis were conducted at Shanghai Personal Biotechnology Co., Ltd. (Shanghai, China).

### Statistical analysis

Wilcoxon rank sum test and Student’s t-test were used for non-normally distributed and normally distributed quantitative data, respectively. Qualitative data were analyzed by chi-square test. Statistical analyses of demographic and clinical characteristics data were conducted with SPSS Statistics (version 22.0.0, IBM SPSS Statistics, IBM Corp., Armonk, NY, USA). Throughout, *P* < 0.05 was regarded as statistically significant.

Alpha-diversity indices (ACE, Chao1, Shannon, and Simpson) were calculated with QIIME (Version 1.9.0). The statistical significance of alpha diversity between groups was evaluated by Mann Whitney U test or Student’s t-test using SPSS. Beta-diversity was calculated by nonmetric multidimensional scaling (NMDS) and hierarchical clustering with the QIIME. Differential abundance of taxa, KO and metabolites was tested by Wilcoxon rank sum test. Only species or KOs with an average relative abundance above 10^−7^ were considered in the analyses. Linear discriminant analysis effect size (*LEfSe*) was also utilized to compare and visualize significant differences in species between groups [23]. Receiver operating characteristic (ROC) analysis was performed to evaluate the diagnostic value.

## Results

### Demographic and clinical characteristics of the kidney transplantation recipients

A total of 60 kidney transplantation recipients, including 28 individuals with AMR and 32 non-rejection controls. Demographic information and clinical characteristics of the recipients were provided in Table 1. The histopathological characteristics of renal biopsy samples for the AMR cases scored according to the Banff 2019 criteria were shown in Additional file 1: Table S1. No differences in age, gender or BMI were detected between the two groups (*P* > 0.05; Table 1). Significantly higher levels of serum creatinine (Cr, *P* < 0.0001), blood urea nitrogen (BUN, *P* < 0.0001), uric acid (UA, *P* = 0.0198), serum cystatin C (CysC, *P* < 0.0001), serum C-reaction protein (CRP, *P* = 0.0005), and urine protein (*P* < 0.0001), and lower levels of serum carbon dioxide (CO_2_, *P* = 0.0019), serum albumin (ALB, *P* < 0.0001), total bile acid (TBA, *P* = 0.0257), hemoglobin (HGB, *P* < 0.0001), and white blood cell (WBC, *P* = 0.0091) were observed in recipients with AMR.

**Table 1:** Comparisons of demographic and clinical characteristics between recipients with AMR and controls

Variable	AMR (n = 28)	Ctrl (n = 32)	*P* value
Age, years	33.29 ± 7.154	37.31 ± 8.525	NS
Male gender, n (%)	25 (89%)	26 (81%)	NS
BMI	21.8 (19.7, 25.53)	23.3 (21.1, 24.95)	NS
Cr, μmol/L	285 (218.3, 417.3)	93.5 (75.25, 100.8)	< 0.0001
BUN, mmol/L	16.6 (12.35, 19.4)	6.625 (5.628, 7.413)	< 0.0001
UA, μmol/L	398.5 (316.3, 458.8)	337.5 (301, 370)	0.0198
CO_2_, mmol/L	21 (18.25, 23)	23.4 (22.05, 25)	0.0019
CysC, mg/L	3.49 (2.78, 4.25)	1.085 (0.975, 1.36)	< 0.0001
ALB, g/L	37.8 (36.15, 39.63)	39.63 (41.45, 48.28)	< 0.0001
TBA, μmol/L	2.75 (2.125, 5.975)	4.65 (3.4, 6.85)	0.0257
LDH, U/L	248.5 (186.8, 327.5)	221 (173.8, 277.5)	NS
ALP, U/L	96 (59.25, 134.3)	77 (70.25, 101)	NS
HGB, g/L	95 (86, 105.5)	139.5 (126.3, 152.5)	< 0.0001
WBC, × 10^9^/L	4.28 (3.523, 6.5)	5.55 (5.01, 6.793)	0.0091
PLT, × 10^9^/L	172 (116.3, 232)	193 (140, 225.8)	NS
CRP, mg/L	1.9 (1.15, 9.41)	0.79 (0.5, 1.755)	0.0005
U-protein, −/±/+/++/+++	0/0/5/9/14	24/4/3/1/0	< 0.0001
Induction therapy, Thymoglobuline, n (%)	28 (100%)	32 (100%)	NS
Maintenance Therapy			
Tacrolimus, n (%)	28 (100%)	32 (100%)	NS
Mycophenolate mofetil, n (%)	28 (100%)	32 (100%)	NS
Prednisone, n (%)	28 (100%)	32 (100%)	NS

Data for age were expressed as mean ± standard deviation and other continuous variables were expressed as median (interquartile range), while categorical variables were reported as counts. Ctrl: control; AMR: antibody-mediated rejection; BMI: body mass index; Cr: creatinine, BUN: blood urea nitrogen; UA: uric acid; CO_2_: carbon dioxide; CysC: serum cystatin C; ALB: albumin; TBA: total bile acid; LDH: lactate dehydrogenase; ALP: alkaline phosphatase; HGB: hemoglobin; WBC: white blood cell; PLT: platelet; CRP: C-reaction protein; U-protein: urine protein; NS: not significant (*P* > 0.05)

### Compositional alteration of gut microbiota in AMR after kidney transplantation

**Fig. 1:**
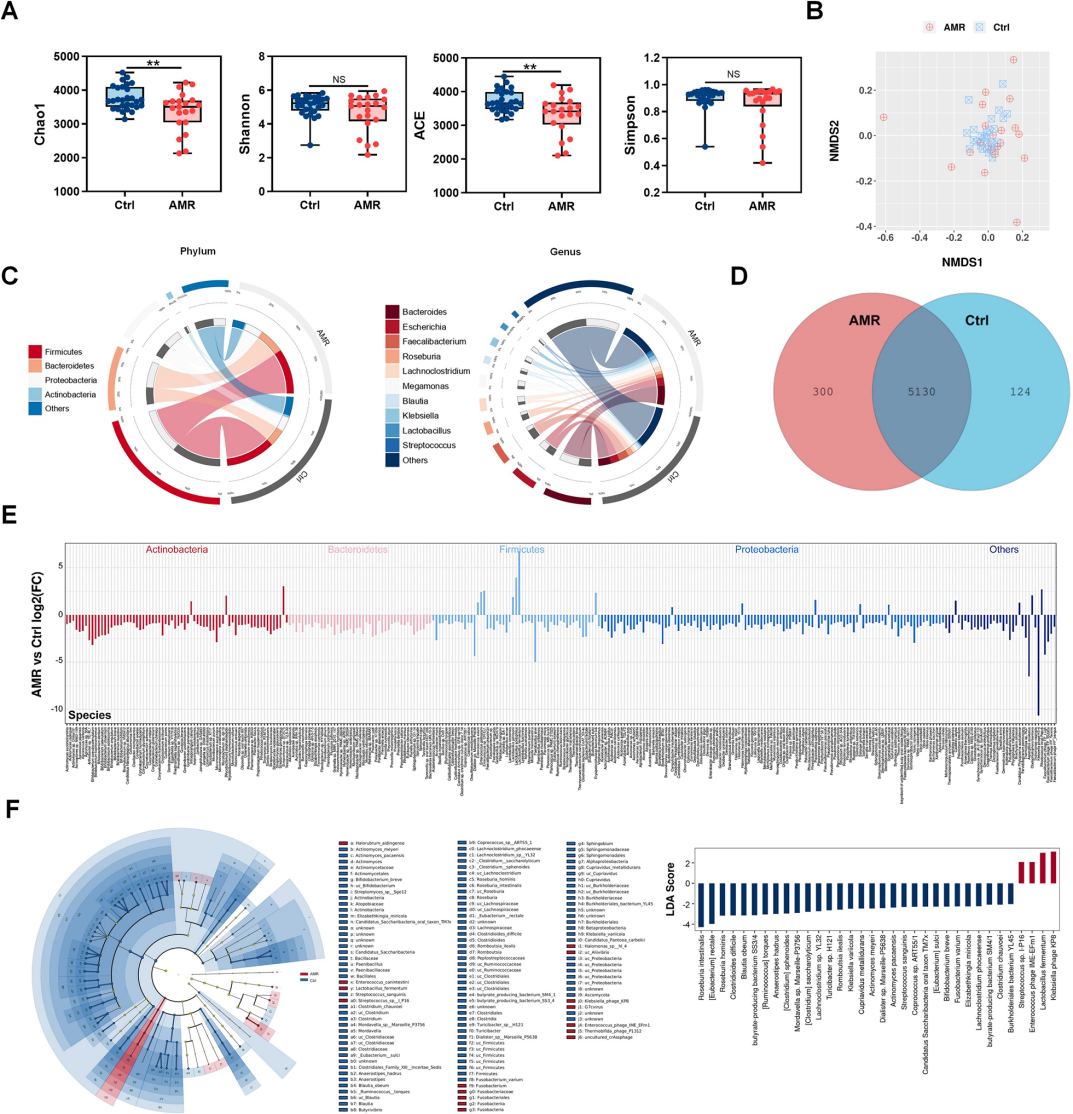
Compositional alteration of gut microbiota in AMR after kidney transplantation. Gut microbiota in AMR and control groups were analyzed with metagenomics. **A** Chao 1, Shannon, ACE, Simpson indexes of alpha diversity. **B** Beta-diversity calculated by nonmetric multidimensional scaling (NMDS) using Bray–Curtis distances for gut microbiota in each group. **C** Contribution of dominant phyla and genera in each group. **D** Venn diagram illustrating the species in gut microbiota in each group. **E** Histogram illustrating the differential species significant changed in AMR group compared to control group (fold change ≥ 1.5 or ≤ 0.67; *P* < 0.05). **F** LDA effect size (LEfSe) analysis of gut microbial species in AMR group compared to control group (LDA > 2; *P* < 0.05). Left panel: a cladogram representation of the taxonomic groups in fecal specimens associated with AMR and controls; right panel: association of specific species with AMR and control groups. Ctrl, control; AMR, antibody-mediated rejection

To comprehensively explore gut microbiota linked to AMR after kidney transplantation, metagenomic sequencing of fecal samples from the AMR and control groups were performed on the Illumina HiSeq X-ten platform at an average depth of about 83 M reads (13 G bp) per sample. We first examined the differences in gut microbial alpha diversity between the AMR and control groups. A significant decrease in species richness (Chao1: *P* = 0.0055; ACE: *P* = 0.0038) was detected in the AMR group, while no differences in microbiota community diversity (Simpson: *P* > 0.05; Shannon: *P* > 0.05) were observed between the two groups (Fig. 1A). For beta diversity, NMDS using Bray–Curtis distances revealed a different distribution of gut microbiota between the AMR and control groups (*P* = 0.002, ANOSIM, Fig. 1B). The relative proportion of dominant taxa at the phylum and genera levels, and their contribution to each group were shown in Fig. 1C. To dissect the detailed taxonomic features involved in AMR, we analyzed metagenomics data at species level. A total of 5554 species were annotated, including 300 species unique to the AMR group, 124 unique to the control group, and 5130 shared by both groups (Fig. 1D). Comparisons between groups were performed using nonparametric analysis. There were 292 and 19 species showing significant decrease and elevation (fold change > 1.5 and *P* < 0.05), respectively (Fig. 1E and Additional file 1: Table S2). The differential species were mainly from *Proteobacteria, Actinobacteria, Firmicutes, and Bacteroidetes* phyla, and about 40% of the species elevated in the AMR group were from *Firmicutes* phylum. The differential abundances of gut microbial taxa at the phylum and genus levels were presented in Additional file 1: Tables S3 and S4. *LEfSe* analysis was also performed to identify the specific species associated with AMR. The results showed elevated *Klebsiella phage KP8*, *Lactobacillus fermentum*, *Enterococcus phage IME-EFm1*, and *Streptococcus sp. I-P16*, and 30 decreased species including *Roseburia intestinalis*, *[Eubacterium] rectale* and *Blautia obeum* in the AMR group (Fig. 1F).

### Functional alteration of gut microbiota in AMR after kidney transplantation

**Fig. 2:**
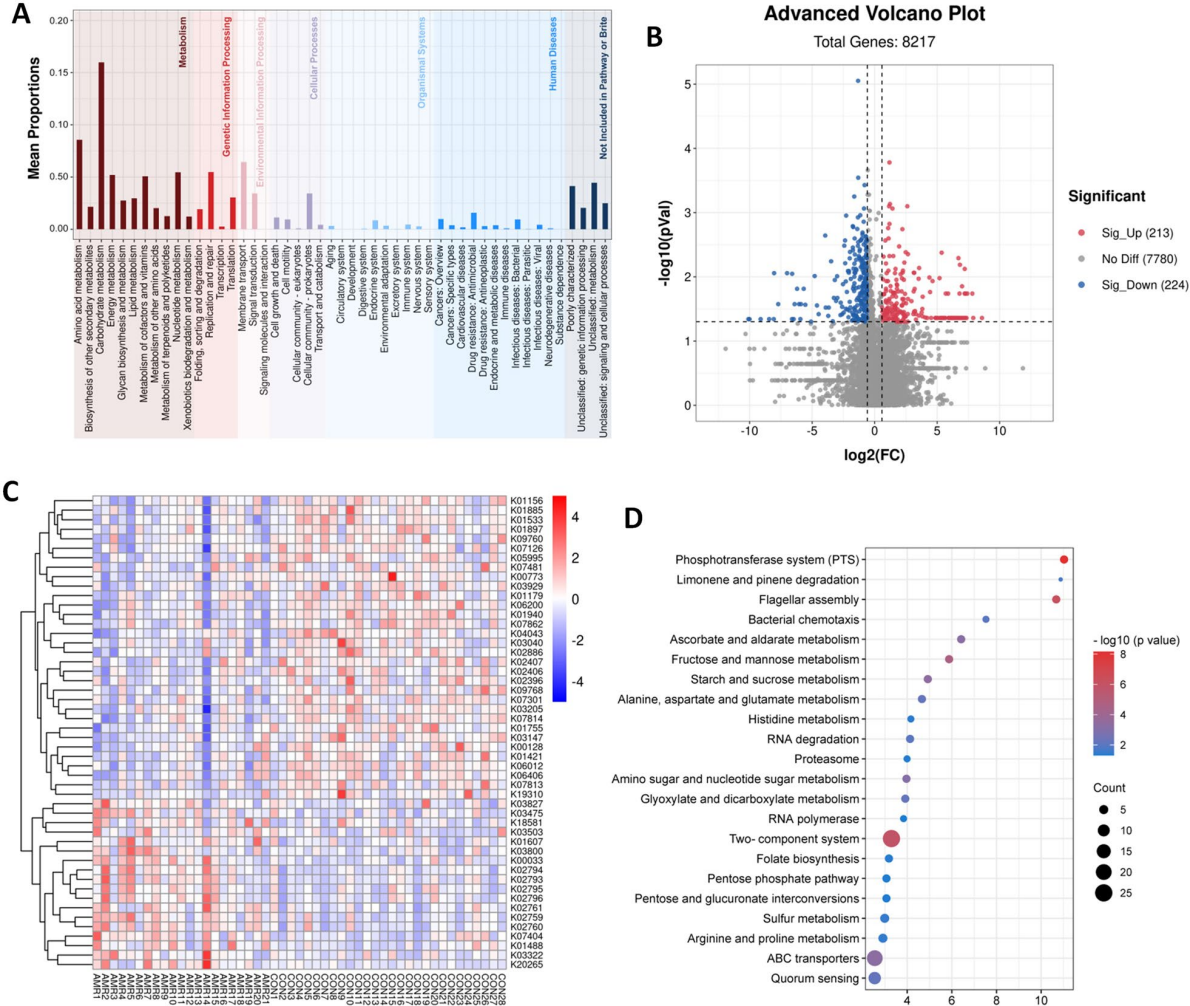
Functional alteration of gut microbiota in AMR after kidney transplantation. Bacterial functional gene in AMR and control groups were analyzed with metagenomics. **A** KEGG annotation and classification of microbial functional genes. **B** Volcano plot for the differential KOs significant changed in AMR group compared to control group (fold change ≥ 1.5 or ≤ 0.67; *P* < 0.05). **C** Heat map of the 50 most differentially expressed KEGG orthologs (KOs). **D** Bubble diagram of KEGG pathways enrichment based on the differential KOs between two groups. Ctrl: control; AMR: antibody-mediated rejection

To investigate the functional properties of gut microbiota between the AMR and control groups, we next performed functional annotations of the metagenome to KEGG modules. Totally, we obtained 8,217 KEGG orthologs (KOs), and most of them were related to metabolism (Fig. 2A). Compared to the control group, 213 KOs were significantly up-regulated, and 224 were significantly down-regulated in the AMR group (Fig. 2B and Additional file 1: Table S5). The first 50 differential KOs of average abundance were exhibited in the heatmap (Fig. 2C). Next, the differentially expressed genes were subjected to KEGG functional enrichment analysis. The bubble chart showed that differential genes were mainly enriched in 22 pathways (for example, Phosphotransferase system, Limonene and pinene degradation, Flagellar assembly, Bacterial chemotaxis, Ascorbate and aldarate metabolism), 13 of which were related to metabolism (Fig. 2D). These functional shifts of microbial metagenome indicated a correlation between AMR and an imbalance of gut microbes involved in the metabolism.

### Fecal metabolomics analyses in AMR after kidney transplantation

**Fig. 3:**
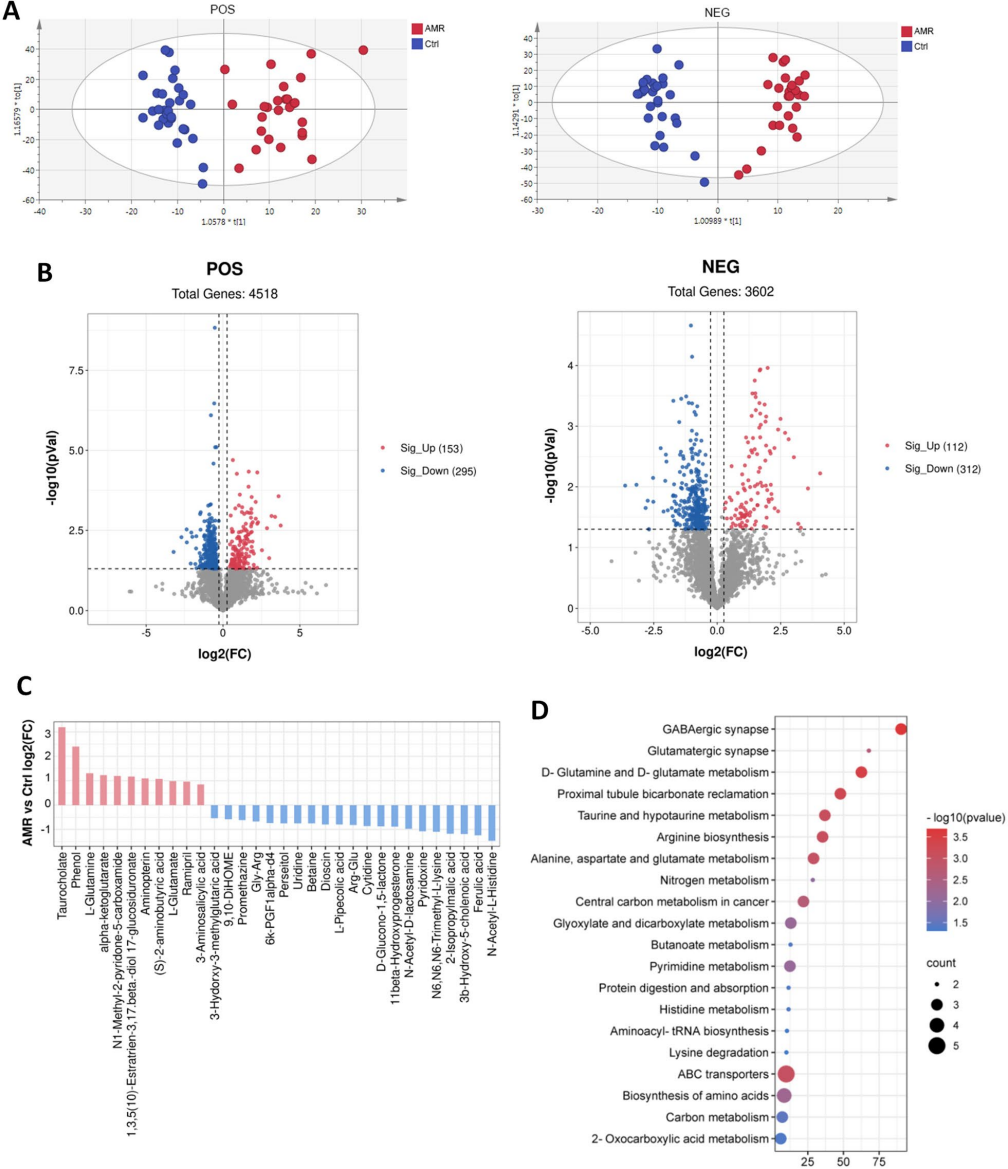
Changes in fecal metabolites in AMR after kidney transplantation. The LC/MS-based untargeted metabolic profiling in positive and negative mode was conducted on fecal samples. **A** OPLS-DA score plots of fecal metabolite profiles derived from recipients with AMR and controls in positive mode (left panel) and negative mode (right panel). **B** Volcano plot for the differential fecal metabolites significant changed in AMR group compared to control group (VIP > 1; *P* < 0.05). **C** Histogram illustrating the differential fecal metabolites significant changed in AMR group compared to control group (VIP > 1; *P* < 0.05). **D** Bubble diagram of metabolic pathways enrichment based on the differential metabolites between two groups. Ctrl, control; AMR, antibody-mediated rejection

Considering that the metabolic functions of gut microbiota between AMR and controls were distinct, metabolic profiling of fecal samples was further performed to assess the impact of a shifted gut microbiome on the metabolic products. The OPLS-DA score map showed a clear separation between AMR and controls using untargeted LC–MS metabolomics in positive and negative mode (Fig. 3A). A total of 8120 m/z features (4518 in POS and 3602 in NEG) were detected, among which 265 (153 in POS and 112 in NEG) were significantly up-regulated, and 607 (295 in POS and 312 in NEG) were significantly down-regulated in the AMR group (Fig. 3B). Of these, 32 differential features were successfully annotated as known metabolites (Fig. 3C). There were 11 metabolites (taurocholate, phenol, l-glutamine, alpha-ketoglutarate, *N*1-methyl-2-pyridone-5-carboxamide, etc.) up-regulated, and 21 metabolites (*N*-acetyl-l-histidine, ferulic acid, 3b-hydroxy-5-cholenoic acid, 2-isopropylmalic acid, N6, N6, N6-trimethyl-l-lysine, etc.) down-regulated in the AMR group (Fig. 3C). The KEGG analysis indicated that the differential metabolites were enriched in 20 pathways including GABAergic synapse, d-Glutamine and d-glutamate metabolism, Proximal tubule bicarbonate reclamation, Taurine and hypotaurine metabolism, and the secondary metabolite biosynthesis and metabolic pathway (Fig. 3D).

### Relationship between AMR-associated fecal microbiota and metabolites

**Fig. 4:**
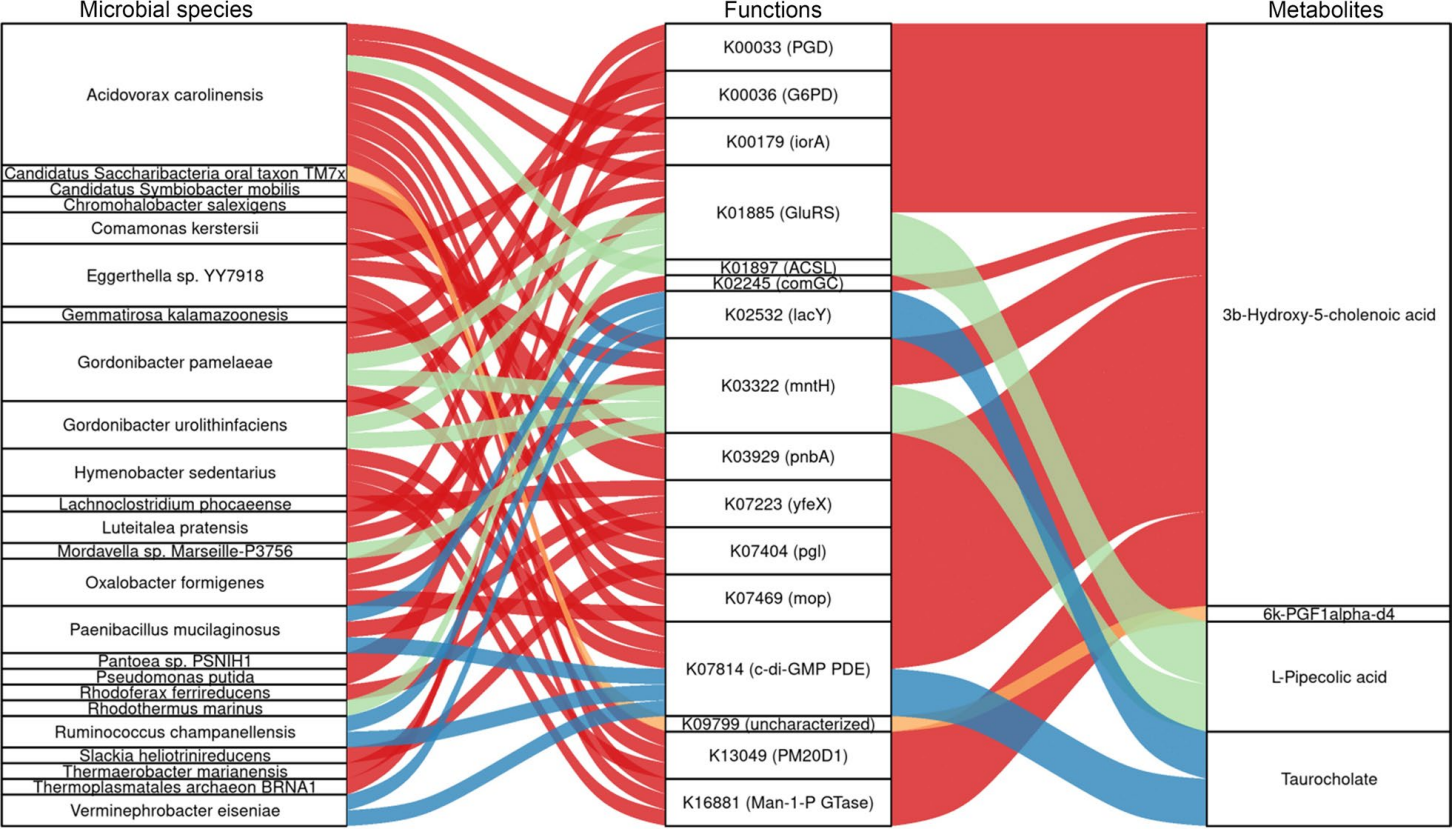
Interrelationship between AMR-associated gut microbial species, functions and metabolites. A Sankey plot was utilized to show the pairwise Spearman correlation between AMR-associated gut microbial species, functions and metabolites (r > 0.5 or < − 0.5; *P* < 0.05). The lines between variables indicated that variables were significantly correlated with each other. Left panel, gut microbial species (24 representative species was shown); middle panel, microbial functional genes; right panel, fecal metabolites; Ctrl: control; AMR: antibody-mediated rejection

Spearman correlation analysis was conducted to further explore the relationships among the AMR-associated gut microbial species, functions and metabolites. A total of 77 microbial species were significantly correlated with 16 functional genes, which were further correlated with 4 metabolites (*P* < 0.05, r > 0.5 or < − 0.5; Fig. 4 and Additional file 1: Table S6). Due to space limitations, the Sankey plot in Fig. 4 presented correlations among 24 representative microbial species, functional genes and metabolites in a schematic manner. Detailed information on the correlation coefficients can be found in Additional file 1: Table S6. Most of the 16 functional genes (6-phosphogluconate dehydrogenase, glucose-6-phosphate 1-dehydrogenase, indolepyruvate ferredoxin oxidoreductase alpha subunit, glutamyl-tRNA synthetase, etc.) correlated with both microbial species and metabolites were metabolic enzyme-related genes (Fig. 4). Notably, all the 77 microbial species demonstrated in Additional file 1: Table S6 were also directly correlated to 4 metabolites including 3b-hydroxy-5-cholenoic acid, l-pipecolic acid, taurocholate, and 6k-PGF1alpha-d4. These data demonstrated the direct interaction between fecal microbiota and metabolites.

### Association between microbial findings and clinical indicators

**Fig. 5:**
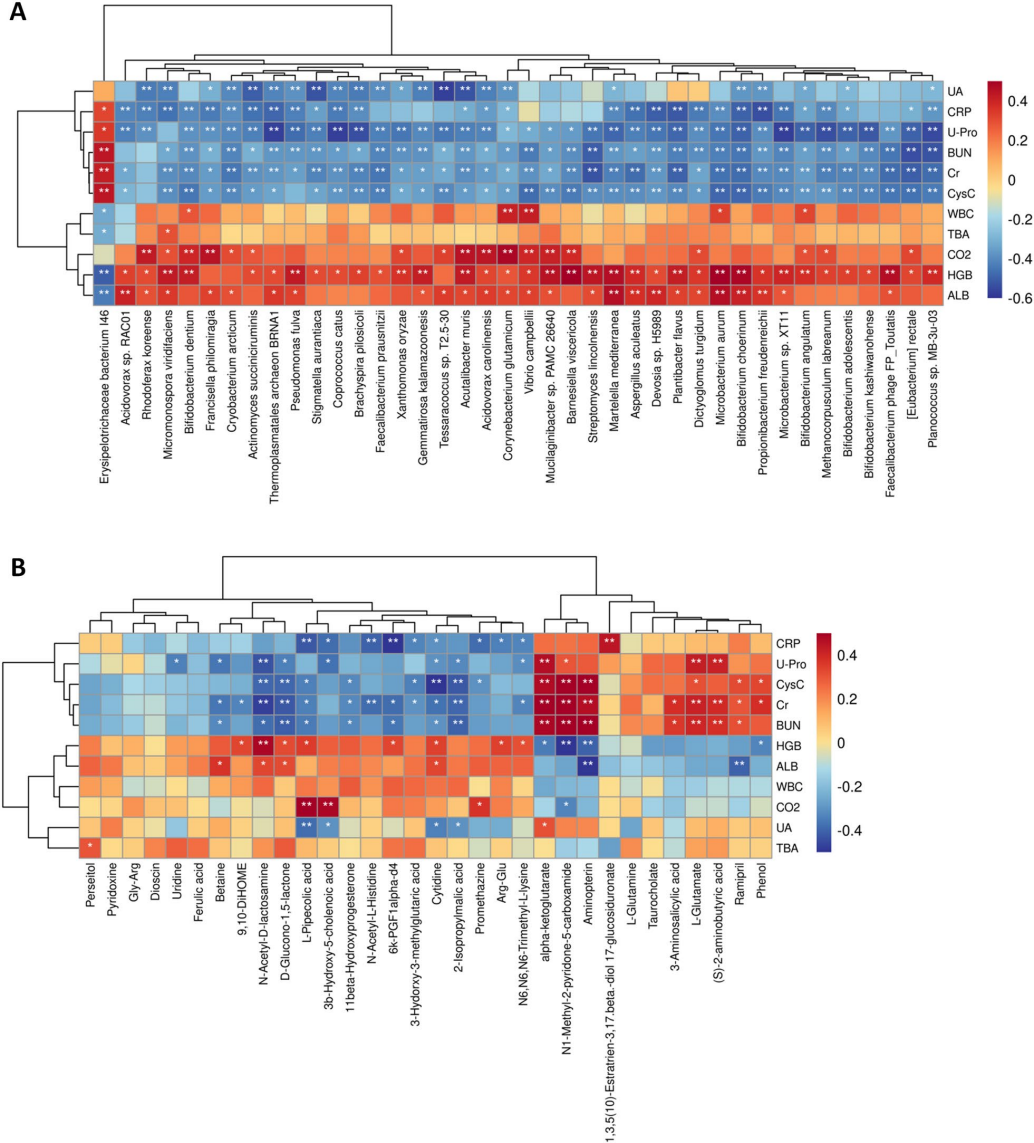
Association between microbial findings and clinical indicators. **A** Spearman correlations between differential gut bacterial species and differential clinical indicators. **B** Spearman correlations between differential fecal metabolites and differential clinical indicators. Positive correlations indicated by red cubes and negative correlations indicated by blue cubes. *P < 0.05; **P < 0.01; Ctrl: control; AMR: antibody-mediated rejection

Based on our results (Table 1), 11 clinical indicators (Cr, BUN, CRP, U-Pro, etc.) were validated to be significantly different between the AMR and control groups. We then evaluated if altered gut microbial species and metabolites in the AMR group were significantly correlated with these clinical indicators using Spearman analysis. A total of 340 microbial species showed significant correlation with one or more clinical indicators (*P* < 0.05, r > 0.3 or < − 0.3; Additional file 1: Table S7). The top 40 species sorted by correlation relevance according to Spearman analysis were shown in Fig. 5A. *Erysipelotrichaceae bacterium I46* was found to be positively correlated with CysC, BUN, Cr, U-Pro and CRP, and negatively correlated with TBA, WBC and ALB (Fig. 5A and Additional file 1: Table S7). Differently, species such as *Corynebacterium glutamicum*, *Martelella mediterranea*, and *Bifidobacterium angulatum* showed positive correlation with CysC, BUN, Cr, U-Pro and CRP, and showed negative correlation with WBC, CO_2_, HGB and ALB (Fig. 5A and Additional file 1: Table S7). Apart from pyridoxine, Gly-Arg, Dioscin, ferulic acid, l-glutamine and taurochola, all the fecal metabolites altered in AMR shared some degree of relatedness with at least one clinical indicators (Fig. 5B and Additional file 1: Table S8). Remarkably, N1-methyl-2-pyridone-5-carboxamide and aminopterin exhibited correlation with multiple clinical indicators (Fig. 5B and Additional file 1: Table S8).

### Evaluation of the diagnostic potential of the microbial and metabolic biomarkers

**Fig. 6:**
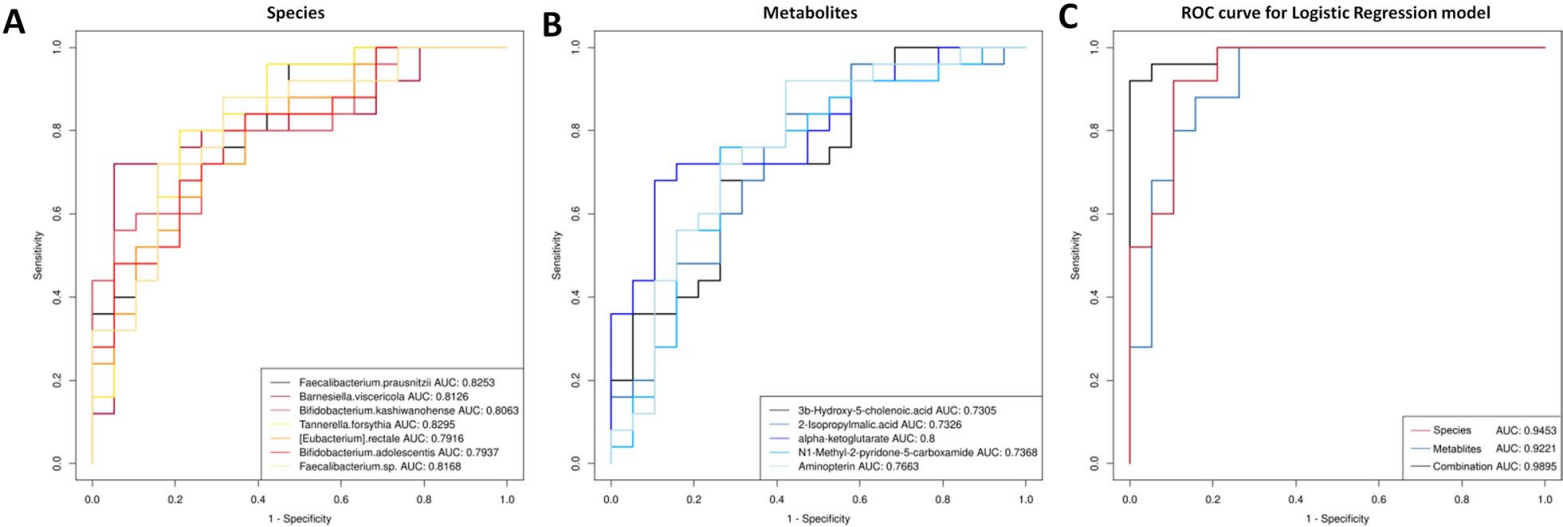
Receiver operating characteristic (ROC) curves of microbial and metabolic biomarkers for discrimination between AMR after kidney transplantation and controls. **A** ROC curves of microbial biomarkers. **B** ROC curves of metabolic biomarkers. **C** ROC curves of the combination of microbial and metabolic biomarkers using logistic regression models. AUC, the total area under the ROC curve; Ctrl: control; AMR: antibody-mediated rejection

To assess the diagnostic potential of the differential gut microbiota and metabolites for discrimination between patients with AMR and controls, ROC curves were constructed. First, we performed AUC estimations based on the abundance data of the top 50 species in the average abundance. Among these species, 20 had AUC values larger than 0.7 (Additional file 1: Table S9), including 7 species with AUC values larger than 0.79, of which the ROC curves were shown in Fig. 6A. Antibiotic resistance and virulence genes of the 20 key species associated with AMR (AUC > 0.7) identified against CARD and VFDB, were shown in Additional file 1: Table S10. The AUC values of the 32 differential metabolites were also calculated, and 15 of them had AUC values larger than 0.7 (Additional file 1: Table S11). ROC curves of the metabolites including alpha-ketoglutarate, N1-methyl-2-pyridone-5-carboxamide, 2-isopropylmalic acid and 3b-hydroxy-5-cholenoic acid were shown in Fig. 6B. For most biomarkers, AUC were < 0.8 (Additional file 1: Tables S9 and S11), indicating that they had moderate diagnostic values. Thus, we analyzed the microbial and metabolic biomarkers with relatively high (Fig. 6A and B) using multivariate logistic regression. Taken together, the 7 species had a combined AUC of 0.9453, and the 4 metabolites had a combined AUC of 0.8526 (Fig. 6C). The combination model with both the microbial and metabolic biomarkers (AUC = 0.9726) outperformed the species or metabolite only model in the discrimination of the patients with AMR from the controls (Fig. 6C). These results indicated that the gut microbiota and metabolites may function as biomarkers to distinguish patients with AMR from the controls.

## Discussion

In this study, we provided extended details regarding the role of gut microbiota in recipients with AMR after kidney transplantation with metagenomics and metabolomics. Totally, we identified 311 down-regulated and 27 up-regulated species associated with AMR. Changes in gut microbiota mainly resulted in the altered metabolic function, for example, Ascorbate and aldarate metabolism, Fructose and mannose metabolism, and Starch and sucrose metabolism Alanine. The fecal metabolome of recipients with AMR was also dramatically changed compared to controls. Correlations were observable between the fecal metabolites and microbiota. Moreover, specific differential fecal species and metabolites were strongly associated with the clinical indexes of AMR, and may serve as diagnostic biomarkers.

The present study demonstrated gut dysbiosis in recipients with AMR after kidney transplantation. A similar phenomenon was also observed in rats with acute rejection after liver transplantation [24]. Early-life disruption of the gut microbiota was reported to cause acute vascular rejection, which was related to exacerbate immune responses [25]. Consistent with our previous findings based on 16s rDNA sequencing [12], the alteration of gut microbiota diversity in recipients with AMR mainly reflected in decreased Chao 1 and ACE indices, while the changes in Shannon and Simpson indices were not apparent. Since metagenomic sequencing is a powerful approach with a high taxonomic accuracy at the species level for studying microbial communities [26], we performed an in-depth characterization of the gut flora in AMR, and identified 311 down-regulated and 27 up-regulated species.

The top five differential species based on relative abundance were *Faecalibacterium prausnitzii, [Eubacterium] rectale, [Ruminococcus] torques, Coprococcus catus,* and *Bifidobacterium pseudocatenulatum*, and all their relative abundance were decreased in recipients with AMR. *Faecalibacterium prausnitzii*, the most important butyrate-producing bacteria in human colon, was previously reported to be negatively correlated with inflammatory bowel disease and colorectal cancer [27]. Generally, *Faecalibacterium prausnitzii* occupied an anti-inflammatory role by producing metabolites (butyrate and salicylic acid) and inducing IL-10 [28, 29]. Similarly, *[Eubacterium] rectale* and *Bifidobacterium pseudocatenulatum* could help to maintain intestinal barrier and suppress inflammation activation through inhibiting CD83 and TLR4/NF-κB, respectively [30, 31]. Increased *Lactobacillus* counts were observed in patients with chronic kidney disease and recipients with AMR [12, 32]. Here, we more accurately identified increased *Lactobacillus fermentum*, *Lactobacillus johnsonii* and *Lactobacillus acidophilus* in recipients with AMR after kidney transplantation using shotgun metagenomic sequencing, all of which were demonstrated to have the effect of enhancing immune response, especially antibody response [33–35]. Possibly, the gut microbiota dysbiosis with decrease in immunosuppressive species and decrease in immune enhancing species in recipients could promote AMR through enhancing the donor specific antibody response.

Changes of gut microbiota usually resulted in functional alteration. In the present work, we totally identified 437 differential KOs between recipients with AMR and the controls, which were enriched in 22 pathways. The differences in metabolic pathways (Arginine and proline metabolism, Sulfur metabolism, Pentose and glucuronate interconversions, etc.) caused by alteration of gut microbiota in AMR were the most obvious, which was similar to the prediction from our previous study by PICRUSt analysis [12]. Thus, we performed metabolomics analysis to further investigate the metabolic changes, and found 11 metabolites (taurocholate, phenol, l-glutamine, alpha-ketoglutarate, N1-methyl-2-pyridone-5-carboxamide, etc.) up-regulated, and 21 metabolites (*N*-acetyl-l-histidine, ferulic acid, 3b-hydroxy-5-cholenoic acid, 2-isopropylmalic acid, N6, N6, N6-trimethyl-l-lysine, etc.) down-regulated in fecal samples from recipients with AMR. A serum metabolomics study of the acute graft rejection in human renal transplantation based on liquid chromatography-mass spectrometry have revealed comprehensive metabolic abnormalities in acute graft rejection [36]. Metabolites such as creatinine, kynurenine, uric acid, polyunsaturated fatty acid, phosphatidylcholines, sphingomyelins, and lysophosphatidylcholines were identified as discriminative metabolites in the serum from the acute graft rejection after transplantation [36].

Among the differential fecal metabolites we identified, 3b-hydroxy-5-cholenoic acid, l-pipecolic acid, taurocholate, and 6k-PGF1alpha-d4 were directly correlated with altered gut microbial species and the related functional genes of enzymes. Both 3b-hydroxy-5-cholenoic acid and taurocholate were metabolites involved in bile acid metabolism [37, 38]. Consistent with our results, taurocholate was also detected with higher intensity in fecal samples from patients with rejection after intestinal transplantation compared to non-rejection ones [38]. Increased concentrations of glycocholate plus glycochenodeoxycholate and taurocholate/taurochenodeoxycholate ratios could be used for early detection of hepatic allograft dysfunction [39]. Moreover, elevated taurocholic acid and glycocholic acid in the bronchoalveolar lavage were reported to be associated with concurrent acute lung allograft dysfunction and inflammatory proteins [40]. Taken together, combining these literature reports and our data, we inferred that the gut microbiota mediated-taurocholate alteration played a crucial role in promoting AMR after kidney transplantation. Few reports on the functions of 3b-hydroxy-5-cholenoic acid, l-pipecolic acid, and 6k-PGF1alpha-d4 have been published, therefore further research is necessary to demonstrate their role in AMR. Based on the above results, we hypothesize that changes of gut microbiota structure and function could result in the alteration of the fecal metabolites, and in turn may impact the pathogenesis and progression of AMR. It is still noteworthy that causal conclusions cannot be drawn from our data, and further Mendelian randomization studies are needed to confirm this hypothesis. This will have important implications for understanding the precise role of gut microbiota in AMR.

Besides the metabolites mentioned above, N1-methyl-2-pyridone-5-carboxamide and aminopterin should also be noted, since they exhibited high correlation with multiple clinical indicators of kidney function. N1-methyl-2-pyridone-5-carboxamide is an end product of NAD^+^ catabolism. Previously, Rutkowski et al. have suggested that high serum concentrations of N1-methyl-2-pyridone-5-carboxamide in chronic renal failure resulted from kidney function injury, since the serum concentrations of N1-methyl-2-pyridone-5-carboxamide were approximately 20-fold higher in patients with advanced renal failure than in healthy controls, which could decline after dialysis or kidney transplantation [41]. Strong associations of urinary N1-methyl-2-pyridone-5-carboxamide/N1-methylnicotinamide with kidney function has also been demonstrated by Azer et al. [42]. Accordingly, elevation of N1-methyl-2-pyridone-5-carboxamide in fecal sample observed in our study could be also associated with renal dysfunction induced by AMR. Aminopterin, as a folic acid antagonist, has been previously used for the treatment of leukemia and rheumatoid arthritis [43, 44]. However, the unsatisfactory therapeutic effects and unpredictable toxicities of aminopterin limit its clinical application [45]. Interestingly, during treatments, all the patients enrolled in this study didn’t have aminopterin which also could not be generated by metabolizing drugs in regimens. From this it was hypothesized that increased aminopterin in fecal samples from recipients with AMR was endogenous. However, the specific mechanism may require further research.

Banff criteria, a combination of serologic (circulating DSA), histologic (primarily microvascular inflammation and transplant glomerulopathy), and immunohistologic (C4d staining in peritubular capillaries) criteria, is the gold standard for the diagnosis of AMR after kidney transplantation [13]. Histologic and immunohistologic evidences could be accessed in invasive manners, thus the identification of novel non-invasive potential biomarkers for the effective diagnosis of AMR is necessary. It has recently been shown that gut microbiota and their metabolites could be used as markers to distinguish patients with colorectal cancer or chronic kidney disease from healthy individuals [46, 47]. In this study, we also identified a series of microbial and metabolomic markers to discriminate kidney transplantation recipients with AMR from cases with stable kidney function. Of note, the combination model with both the microbial and metabolic markers had the AUC more than 0.9, suggesting that it may have high diagnostic value for AMR. Easily accessible fecal samples and improvements in multiomic technologies will enable microbiota-based diagnosis for recipients with AMR.

There are some limitations in the present study that must be recognized. Firstly, our findings warrant further confirmation with an external cohort. Secondly, the data extracted from non-transplant fecal samples was absent. Comparing the findings of this study to the data extracted from non-transplant fecal samples will provide a metagenomic and metabolic background for the allograft recipients, and further studies will be required to address this important issue. Thirdly, we didn’t take account of the compositional nature of microbiome datasets in the selection of the analysis methods. The counts of sequencing reads assigned to organisms were normalized to a constant area. Thus, our results could reflect only changes in the relative abundance of the microbiota but not the absolute abundance.

## Conclusions

In conclusion, our current study highlighted the gut microbiota dysbiosis at the species level in AMR after kidney transplantation, which was associated with an alteration of the gut microbiota functions and a change in fecal metabolites. Moreover, specific species and metabolites were well associated with kidney function indicators, and could be used as biomarkers to distinguish kidney recipients with AMR from the ones with stable kidney functions. Altogether, these findings provided a comprehensive and in-depth understanding of the correlation between AMR and gut microbiota, which is important for the etiological and diagnostic study of AMR after kidney transplantation.

## Supplementary Information

**Additional file 1: Table S1.** Histopathological **characteristics** according to the Banff 2019 criteria of AMR cases. **Table S2.** Comparison of relative abundance of gut microbiota between AMR and control groups at the species level. **Table S3.** Comparison of relative abundance of gut microbiota between AMR and control groups at the phylum level. **Table S4.** Comparison of relative abundance of gut microbiota between AMR and control groups at the genus level. **Table S5.** Comparison of relative abundance of KOs between AMR and control groups. **Table S6.** Interrelationship between AMR-associated gut microbial species, functions and metabolites. **Table S7.** Spearman correlation between species and clinical indicators. **Table S8.** Spearman correlation between metabolites and clinical indicators. **Table S9.** Area under the ROC curves of microbial biomarkers. **Table S10.** Antibiotic resistance and virulence genes of key species associated with AMR after kidney transplantation. **Table S11.** Area under the ROC curves of metabolic biomarkers

## Data Availability

All sequencing data associated with this study were uploaded to the GenBank Sequence Read Archive (https://www.ncbi.nlm.nih.gov/sra; accession number: PRJNA836621).

## References

[1] Tonelli M, Wiebe N, Knoll G, Bello A, Browne S, Jadhav D, Klarenbach S, Gill J (2011). Systematic review: kidney transplantation compared with dialysis in clinically relevant outcomes. Am J Transplant.

[2] Matas AJ, Smith JM, Skeans MA, Thompson B, Gustafson SK, Stewart DE, Cherikh WS, Wainright JL, Boyle G, Snyder JJ (2015). OPTN/SRTR 2013 annual data report: kidney. Am J Transplant.

[3] Loupy A, Lefaucheur C, Vernerey D, Prugger C, Duong van Huyen JP, Mooney N, Suberbielle C, Fremeaux-Bacchi V, Mejean A, Desgrandchamps F (2013). Complement-binding anti-HLA antibodies and kidney-allograft survival. N Engl J Med.

[4] Davis S, Cooper JE (2017). Acute antibody-mediated rejection in kidney transplant recipients. Transplant Rev.

[5] Kim M, Martin ST, Townsend KR, Gabardi S (2014). Antibody-mediated rejection in kidney transplantation: a review of pathophysiology, diagnosis, and treatment options. Pharmacotherapy.

[6] Lucas JG, Co JP, Nwaogwugwu UT, Dosani I, Sureshkumar KK (2011). Antibody-mediated rejection in kidney transplantation: an update. Expert Opin Pharmacother.

[7] Loupy A, Lefaucheur C (2018). Antibody-mediated rejection of solid-organ allografts. N Engl J Med.

[8] Eskandary F, Regele H, Baumann L, Bond G, Kozakowski N, Wahrmann M, Hidalgo LG, Haslacher H, Kaltenecker CC, Aretin MB (2018). A randomized trial of bortezomib in late antibody-mediated kidney transplant rejection. J Am Soc Nephrol.

[9] Wang C, Li Q, Li J (2018). Gut microbiota and its implications in small bowel transplantation. Front Med.

[10] McIntosh CM, Chen L, Shaiber A, Eren AM, Alegre ML (2018). Gut microbes contribute to variation in solid organ transplant outcomes in mice. Microbiome.

[11] Lu D, Xue L, Feng C, Jin Y, Wu C, Xie C, Gonzalez FJ, Wang G, Zhou Z (2019). A systemic workflow for profiling metabolome and lipidome in tissue. J Chromatogr A.

[12] Wang J, Li X, Wu X, Wang Z, Zhang C, Cao G, Liu S, Yan T (2021). Gut microbiota alterations associated with antibody-mediated rejection after kidney transplantation. Appl Microbiol Biotechnol.

[13] Loupy A, Haas M, Roufosse C, Naesens M, Adam B, Afrouzian M, Akalin E, Alachkar N, Bagnasco S, Becker JU (2020). The Banff 2019 Kidney Meeting Report (I): updates on and clarification of criteria for T cell- and antibody-mediated rejection. Am J Transplant.

[14] Li H, Durbin R (2009). Fast and accurate short read alignment with Burrows-Wheeler transform. Bioinformatics.

[15] Li D, Liu CM, Luo R, Sadakane K, Lam TW (2015). MEGAHIT: an ultra-fast single-node solution for large and complex metagenomics assembly via succinct de Bruijn graph. Bioinformatics.

[16] Zhu W, Lomsadze A, Borodovsky M (2010). Ab initio gene identification in metagenomic sequences. Nucleic Acids Res.

[17] Fu L, Niu B, Zhu Z, Wu S, Li W (2012). CD-HIT: accelerated for clustering the next-generation sequencing data. Bioinformatics.

[18] Buchfink B, Xie C, Huson DH (2015). Fast and sensitive protein alignment using DIAMOND. Nat Methods.

[19] Jia B, Raphenya AR, Alcock B, Waglechner N, Guo P, Tsang KK, Lago BA, Dave BM, Pereira S, Sharma AN (2017). CARD 2017: expansion and model-centric curation of the comprehensive antibiotic resistance database. Nucleic Acids Res.

[20] Chen L, Zheng D, Liu B, Yang J, Jin Q (2016). VFDB 2016: hierarchical and refined dataset for big data analysis–10 years on. Nucleic Acids Res.

[21] Huan T, Forsberg EM, Rinehart D, Johnson CH, Ivanisevic J, Benton HP, Fang M, Aisporna A, Hilmers B, Poole FL (2017). Systems biology guided by XCMS Online metabolomics. Nat Methods.

[22] Triba MN, Le Moyec L, Amathieu R, Goossens C, Bouchemal N, Nahon P, Rutledge DN, Savarin P (2015). PLS/OPLS models in metabolomics: the impact of permutation of dataset rows on the K-fold cross-validation quality parameters. Mol Biosyst.

[23] Segata N, Izard J, Waldron L, Gevers D, Miropolsky L, Garrett WS, Huttenhower C (2011). Metagenomic biomarker discovery and explanation. Genome Biol.

[24] Lu H, He J, Wu Z, Xu W, Zhang H, Ye P, Yang J, Zhen S, Li L (2013). Assessment of microbiome variation during the perioperative period in liver transplant patients: a retrospective analysis. Microb Ecol.

[25] Rey K, Manku S, Enns W, Van Rossum T, Bushell K, Morin RD, Brinkman FSL, Choy JC (2018). Disruption of the gut microbiota with antibiotics exacerbates acute vascular rejection. Transplantation.

[26] Verce M, De Vuyst L, Weckx S (2019). Shotgun metagenomics of a water kefir fermentation ecosystem reveals a novel Oenococcus species. Front Microbiol.

[27] Ferreira-Halder CV, Faria AVS, Andrade SS (2017). Action and function of *Faecalibacterium prausnitzii* in health and disease. Best Pract Res Clin Gastroenterol.

[28] Sokol H, Pigneur B, Watterlot L, Lakhdari O, Bermudez-Humaran LG, Gratadoux JJ, Blugeon S, Bridonneau C, Furet JP, Corthier G (2008). *Faecalibacterium prausnitzii* is an anti-inflammatory commensal bacterium identified by gut microbiota analysis of Crohn disease patients. Proc Natl Acad Sci U S A.

[29] Gullon B, Gullon P, Tavaria FK, Yanez R (2016). Assessment of the prebiotic effect of quinoa and amaranth in the human intestinal ecosystem. Food Funct.

[30] Islam SMS, Ryu HM, Sayeed HM, Byun HO, Jung JY, Kim HA, Suh CH, Sohn S (2021). Eubacterium rectale attenuates HSV-1 induced systemic inflammation in mice by inhibiting CD83. Front Immunol.

[31] Chen Y, Yang B, Stanton C, Ross RP, Zhao J, Zhang H, Chen W (2021). Bifidobacterium pseudocatenulatum ameliorates DSS-induced colitis by maintaining intestinal mechanical barrier, blocking proinflammatory cytokines, inhibiting TLR4/NF-kappaB signaling, and altering gut microbiota. J Agric Food Chem.

[32] Lopes R, Balbino KP, Jorge MP, Ribeiro AQ, Martino HSD, Alfenas RCG (2018). Modulation of intestinal microbiota, control of nitrogen products and inflammation by pre/probiotics in chronic kidney disease: a systematic review. Nutr Hosp.

[33] Alaqil AA, Abbas AO, El-Beltagi HS, El-Atty HKA, Mehaisen GMK, Moustafa ES (2020). Dietary supplementation of probiotic *Lactobacillus acidophilus* modulates cholesterol levels, immune response, and productive performance of laying hens. Animals.

[34] Olivares M, Diaz-Ropero MP, Sierra S, Lara-Villoslada F, Fonolla J, Navas M, Rodriguez JM, Xaus J (2007). Oral intake of *Lactobacillus fermentum* CECT5716 enhances the effects of influenza vaccination. Nutrition.

[35] Taheri HR, Moravej H, Tabandeh F, Zaghari M, Shivazad M (2010). Efficacy of combined or single use of *Lactobacillus crispatus* LT116 and *L. johnsonii* LT171 on broiler performance. Br Poult Sci.

[36] Zhao X, Chen J, Ye L, Xu G (2014). Serum metabolomics study of the acute graft rejection in human renal transplantation based on liquid chromatography-mass spectrometry. J Proteome Res.

[37] Setchell KD, Schwarz M, O'Connell NC, Lund EG, Davis DL, Lathe R, Thompson HR, Weslie Tyson R, Sokol RJ, Russell DW (1998). Identification of a new inborn error in bile acid synthesis: mutation of the oxysterol 7alpha-hydroxylase gene causes severe neonatal liver disease. J Clin Invest.

[38] Girlanda R, Cheema AK, Kaur P, Kwon Y, Li A, Guerra J, Matsumoto CS, Zasloff M, Fishbein TM (2012). Metabolomics of human intestinal transplant rejection. Am J Transplant.

[39] Azer SA, McCaughan GW, Stacey NH (1994). Daily determination of individual serum bile acids allows early detection of hepatic allograft dysfunction. Hepatology.

[40] Zhang CYK, Ahmed M, Huszti E, Levy L, Hunter SE, Boonstra KM, Moshkelgosha S, Sage AT, Azad S, Zamel R (2020). Bronchoalveolar bile acid and inflammatory markers to identify high-risk lung transplant recipients with reflux and microaspiration. J Heart Lung Transplant.

[41] Rutkowski B, Slominska E, Szolkiewicz M, Smolenski RT, Striley C, Rutkowski P, Swierczynski J (2003). *N*-methyl-2-pyridone-5-carboxamide: a novel uremic toxin?. Kidney Int Suppl.

[42] Deen CPJ, van der Veen A, Gomes-Neto AW, Geleijnse JM, van den Borgonjen Berg KJ, Heiner-Fokkema MR, Kema IP, Bakker SJL (2020). Urinary excretion of N(1)-methyl-2-pyridone-5-carboxamide and N(1)-methylnicotinamide in renal transplant recipients and donors. J Clin Med.

[43] Gubner R, August S, Ginsberg V (1951). Therapeutic suppression of tissue reactivity. II. Effect of aminopterin in rheumatoid arthritis and psoriasis. Am J Med Sci.

[44] Gubner R (1951). Therapeutic suppression of tissue reactivity. I. Comparison of the effects of cortisone and aminopterin. Am J Med Sci.

[45] Peiro Cadahia J, Bondebjerg J, Hansen CA, Previtali V, Hansen AE, Andresen TL, Clausen MH (2018). Synthesis and evaluation of hydrogen peroxide sensitive prodrugs of methotrexate and aminopterin for the treatment of rheumatoid arthritis. J Med Chem.

[46] Wu IW, Gao SS, Chou HC, Yang HY, Chang LC, Kuo YL, Dinh MCV, Chung WH, Yang CW, Lai HC (2020). Integrative metagenomic and metabolomic analyses reveal severity-specific signatures of gut microbiota in chronic kidney disease. Theranostics.

[47] Yachida S, Mizutani S, Shiroma H, Shiba S, Nakajima T, Sakamoto T, Watanabe H, Masuda K, Nishimoto Y, Kubo M (2019). Metagenomic and metabolomic analyses reveal distinct stage-specific phenotypes of the gut microbiota in colorectal cancer. Nat Med.

