# Supplementary material for: Integrative metagenomic and metabolomic analyses reveal the role of gut microbiota in antibody-mediated renal allograft rejection
Source: J Transl Med. 2022 Dec 23;20:614. doi: 10.1186/s12967-022-03825-6 (PMC9784291; doi:10.1186/s12967-022-03825-6)
Supplement: Supplementary file 1 — Additional file 1: Table S1. Histopathological characteristics according to the Banff 2019 criteria of AMR cases. Table S2. Comparison of relative abundance of gut microbiota between AMR and control groups at the species level. Table S3. Comparison of relative abundance of gut microbiota between AMR and control groups at the phylum level. Table S4. Comparison of relative abundance of gut microbiota between AMR and control groups at the genus level. Table S5. Comparison of relative abundance of KOs between AMR and control groups. Table S6. Interrelationship between AMR-associated gut microbial species, functions and metabolites. Table S7. Spearman correlation between species and clinical indicators. Table S8. Spearman correlation between metabolites and clinical indicators. Table S9. Area under the ROC curves of microbial biomarkers. Table S10. Antibiotic resistance and virulence genes of key species associated with AMR after kidney transplantation. Table S11. Area under the ROC curves of metabolic biomarkers [file 12967_2022_3825_MOESM1_ESM.docx]

**Additional file**

**Table S1. Histopathological characteristics according to the Banff 2019 criteria of AMR cases**

|  | **g-score** | **i-score** | **t-score** | **v-score** | **ah-score** | **cg-score** | **mm-score** | **ci-score** | **ct-score** | **cv-score** | **ptc-score** | **C4d-score** |
| --- | --- | --- | --- | --- | --- | --- | --- | --- | --- | --- | --- | --- |
| **AMR 1** | 3 | 1 | 0 | 0 | 1 | 2 | 1 | 1 | 1 | 1 | 2 | 1 |
| **AMR 2** | 2 | 1 | 0 | 0 | 1 | 1 | 0 | 1 | 0 | 0 | 1 | 2 |
| **AMR 3** | 3 | 1 | 1 | 0 | 1 | 2 | 2 | 1 | 1 | 1 | 2 | 2 |
| **AMR 4** | 2 | 1 | 1 | 0 | 0 | 1 | 1 | 0 | 0 | 1 | 1 | 1 |
| **AMR 5** | 1 | 1 | 1 | 0 | 2 | 3 | 1 | 1 | 1 | 2 | 1 | 2 |
| **AMR 6** | 2 | 1 | 0 | 0 | 1 | 3 | 2 | 2 | 1 | 2 | 1 | 1 |
| **AMR 7** | 3 | 2 | 0 | 0 | 1 | 2 | 1 | 1 | 1 | 0 | 3 | 2 |
| **AMR 8** | 2 | 1 | 0 | 1 | 2 | 3 | 3 | 3 | 2 | 2 | 1 | 1 |
| **AMR 9** | 1 | 0 | 0 | 0 | 1 | 3 | 2 | 2 | 2 | 2 | 1 | 0 |
| **AMR 10** | 3 | 1 | 0 | 0 | 1 | 3 | 2 | 3 | 2 | 1 | 2 | 1 |
| **AMR 11** | 2 | 1 | 1 | 1 | 2 | 3 | 2 | 2 | 2 | 2 | 2 | 2 |
| **AMR 12** | 3 | 2 | 0 | 0 | 1 | 2 | 1 | 1 | 1 | 1 | 2 | 2 |
| **AMR 13** | 2 | 2 | 0 | 0 | 1 | 1 | 0 | 0 | 1 | 1 | 2 | 2 |
| **AMR 14** | 2 | 1 | 1 | 0 | 2 | 3 | 2 | 2 | 1 | 2 | 0 | 2 |
| **AMR 15** | 1 | 1 | 2 | 0 | 2 | 3 | 3 | 1 | 1 | 2 | 1 | 1 |
| **AMR 16** | 3 | 1 | 1 | 0 | 0 | 1 | 0 | 1 | 1 | 0 | 2 | 3 |
| **AMR 17** | 1 | 1 | 0 | 0 | 1 | 3 | 1 | 1 | 1 | 2 | 1 | 1 |
| **AMR 18** | 2 | 1 | 0 | 0 | 2 | 3 | 2 | 2 | 1 | 2 | 1 | 1 |
| **AMR 19** | 3 | 1 | 0 | 0 | 2 | 2 | 2 | 1 | 1 | 1 | 2 | 2 |
| **AMR 20** | 1 | 0 | 0 | 0 | 1 | 2 | 3 | 1 | 1 | 1 | 2 | 0 |
| **AMR 21** | 1 | 1 | 1 | 1 | 2 | 3 | 2 | 2 | 2 | 2 | 1 | 1 |
| **AMR 22** | 2 | 1 | 0 | 0 | 1 | 2 | 1 | 1 | 1 | 1 | 1 | 2 |
| **AMR 23** | 3 | 3 | 1 | 0 | 1 | 2 | 1 | 1 | 1 | 1 | 2 | 3 |
| **AMR 24** | 2 | 1 | 1 | 0 | 1 | 2 | 1 | 1 | 0 | 1 | 1 | 1 |
| **AMR 25** | 1 | 1 | 0 | 0 | 2 | 3 | 2 | 1 | 1 | 2 | 2 | 0 |
| **AMR 26** | 3 | 1 | 1 | 0 | 0 | 1 | 0 | 0 | 0 | 0 | 2 | 2 |
| **AMR 27** | 3 | 2 | 0 | 0 | 1 | 2 | 1 | 1 | 1 | 2 | 3 | 1 |
| **AMR 28** | 2 | 2 | 0 | 1 | 1 | 2 | 1 | 1 | 1 | 1 | 2 | 1 |

g, glomerulitis; i, interstitial inflammation; t, tubulitis; v, intimal artertitis; ah, arteriolar hyaline thickening; cg, glomerulopathy; mm, mesangial matrix; ci, interstitial fibrosis; ct, tubular atrophy; cv, vascular fibrosis; ptc, peritubular capillaritis.

**Table S2. Comparison of relative abundance of gut microbiota between AMR and control groups at the species level**

| **Species** | ***P* value** | **FDR-adjusted**  ***P* value** | **Fold change**  **(AMR vs Ctrl)** |  |
| --- | --- | --- | --- | --- |
| *Faecalibacterium prausnitzii* | 0.0020 | 0.0188 | 0.3590 | |
| *[Eubacterium] rectale* | 0.0005 | 0.0153 | 0.2069 | |
| *[Ruminococcus] torques* | 0.0051 | 0.0240 | 0.6643 | |
| *Coprococcus catus* | 0.0013 | 0.0161 | 0.5619 | |
| *Bifidobacterium pseudocatenulatum* | 0.0357 | 0.0439 | 0.1090 | |
| *Lactobacillus fermentum* | 0.0459 | 0.0486 | 106.5148 | |
| *Coprococcus sp. ART55/1* | 0.0116 | 0.0318 | 0.3465 | |
| *Ruminococcus bicirculans* | 0.0131 | 0.0339 | 0.6556 | |
| *Dialister sp. Marseille-P5638* | 0.0008 | 0.0153 | 0.0489 | |
| *[Eubacterium] siraeum* | 0.0289 | 0.0409 | 0.4807 | |
| *Bacteroides heparinolyticus* | 0.0246 | 0.0398 | 0.5481 | |
| *Barnesiella viscericola* | 0.0009 | 0.0153 | 0.2830 | |
| *Desulfovibrio piger* | 0.0246 | 0.0398 | 0.6018 | |
| *[Eubacterium] sulci* | 0.0080 | 0.0271 | 0.5851 | |
| *Bifidobacterium breve* | 0.0274 | 0.0403 | 0.4631 | |
| *Ruminococcus champanellensis* | 0.0437 | 0.0474 | 0.4633 | |
| *Lactobacillus johnsonii* | 0.0482 | 0.0493 | 3.7028 | |
| *Acidaminococcus fermentans* | 0.0437 | 0.0474 | 0.6658 | |
| *Bifidobacterium kashiwanohense* | 0.0027 | 0.0201 | 0.1732 | |
| *Phoenicibacter massiliensis* | 0.0006 | 0.0153 | 0.5235 | |
| *Bifidobacterium adolescentis* | 0.0012 | 0.0161 | 0.2529 | |
| *Faecalibacterium sp.* | 0.0050 | 0.0240 | 0.3774 | |
| *Tannerella forsythia* | 0.0007 | 0.0153 | 0.3555 | |
| *Bifidobacterium bifidum* | 0.0233 | 0.0398 | 0.4765 | |
| *Slackia heliotrinireducens* | 0.0047 | 0.0240 | 0.3137 | |
| *Libanicoccus massiliensis* | 0.0220 | 0.0383 | 0.4206 | |
| *Prevotella dentalis* | 0.0097 | 0.0300 | 0.5903 | |
| *Klebsiella phage K5-4* | 0.0376 | 0.0439 | 0.0006 | |
| *Prevotella ruminicola* | 0.0005 | 0.0153 | 0.2994 | |
| *Gordonibacter pamelaeae* | 0.0176 | 0.0362 | 0.5388 | |
| *Lactobacillus acidophilus* | 0.0459 | 0.0486 | 15.3669 | |
| *Olsenella umbonata* | 0.0091 | 0.0292 | 0.4919 | |
| *Tannerella sp. oral taxon HOT-286* | 0.0339 | 0.0436 | 0.5249 | |
| *Prevotella fusca* | 0.0044 | 0.0239 | 0.5752 | |
| *Gordonibacter urolithinfaciens* | 0.0197 | 0.0377 | 0.3382 | |
| *Parvimonas micra* | 0.0274 | 0.0403 | 0.3996 | |
| *Mogibacterium pumilum* | 0.0166 | 0.0352 | 0.6494 | |
| *Myroides odoratimimus* | 0.0066 | 0.0243 | 0.5770 | |
| *Eggerthella sp. YY7918* | 0.0395 | 0.0447 | 0.6662 | |
| *Prevotella melaninogenica* | 0.0305 | 0.0419 | 0.5160 | |
| *Paenibacillus mucilaginosus* | 0.0395 | 0.0447 | 0.6180 | |
| *Bifidobacterium scardovii* | 0.0025 | 0.0201 | 0.4682 | |
| *Bifidobacterium catenulatum* | 0.0274 | 0.0403 | 0.2241 | |
| *Faecalibacterium phage FP_Taranis* | 0.0119 | 0.0319 | 0.2524 | |
| *Serratia marcescens* | 0.0047 | 0.0240 | 0.5353 | |
| *Petrimonas sp. IBARAKI* | 0.0149 | 0.0351 | 0.5039 | |
| *Fermentimonas caenicola* | 0.0339 | 0.0436 | 0.5423 | |
| *Faecalibacterium phage FP_Lugh* | 0.0034 | 0.0221 | 0.0541 | |
| *Prevotella enoeca* | 0.0246 | 0.0398 | 0.2702 | |
| *Pseudopedobacter saltans* | 0.0043 | 0.0239 | 0.4954 | |
| *Pseudomonas putida* | 0.0166 | 0.0352 | 0.5539 | |
| *Prevotella jejuni* | 0.0009 | 0.0153 | 0.3325 | |
| *Faecalibacterium phage FP_Toutatis* | 0.0020 | 0.0188 | 0.1408 | |
| *Acidovorax avenae* | 0.0062 | 0.0243 | 0.6094 | |
| *Fastidiosipila sanguinis* | 0.0395 | 0.0447 | 0.3409 | |
| *Thermaerobacter marianensis* | 0.0339 | 0.0436 | 0.4814 | |
| *Brachyspira pilosicoli* | 0.0004 | 0.0153 | 0.4554 | |
| *Actinomyces succiniciruminis* | 0.0025 | 0.0201 | 0.5121 | |
| *[Eubacterium] minutum* | 0.0375 | 0.0439 | 0.5803 | |
| *Moorella thermoacetica* | 0.0395 | 0.0447 | 0.5597 | |
| *Desulfitobacterium metallireducens* | 0.0437 | 0.0474 | 0.5547 | |
| *Chitinophaga pinensis* | 0.0357 | 0.0439 | 0.5066 | |
| *Denitrobacterium detoxificans* | 0.0305 | 0.0419 | 0.4668 | |
| *Olsenella sp. oral taxon 807* | 0.0186 | 0.0371 | 0.4817 | |
| *Bacillus oceanisediminis* | 0.0022 | 0.0192 | 0.5421 | |
| *Bifidobacterium angulatum* | 0.0014 | 0.0161 | 0.3937 | |
| *Fusobacterium gonidiaformans* | 0.0149 | 0.0351 | 0.5416 | |
| *Faecalibacterium phage FP_oengus* | 0.0054 | 0.0240 | 0.4241 | |
| *Deinococcus proteolyticus* | 0.0026 | 0.0201 | 0.3422 | |
| *Rufibacter tibetensis* | 0.0048 | 0.0240 | 0.5941 | |
| *Thermincola potens* | 0.0103 | 0.0304 | 0.5813 | |
| *Vibrio tritonius* | 0.0500 | 0.0500 | 0.6468 | |
| *Pelobacter carbinolicus* | 0.0357 | 0.0439 | 0.5591 | |
| *Paenibacillus kribbensis* | 0.0375 | 0.0439 | 0.6063 | |
| *Flavobacterium gilvum* | 0.0201 | 0.0377 | 0.3712 | |
| *Stigmatella aurantiaca* | 0.0006 | 0.0153 | 2.1373 | |
| *Mucilaginibacter sp. PAMC 26640* | 0.0027 | 0.0201 | 0.3803 | |
| *Bacillus sp. OxB-1* | 0.0031 | 0.0204 | 0.5520 | |
| *Alkalitalea saponilacus* | 0.0051 | 0.0240 | 0.4761 | |
| *Pasteurellaceae bacterium NI1060* | 0.0081 | 0.0272 | 0.4652 | |
| *Sphingobacterium sp. G1-14* | 0.0360 | 0.0439 | 0.2336 | |
| *Azospira oryzae* | 0.0207 | 0.0377 | 0.6278 | |
| *Propionibacterium freudenreichii* | 0.0019 | 0.0188 | 0.3819 | |
| *Desulfovibrio salexigens* | 0.0045 | 0.0239 | 0.3069 | |
| *Oxalobacter formigenes* | 0.0482 | 0.0493 | 0.3618 | |
| *Mahella australiensis* | 0.0058 | 0.0241 | 0.6014 | |
| *Enterobacter cloacae complex sp.* | 0.0463 | 0.0486 | 0.3342 | |
| *Elizabethkingia anophelis* | 0.0260 | 0.0403 | 0.5819 | |
| *Haliscomenobacter hydrossis* | 0.0482 | 0.0493 | 0.4923 | |
| *Bifidobacterium choerinum* | 0.0002 | 0.0153 | 0.2271 | |
| *Bifidobacterium dentium* | 0.0054 | 0.0240 | 0.2044 | |
| *Ottowia sp. oral taxon 894* | 0.0053 | 0.0240 | 0.2988 | |
| *Caldicellulosiruptor saccharolyticus* | 0.0041 | 0.0239 | 0.5051 | |
| *Neomicrococcus aestuarii* | 0.0242 | 0.0398 | 0.2279 | |
| *Runella slithyformis* | 0.0207 | 0.0377 | 0.4604 | |
| *Pseudomonas citronellolis* | 0.0357 | 0.0439 | 0.5855 | |
| *Flavobacterium commune* | 0.0330 | 0.0434 | 0.5076 | |
| *Rhodothermus marinus* | 0.0466 | 0.0486 | 0.4715 | |
| *Desulfobacula toluolica* | 0.0305 | 0.0419 | 0.6594 | |
| *Actinomyces sp. Chiba101* | 0.0314 | 0.0419 | 0.6580 | |
| *Rhodoferax ferrireducens* | 0.0066 | 0.0243 | 0.4152 | |
| *Thermoanaerobacter kivui* | 0.0220 | 0.0383 | 0.6443 | |
| *Luteitalea pratensis* | 0.0070 | 0.0253 | 0.5560 | |
| *Gramella sp. MAR_2010_147* | 0.0252 | 0.0398 | 0.2316 | |
| *Candidatus Arthromitus sp. SFB-rat-Yit* | 0.0207 | 0.0377 | 0.6073 | |
| *Serratia grimesii* | 0.0089 | 0.0290 | 0.5688 | |
| *Lactobacillus koreensis* | 0.0186 | 0.0371 | 0.5934 | |
| *Actinomyces radingae* | 0.0124 | 0.0323 | 0.5601 | |
| *Actinobacillus suis* | 0.0347 | 0.0439 | 0.4642 | |
| *Collimonas fungivorans* | 0.0149 | 0.0351 | 0.5581 | |
| *Mucilaginibacter mallensis* | 0.0061 | 0.0243 | 0.3980 | |
| *Comamonas kerstersii* | 0.0136 | 0.0341 | 0.4533 | |
| *Hymenobacter sp. PAMC 26628* | 0.0008 | 0.0153 | 0.2638 | |
| *Kluyvera georgiana* | 0.0045 | 0.0239 | 0.3875 | |
| *Acinetobacter sp. TGL-Y2* | 0.0055 | 0.0240 | 0.2570 | |
| *Pantoea sp. PSNIH1* | 0.0498 | 0.0500 | 0.3593 | |
| *Azotobacter chroococcum* | 0.0260 | 0.0403 | 0.5991 | |
| *Cryobacterium arcticum* | 0.0014 | 0.0161 | 0.2239 | |
| *Hymenobacter sp. APR13* | 0.0203 | 0.0377 | 0.3177 | |
| *Erysipelotrichaceae bacterium I46* | 0.0217 | 0.0382 | 5.0457 | |
| *Plesiomonas shigelloides* | 0.0112 | 0.0312 | 0.3805 | |
| *Mucilaginibacter gotjawali* | 0.0014 | 0.0161 | 0.2570 | |
| *Hymenobacter sp. PAMC 26554* | 0.0065 | 0.0243 | 0.3281 | |
| *Paludisphaera borealis* | 0.0160 | 0.0352 | 0.1587 | |
| *Muricauda ruestringensis* | 0.0461 | 0.0486 | 0.2452 | |
| *Planococcus sp. PAMC 21323* | 0.0291 | 0.0409 | 0.6046 | |
| *Granulosicoccus antarcticus* | 0.0412 | 0.0458 | 0.5559 | |
| *Capnocytophaga stomatis* | 0.0309 | 0.0419 | 0.4863 | |
| *Streptococcus himalayensis* | 0.0274 | 0.0403 | 0.5738 | |
| *Rhodoferax antarcticus* | 0.0166 | 0.0352 | 0.5609 | |
| *Synechococcus sp. JA-2-3B'a(2-13)* | 0.0057 | 0.0240 | 0.3452 | |
| *Hydrogenophaga crassostreae* | 0.0356 | 0.0439 | 0.3326 | |
| *Hymenobacter sedentarius* | 0.0094 | 0.0296 | 0.3672 | |
| *Trichomonas vaginalis* | 0.0090 | 0.0292 | 0.3973 | |
| *Clostridium sp. enrichment culture clone 7-14* | 0.0084 | 0.0276 | 0.5632 | |
| *Acidovorax sp. RAC01* | 0.0015 | 0.0166 | 0.1884 | |
| *Halomonas sp. HG01* | 0.0450 | 0.0483 | 0.4180 | |
| *Sneathia amnii* | 0.0185 | 0.0371 | 0.5721 | |
| *Halomonas sp. 1513* | 0.0366 | 0.0439 | 0.5810 | |
| *Kiritimatiella glycovorans* | 0.0185 | 0.0371 | 0.5152 | |
| *Spirosoma linguale* | 0.0251 | 0.0398 | 0.2950 | |
| *Bordetella bronchiseptica* | 0.0056 | 0.0240 | 0.5008 | |
| *Isoptericola dokdonensis* | 0.0357 | 0.0439 | 0.6344 | |
| *Cellulomonas fimi* | 0.0466 | 0.0486 | 0.5841 | |
| *Kyrpidia sp. EA-1* | 0.0294 | 0.0410 | 0.4893 | |
| *Caldicellulosiruptor hydrothermalis* | 0.0375 | 0.0439 | 0.6286 | |
| *Geobacter metallireducens* | 0.0053 | 0.0240 | 0.3376 | |
| *Acidovorax carolinensis* | 0.0009 | 0.0153 | 0.2988 | |
| *Geobacillus sp. WCH70* | 0.0433 | 0.0474 | 0.6560 | |
| *Gemmatirosa kalamazoonesis* | 0.0013 | 0.0161 | 0.3088 | |
| *Planococcus sp. MB-3u-03* | 0.0003 | 0.0153 | 0.2919 | |
| *Pelodictyon luteolum* | 0.0487 | 0.0495 | 0.5504 | |
| *Gallibacterium anatis* | 0.0157 | 0.0352 | 0.5277 | |
| *Xanthomonas oryzae* | 0.0029 | 0.0203 | 0.5464 | |
| *Corynebacterium imitans* | 0.0210 | 0.0377 | 0.5076 | |
| *Rhodoferax koreense* | 0.0196 | 0.0377 | 0.5076 | |
| *Caldithrix abyssi* | 0.0171 | 0.0358 | 2.8203 | |
| *Stenotrophomonas acidaminiphila* | 0.0438 | 0.0474 | 0.5498 | |
| *Flavobacterium sp. HYN0048* | 0.0349 | 0.0439 | 0.3662 | |
| *Acidithiobacillus caldus* | 0.0279 | 0.0403 | 0.2963 | |
| *Aureitalea sp. RR4-38* | 0.0121 | 0.0320 | 0.2831 | |
| *Spirosoma rigui* | 0.0098 | 0.0300 | 0.3359 | |
| *Sterolibacterium denitrificans* | 0.0118 | 0.0318 | 0.4807 | |
| *Syntrophus aciditrophicus* | 0.0269 | 0.0403 | 0.5966 | |
| *Aeromonas sp. CU5* | 0.0238 | 0.0398 | 0.3377 | |
| *Achromobacter sp. AONIH1* | 0.0269 | 0.0403 | 0.6144 | |
| *Salmonella bongori* | 0.0029 | 0.0203 | 0.3751 | |
| *Methanocorpusculum labreanum* | 0.0049 | 0.0240 | 0.3717 | |
| *Enterococcus avium* | 0.0250 | 0.0398 | 2.5029 | |
| *Pontibacter akesuensis* | 0.0485 | 0.0495 | 0.6502 | |
| *Halomonas hydrothermalis* | 0.0114 | 0.0313 | 2.3153 | |
| *Photobacterium gaetbulicola* | 0.0211 | 0.0377 | 0.4490 | |
| *Grimontia hollisae* | 0.0194 | 0.0377 | 0.5217 | |
| *Plantibacter flavus* | 0.0021 | 0.0188 | 0.4192 | |
| *Corynebacterium sphenisci* | 0.0080 | 0.0271 | 0.4581 | |
| *Verminephrobacter eiseniae* | 0.0321 | 0.0426 | 0.4293 | |
| *Caldicellulosiruptor bescii* | 0.0313 | 0.0419 | 0.5281 | |
| *Rhizobium sp. ACO-34A* | 0.0073 | 0.0259 | 0.6195 | |
| *endosymbiont of unidentified scaly snail isolate Monju* | 0.0039 | 0.0239 | 0.3729 | |
| *Vibrio crassostreae* | 0.0136 | 0.0341 | 0.3978 | |
| *Virgibacillus phasianinus* | 0.0344 | 0.0439 | 0.4428 | |
| *Stenotrophomonas sp. LM091* | 0.0464 | 0.0486 | 0.6554 | |
| *Thermus thermophilus* | 0.0489 | 0.0495 | 0.5158 | |
| *Micromonospora auratinigra* | 0.0136 | 0.0341 | 0.1362 | |
| *Corynebacterium frankenforstense* | 0.0256 | 0.0402 | 0.5672 | |
| *Fluviicola taffensis* | 0.0070 | 0.0253 | 0.4263 | |
| *Tessaracoccus sp. T2.5-30* | 0.0042 | 0.0239 | 0.5701 | |
| *Bradyrhizobium sp. BTAi1* | 0.0376 | 0.0439 | 0.1184 | |
| *Thermoanaerobacterium saccharolyticum* | 0.0279 | 0.0403 | 0.3804 | |
| *Novosphingobium resinovorum* | 0.0402 | 0.0450 | 0.5182 | |
| *Paraburkholderia sprentiae* | 0.0157 | 0.0352 | 0.5035 | |
| *Myxococcus stipitatus* | 0.0146 | 0.0351 | 0.4364 | |
| *Methyloversatilis sp. RAC08* | 0.0412 | 0.0458 | 0.5960 | |
| *Conexibacter woesei* | 0.0103 | 0.0304 | 0.3985 | |
| *Brevundimonas naejangsanensis* | 0.0151 | 0.0352 | 0.3875 | |
| *Pectobacterium polaris* | 0.0475 | 0.0493 | 0.5884 | |
| *Cutibacterium acnes* | 0.0141 | 0.0344 | 0.5137 | |
| *Roseiflexus castenholzii* | 0.0373 | 0.0439 | 0.5831 | |
| *Caulobacter mirabilis* | 0.0062 | 0.0243 | 0.5254 | |
| *Arenibacter algicola* | 0.0265 | 0.0403 | 0.5382 | |
| *Terrisporobacter glycolicus* | 0.0400 | 0.0449 | 0.3876 | |
| *Brachybacterium sp. VM2412* | 0.0373 | 0.0439 | 0.5706 | |
| *Desulfotalea psychrophila* | 0.0016 | 0.0172 | 0.3925 | |
| *Variovorax sp. PAMC 28711* | 0.0290 | 0.0409 | 0.5923 | |
| *Polaribacter vadi* | 0.0177 | 0.0363 | 0.2023 | |
| *Janibacter indicus* | 0.0275 | 0.0403 | 0.5083 | |
| *Martelella mediterranea* | 0.0011 | 0.0161 | 0.3463 | |
| *Vibrio natriegens* | 0.0400 | 0.0449 | 0.1289 | |
| *Vibrio owensii* | 0.0313 | 0.0419 | 0.5888 | |
| *Salipiger profundus* | 0.0312 | 0.0419 | 2.2233 | |
| *Basilea psittacipulmonis* | 0.0328 | 0.0434 | 0.5653 | |
| *Streptomyces violaceoruber* | 0.0148 | 0.0351 | 0.3181 | |
| *Devosia sp. H5989* | 0.0057 | 0.0240 | 0.4408 | |
| *Gloeobacter violaceus* | 0.0207 | 0.0377 | 0.4019 | |
| *Methylobacterium nodulans* | 0.0331 | 0.0434 | 0.4959 | |
| *Prosthecochloris aestuarii* | 0.0163 | 0.0352 | 0.3414 | |
| *Methylosinus trichosporium* | 0.0104 | 0.0304 | 0.5433 | |
| *Thermoplasmatales archaeon BRNA1* | 0.0003 | 0.0153 | 0.2686 | |
| *Xanthomonas sacchari* | 0.0159 | 0.0352 | 0.5220 | |
| *Halobacillus mangrovi* | 0.0040 | 0.0239 | 0.5045 | |
| *Lactobacillus terrae* | 0.0499 | 0.0500 | 0.2750 | |
| *Corynebacterium uterequi* | 0.0212 | 0.0377 | 0.5447 | |
| *Burkholderia ubonensis* | 0.0374 | 0.0439 | 0.4322 | |
| *Geobacter daltonii* | 0.0206 | 0.0377 | 0.5003 | |
| *Gordonia iterans* | 0.0210 | 0.0377 | 0.5509 | |
| *Chromohalobacter salexigens* | 0.0200 | 0.0377 | 0.4505 | |
| *Phreatobacter cathodiphilus* | 0.0333 | 0.0435 | 0.4093 | |
| *Piscirickettsia salmonis* | 0.0158 | 0.0352 | 3.0272 | |
| *Aeromonas schubertii* | 0.0378 | 0.0439 | 0.3533 | |
| *Agrobacterium rhizogenes* | 0.0166 | 0.0352 | 0.6094 | |
| *Alcanivorax dieselolei* | 0.0231 | 0.0398 | 0.2753 | |
| *Octadecabacter arcticus* | 0.0241 | 0.0398 | 0.5140 | |
| *Sulfurifustis variabilis* | 0.0030 | 0.0203 | 0.3566 | |
| *Oceanithermus profundus* | 0.0385 | 0.0443 | 0.3637 | |
| *Arthrobacter sp. YC-RL1* | 0.0062 | 0.0243 | 0.1553 | |
| *Candidatus Nanopelagicus abundans* | 0.0280 | 0.0403 | 0.5946 | |
| *Intrasporangium calvum* | 0.0446 | 0.0480 | 2.7496 | |
| *Geobacter uraniireducens* | 0.0443 | 0.0478 | 0.4590 | |
| *Acidovorax cattleyae* | 0.0235 | 0.0398 | 0.5443 | |
| *Xylella fastidiosa* | 0.0111 | 0.0312 | 0.5201 | |
| *Ruegeria sp. TM1040* | 0.0284 | 0.0407 | 0.2777 | |
| *Microbacterium aurum* | 0.0039 | 0.0239 | 0.3258 | |
| *Rhodococcus opacus* | 0.0389 | 0.0447 | 0.4051 | |
| *Shewanella baltica* | 0.0265 | 0.0403 | 0.5381 | |
| *Pseudoalteromonas piscicida* | 0.0374 | 0.0439 | 0.6629 | |
| *Pantoea gaviniae* | 0.0030 | 0.0203 | 0.5980 | |
| *Cladophialophora bantiana* | 0.0370 | 0.0439 | 4.1981 | |
| *Friedmanniella sagamiharensis* | 0.0189 | 0.0373 | 0.3677 | |
| *Agromyces sp. 30A* | 0.0080 | 0.0271 | 0.4467 | |
| *Fervidobacterium pennivorans* | 0.0285 | 0.0407 | 0.4330 | |
| *Sphingomonas sp. JJ-A5* | 0.0472 | 0.0491 | 0.5954 | |
| *Anaerolinea thermophila* | 0.0277 | 0.0403 | 0.6516 | |
| *Salinicola tamaricis* | 0.0273 | 0.0403 | 0.5787 | |
| *Aspergillus aculeatus* | 0.0013 | 0.0161 | 0.0110 | |
| *Mycolicibacterium thermoresistibile* | 0.0022 | 0.0192 | 0.4428 | |
| *Candidatus Pelagibacter ubique* | 0.0093 | 0.0296 | 0.4952 | |
| *Francisella philomiragia* | 0.0062 | 0.0243 | 0.4752 | |
| *Mycolicibacterium rutilum* | 0.0198 | 0.0377 | 0.5798 | |
| *Calothrix sp. NIES-2100* | 0.0192 | 0.0375 | 0.3385 | |
| *Streptomyces lincolnensis* | 0.0001 | 0.0153 | 0.2402 | |
| *Vibrio campbellii* | 0.0079 | 0.0271 | 0.4443 | |
| *Bacteroidetes bacterium UKL13-3* | 0.0345 | 0.0439 | 0.5415 | |
| *Phycisphaera mikurensis* | 0.0490 | 0.0495 | 0.2872 | |
| *Thermotoga sp. RQ7* | 0.0240 | 0.0398 | 0.1863 | |
| *Bartonella vinsonii* | 0.0111 | 0.0312 | 0.5386 | |
| *Xanthomonas phaseoli* | 0.0339 | 0.0436 | 0.4356 | |
| *Nocardia terpenica* | 0.0252 | 0.0398 | 0.4805 | |
| *Corynebacterium humireducens* | 0.0045 | 0.0239 | 0.3317 | |
| *Frondihabitans sp. 762G35* | 0.0064 | 0.0243 | 0.4540 | |
| *Labrenzia sp. VG12* | 0.0366 | 0.0439 | 0.5015 | |
| *Legionella lansingensis* | 0.0271 | 0.0403 | 0.4875 | |
| *Clavibacter michiganensis* | 0.0454 | 0.0485 | 0.5504 | |
| *Dictyoglomus turgidum* | 0.0019 | 0.0188 | 0.6553 | |
| *Pseudomonas guangdongensis* | 0.0275 | 0.0403 | 0.5000 | |
| *Candidatus Phytoplasma australiense* | 0.0132 | 0.0339 | 2.4453 | |
| *[Mycobacterium] stephanolepidis* | 0.0173 | 0.0358 | 4.1269 | |
| *Corynebacterium glyciniphilum* | 0.0102 | 0.0304 | 0.3528 | |
| *Enterococcus sp. CR-Ec1* | 0.0416 | 0.0460 | 5.8909 | |
| *Paenibacillus alvei* | 0.0120 | 0.0319 | 0.0312 | |
| *Acetobacter ghanensis* | 0.0310 | 0.0419 | 0.3759 | |
| *Plantactinospora sp. KBS50* | 0.0160 | 0.0352 | 0.3976 | |
| *Pseudomonas fulva* | 0.0002 | 0.0153 | 0.2406 | |
| *Streptomyces gilvosporeus* | 0.0107 | 0.0310 | 0.3836 | |
| *Rhodococcus virus Pepy6* | 0.0237 | 0.0398 | 6.5119 | |
| *Dokdonia sp. 4H-3-7-5* | 0.0171 | 0.0358 | 0.4106 | |
| *Actinoplanes sp. N902-109* | 0.0356 | 0.0439 | 0.3350 | |
| *Mycobacterium sp. djl-10* | 0.0137 | 0.0341 | 0.5254 | |
| *Streptomyces sp. SF2575* | 0.0165 | 0.0352 | 8.1914 | |
| *Micromonospora viridifaciens* | 0.0023 | 0.0192 | 0.4012 | |
| *Ketobacter alkanivorans* | 0.0111 | 0.0312 | 0.4727 | |
| *Agrococcus jejuensis* | 0.0293 | 0.0410 | 0.2911 | |
| *Polaribacter sp. KT25b* | 0.0138 | 0.0341 | 0.2281 | |
| *Agrococcus carbonis* | 0.0166 | 0.0352 | 0.3091 | |
| *Yersinia rohdei* | 0.0252 | 0.0398 | 0.5723 | |
| *Pelagibaca abyssi* | 0.0097 | 0.0300 | 0.2432 | |
| *Sinomonas sp. R1AF57* | 0.0043 | 0.0239 | 0.4096 | |
| *Clostridiales bacterium CoAT_53-4c* | 0.0005 | 0.0153 | 0.1958 | |
| *Synechococcus sp. SynAce01* | 0.0246 | 0.0398 | 0.4179 | |
| *Lysinibacillus fusiformis* | 0.0161 | 0.0352 | 0.5391 | |
| *Candidatus Symbiobacter mobilis* | 0.0079 | 0.0271 | 0.3067 | |
| *Thauera aromatica* | 0.0108 | 0.0310 | 0.2059 | |
| *Campylobacter insulaenigrae* | 0.0309 | 0.0419 | 1.7784 | |
| *Jiangella sp. DSM 45060* | 0.0187 | 0.0371 | 0.4138 | |
| *Jatrophihabitans sp. GAS493* | 0.0214 | 0.0378 | 0.4460 | |
| *Enterococcus gallinarum* | 0.0479 | 0.0493 | 5.4110 | |
| *Aeromonas sp. ASNIH4* | 0.0382 | 0.0441 | 0.4904 | |
| *Corynebacterium glutamicum* | 0.0250 | 0.0398 | 0.5365 | |
| *Acutalibacter muris* | 0.0003 | 0.0153 | 0.1568 | |
| *Microbacterium sp. XT11* | 0.0007 | 0.0153 | 0.3105 | |
| *Streptomyces glaucescens* | 0.0378 | 0.0439 | 0.3576 | |
| *Porphyrobacter HT-58-2* | 0.0421 | 0.0465 | 0.5310 | |

**Table S3. Comparison of relative abundance of gut microbiota between AMR and control groups at the phylum level**

| **Phylum** | ***P* value** | **FDR-adjusted**  ***P* value** | **Fold change (AMR vs Ctrl)** |
| --- | --- | --- | --- |
| *Gemmatimonadetes* | 0.0011 | 0.0074 | 0.4086 |
| *Thermodesulfobacteria* | 0.0217 | 0.0287 | 0.4167 |
| *Kiritimatiellaeota* | 0.0185 | 0.0287 | 0.5152 |
| *Chlorobi* | 0.0139 | 0.0287 | 0.5351 |
| *Actinobacteria* | 0.0246 | 0.0287 | 0.5955 |
| *Acidobacteria* | 0.0305 | 0.0305 | 0.6063 |
| *Calditrichaeota* | 0.0171 | 0.0287 | 2.8203 |

**Table S4. Comparison of relative abundance of gut microbiota between AMR and control groups at the genus level**

| **Genus** | ***P* value** | **FDR-adjusted**  ***P* value** | **Fold change (AMR vs Ctrl)** |
| --- | --- | --- | --- |
| *Neomicrococcus* | 0.0242 | 0.0407 | 0.2279 |
| *Agrococcus* | 0.0491 | 0.0491 | 0.2999 |
| *Slackia* | 0.0047 | 0.0279 | 0.3128 |
| *Cryobacterium* | 0.0133 | 0.0365 | 0.3773 |
| *Plantactinospora* | 0.0108 | 0.0365 | 0.3891 |
| *Propionibacterium* | 0.0020 | 0.0201 | 0.3903 |
| *Conexibacter* | 0.0103 | 0.0365 | 0.3985 |
| *Friedmanniella* | 0.0423 | 0.0453 | 0.4167 |
| *Plantibacter* | 0.0021 | 0.0201 | 0.4192 |
| *Libanicoccus* | 0.0220 | 0.0403 | 0.4206 |
| *Jiangella* | 0.0240 | 0.0407 | 0.4421 |
| *Jatrophihabitans* | 0.0214 | 0.0401 | 0.4460 |
| *Denitrobacterium* | 0.0233 | 0.0404 | 0.4510 |
| *Olsenella* | 0.0124 | 0.0365 | 0.4689 |
| *Leifsonia* | 0.0284 | 0.0407 | 0.4933 |
| *Kocuria* | 0.0233 | 0.0404 | 0.4972 |
| *Frondihabitans* | 0.0256 | 0.0407 | 0.5079 |
| *Janibacter* | 0.0275 | 0.0407 | 0.5083 |
| *Bifidobacterium* | 0.0166 | 0.0378 | 0.5130 |
| *Cellulosimicrobium* | 0.0256 | 0.0407 | 0.5147 |
| *Agromyces* | 0.0181 | 0.0385 | 0.5162 |
| *Phoenicibacter* | 0.0006 | 0.0192 | 0.5235 |
| *Gordonibacter* | 0.0091 | 0.0365 | 0.5319 |
| *Curtobacterium* | 0.0454 | 0.0475 | 0.5561 |
| *Sinomonas* | 0.0464 | 0.0476 | 0.5681 |
| *Rubrobacter* | 0.0385 | 0.0451 | 0.5984 |
| *Mycobacterium* | 0.0289 | 0.0407 | 0.6043 |
| *Candidatus Nanopelagicus* | 0.0312 | 0.0407 | 0.6151 |
| *Pseudonocardia* | 0.0322 | 0.0411 | 0.6614 |
| *Intrasporangium* | 0.0446 | 0.0470 | 2.7496 |
| *Barnesiella* | 0.0009 | 0.0198 | 0.2825 |
| *Aureitalea* | 0.0121 | 0.0365 | 0.2831 |
| *Muricauda* | 0.0312 | 0.0407 | 0.3519 |
| *Gramella* | 0.0029 | 0.0222 | 0.3615 |
| *Tannerella* | 0.0019 | 0.0201 | 0.4056 |
| *Hymenobacter* | 0.0006 | 0.0192 | 0.4155 |
| *Dokdonia* | 0.0125 | 0.0365 | 0.4385 |
| *Mucilaginibacter* | 0.0025 | 0.0222 | 0.4397 |
| *Fluviicola* | 0.0112 | 0.0365 | 0.4490 |
| *Runella* | 0.0207 | 0.0394 | 0.4604 |
| *Rhodothermus* | 0.0466 | 0.0476 | 0.4715 |
| *Alkalitalea* | 0.0051 | 0.0281 | 0.4761 |
| *Spirosoma* | 0.0339 | 0.0411 | 0.4782 |
| *Haliscomenobacter* | 0.0482 | 0.0489 | 0.4923 |
| *Pseudopedobacter* | 0.0043 | 0.0277 | 0.4954 |
| *Sphingobacterium* | 0.0357 | 0.0429 | 0.5202 |
| *Arenibacter* | 0.0265 | 0.0407 | 0.5382 |
| *Fermentimonas* | 0.0339 | 0.0411 | 0.5423 |
| *Capnocytophaga* | 0.0157 | 0.0378 | 0.5543 |
| *Rufibacter* | 0.0110 | 0.0365 | 0.5903 |
| *Maribacter* | 0.0299 | 0.0407 | 0.6208 |
| *Petrimonas* | 0.0116 | 0.0365 | 0.6590 |
| *Dialister* | 0.0010 | 0.0198 | 0.0895 |
| *Acutalibacter* | 0.0003 | 0.0192 | 0.1568 |
| *Fastidiosipila* | 0.0395 | 0.0452 | 0.3409 |
| *Faecalibacterium* | 0.0020 | 0.0201 | 0.3590 |
| *Terrisporobacter* | 0.0400 | 0.0452 | 0.3876 |
| *Parvimonas* | 0.0274 | 0.0407 | 0.3999 |
| *Thermincola* | 0.0027 | 0.0222 | 0.4114 |
| *Thermaerobacter* | 0.0339 | 0.0411 | 0.4814 |
| *Coprococcus* | 0.0016 | 0.0201 | 0.5001 |
| *Ruminiclostridium* | 0.0176 | 0.0383 | 0.5482 |
| *Moorella* | 0.0395 | 0.0452 | 0.5518 |
| *Mahella* | 0.0058 | 0.0307 | 0.6014 |
| *Dorea* | 0.0119 | 0.0365 | 0.6245 |
| *Salinicoccus* | 0.0166 | 0.0378 | 0.6359 |
| *Gordonvirus* | 0.0191 | 0.0390 | 0.1281 |
| *Paludisphaera* | 0.0160 | 0.0378 | 0.1587 |
| *Borrelia* | 0.0397 | 0.0452 | 0.2707 |
| *Phycisphaera* | 0.0490 | 0.0491 | 0.2872 |
| *Gemmatirosa* | 0.0013 | 0.0201 | 0.3088 |
| *Hk97virus* | 0.0226 | 0.0404 | 0.3327 |
| *Thermodesulfobacterium* | 0.0312 | 0.0407 | 0.3395 |
| *Oceanithermus* | 0.0385 | 0.0451 | 0.3637 |
| *Methanocorpusculum* | 0.0049 | 0.0279 | 0.3717 |
| *Trichomonas* | 0.0090 | 0.0365 | 0.3973 |
| *Fervidobacterium* | 0.0088 | 0.0365 | 0.4016 |
| *Leishmania* | 0.0265 | 0.0407 | 0.4279 |
| *Prosthecochloris* | 0.0141 | 0.0378 | 0.4483 |
| *Fonsecaea* | 0.0132 | 0.0365 | 0.4630 |
| *Gloeobacter* | 0.0282 | 0.0407 | 0.4903 |
| *Kiritimatiella* | 0.0185 | 0.0385 | 0.5152 |
| *Sphaerospermopsis* | 0.0261 | 0.0407 | 0.5203 |
| *Thermotoga* | 0.0375 | 0.0447 | 0.5326 |
| *Terriglobus* | 0.0466 | 0.0476 | 0.5513 |
| *Luteitalea* | 0.0070 | 0.0355 | 0.5560 |
| *Sneathia* | 0.0185 | 0.0385 | 0.5721 |
| *Thermus* | 0.0166 | 0.0378 | 0.5769 |
| *Pelodictyon* | 0.0299 | 0.0407 | 0.5960 |
| *Roseiflexus* | 0.0169 | 0.0378 | 0.6105 |
| *Geminocystis* | 0.0310 | 0.0407 | 0.6404 |
| *Calothrix* | 0.0148 | 0.0378 | 0.6432 |
| *Leptospira* | 0.0274 | 0.0407 | 0.6453 |
| *Anaerolinea* | 0.0277 | 0.0407 | 0.6516 |
| *Brachyspira* | 0.0116 | 0.0365 | 0.6593 |
| *Caldithrix* | 0.0171 | 0.0378 | 2.8203 |
| *Methylovorus* | 0.0002 | 0.0192 | 0.2332 |
| *Pelagibaca* | 0.0097 | 0.0365 | 0.2432 |
| *Candidatus Symbiobacter* | 0.0079 | 0.0365 | 0.3067 |
| *Sulfurifustis* | 0.0030 | 0.0222 | 0.3566 |
| *Plesiomonas* | 0.0112 | 0.0365 | 0.3805 |
| *Methylocystis* | 0.0206 | 0.0394 | 0.3837 |
| *Kluyvera* | 0.0045 | 0.0279 | 0.3875 |
| *Desulfotalea* | 0.0016 | 0.0201 | 0.3925 |
| *Curvibacter* | 0.0149 | 0.0378 | 0.4007 |
| *Phreatobacter* | 0.0333 | 0.0411 | 0.4093 |
| *Verminephrobacter* | 0.0321 | 0.0411 | 0.4293 |
| *Chromohalobacter* | 0.0200 | 0.0394 | 0.4505 |
| *Alteromonas* | 0.0415 | 0.0453 | 0.4529 |
| *Labrenzia* | 0.0031 | 0.0222 | 0.4627 |
| *Ketobacter* | 0.0111 | 0.0365 | 0.4727 |
| *Acidithiobacillus* | 0.0403 | 0.0452 | 0.4759 |
| *Sterolibacterium* | 0.0118 | 0.0365 | 0.4807 |
| *Ottowia* | 0.0339 | 0.0411 | 0.5128 |
| *Rhodoferax* | 0.0041 | 0.0277 | 0.5170 |
| *Xylella* | 0.0111 | 0.0365 | 0.5201 |
| *Grimontia* | 0.0194 | 0.0392 | 0.5217 |
| *Gallibacterium* | 0.0157 | 0.0378 | 0.5277 |
| *Anaeromyxobacter* | 0.0423 | 0.0453 | 0.5395 |
| *Methylosinus* | 0.0104 | 0.0365 | 0.5433 |
| *Martelella* | 0.0131 | 0.0365 | 0.5486 |
| *Granulosicoccus* | 0.0412 | 0.0453 | 0.5559 |
| *Moraxella* | 0.0444 | 0.0470 | 0.5606 |
| *Basilea* | 0.0328 | 0.0411 | 0.5653 |
| *Novosphingobium* | 0.0256 | 0.0407 | 0.5740 |
| *Salinicola* | 0.0273 | 0.0407 | 0.5787 |
| *Komagataeibacter* | 0.0218 | 0.0403 | 0.5798 |
| *Methyloversatilis* | 0.0412 | 0.0453 | 0.5960 |
| *Syntrophus* | 0.0269 | 0.0407 | 0.5966 |
| *Aminobacter* | 0.0418 | 0.0453 | 0.6057 |
| *Azospira* | 0.0207 | 0.0394 | 0.6278 |
| *Shewanella* | 0.0289 | 0.0407 | 0.6354 |
| *Desulfobacula* | 0.0305 | 0.0407 | 0.6594 |
| *Acidovorax* | 0.0233 | 0.0404 | 0.6612 |
| *Stigmatella* | 0.0007 | 0.0192 | 2.1355 |
| *Salipiger* | 0.0312 | 0.0407 | 2.2233 |
| *Piscirickettsia* | 0.0158 | 0.0378 | 3.0272 |

**Table S5. Comparison of relative abundance of KOs between AMR and control groups**

| **Taxon** | ***P* value** | **Fold change (AMR vs Ctrl)** |
| --- | --- | --- |
| K00009 | 0.037548769 | 1.994297115 |
| K00033 | 0.001498028 | 2.275812596 |
| K00036 | 0.0001656 | 2.270918807 |
| K00042 | 0.014785381 | 0.523948281 |
| K00066 | 0.005730196 | 0.088911436 |
| K00082 | 0.045540202 | 0 |
| K00091 | 0.023616023 | 0.529571701 |
| K00102 | 0.001798162 | 0.381833557 |
| K00126 | 0.008967108 | 0.009214942 |
| K00128 | 0.013938741 | 0.646822777 |
| K00169 | 0.042309964 | 0.589874865 |
| K00179 | 0.007526048 | 0.561243687 |
| K00284 | 0.013938741 | 0.562936669 |
| K00294 | 0.025989147 | 0.607013742 |
| K00302 | 0.005023813 | 0.557996443 |
| K00394 | 0.017600409 | 0.481763323 |
| K00395 | 0.014903478 | 0.36281973 |
| K00455 | 0.011118389 | 5.147642381 |
| K00702 | 0.000286395 | 0.402576624 |
| K00773 | 0.027428263 | 0.640076529 |
| K00803 | 0.019611933 | 0.65317515 |
| K00809 | 0.032617109 | 3.200047201 |
| K00887 | 0.044404223 | 3.270276228 |
| K00899 | 0.01863044 | 1.63674592 |
| K01083 | 0.032319893 | 0.217649216 |
| K01113 | 0.048746387 | 0.551861765 |
| K01114 | 0.022049513 | 0.584806018 |
| K01156 | 0.00968694 | 0.633415762 |
| K01179 | 0.028934139 | 0.492112249 |
| K01181 | 0.017596653 | 0.538213179 |
| K01227 | 0.002684899 | 0.470325927 |
| K01318 | 0.021281352 | 0.103751727 |
| K01387 | 0.015342125 | 0.011477725 |
| K01408 | 0.033224477 | 2.359795416 |
| K01415 | 0.038222483 | 0.161833581 |
| K01421 | 0.013938741 | 0.633819879 |
| K01436 | 0.001678582 | 0.303817349 |
| K01437 | 0.032022504 | 0.546539325 |
| K01446 | 0.015342125 | 0.017609042 |
| K01488 | 0.032155624 | 1.949997852 |
| K01505 | 0.045128133 | 2.791806193 |
| K01533 | 0.002181019 | 0.508411671 |
| K01548 | 0.002713845 | 1.80886007 |
| K01561 | 0.000561518 | 0.322109904 |
| K01585 | 0.006613765 | 0.498303509 |
| K01607 | 0.02330201 | 2.218845524 |
| K01664 | 0.043675737 | 0.527136489 |
| K01707 | 0.02351089 | 0.454075765 |
| K01732 | 0.026599596 | 0 |
| K01755 | 0.000373137 | 0.571994701 |
| K01807 | 0.045893331 | 2.17170329 |
| K01820 | 0.004491618 | 0.332718422 |
| K01836 | 0.016582251 | 2.454548182 |
| K01884 | 0.020430616 | 14.74109172 |
| K01885 | 0.007526048 | 0.599504137 |
| K01897 | 0.013134325 | 0.652547355 |
| K01940 | 0.000863773 | 0.554587922 |
| K01969 | 0.049922318 | 0.584355468 |
| K02001 | 0.037548769 | 2.685003849 |
| K02046 | 0.002713845 | 0.194123893 |
| K02047 | 0.010957418 | 0.438088734 |
| K02048 | 0.001387193 | 0.544686565 |
| K02068 | 0.017596653 | 2.901044841 |
| K02245 | 0.006001764 | 5.196715565 |
| K02280 | 0.041247957 | 0.336811407 |
| K02321 | 0.027900127 | 3.155607151 |
| K02396 | 0.039506106 | 0.517861116 |
| K02397 | 0.014785381 | 0.633248331 |
| K02398 | 0.002882239 | 0.402141114 |
| K02400 | 0.013938741 | 0.505774631 |
| K02404 | 0.008546917 | 0.568056404 |
| K02406 | 0.001187412 | 0.395962719 |
| K02407 | 0.016612488 | 0.638898554 |
| K02412 | 0.033876069 | 0.565568926 |
| K02422 | 0.005076254 | 0.426131817 |
| K02437 | 0.035672944 | 1.763735299 |
| K02476 | 0.025424718 | 2.013862114 |
| K02488 | 0.025228608 | 0.151977735 |
| K02532 | 0.035672944 | 2.008608327 |
| K02557 | 0.007056965 | 0.642976971 |
| K02604 | 0.027900127 | 4.688461577 |
| K02655 | 0.006248835 | 0.325036927 |
| K02659 | 0.018129608 | 0.445108406 |
| K02660 | 0.007835519 | 0.511725923 |
| K02662 | 0.00252456 | 0.49552388 |
| K02664 | 0.020049083 | 0.428686277 |
| K02670 | 0.005541115 | 0.453409984 |
| K02673 | 0.004094322 | 0.25249129 |
| K02685 | 0.033224477 | 2.153278552 |
| K02725 | 0.049789843 | 5.4129088 |
| K02759 | 0.000734009 | 2.34146778 |
| K02760 | 0.000796499 | 2.226431234 |
| K02761 | 0.004745183 | 2.273341114 |
| K02773 | 0.011645316 | 1.909316497 |
| K02781 | 0.00413986 | 5.607540498 |
| K02793 | 0.004745183 | 2.820439656 |
| K02794 | 0.000863773 | 2.686051305 |
| K02795 | 0.016612488 | 1.876450243 |
| K02796 | 0.008022266 | 2.144061852 |
| K02818 | 0.043675737 | 3.264052861 |
| K02822 | 0.025989147 | 2.523038361 |
| K02886 | 0.003360054 | 0.628338766 |
| K02937 | 0.040717224 | 3.198979272 |
| K02999 | 0.033224477 | 1.81271723 |
| K03018 | 0.025092466 | 5.04888118 |
| K03037 | 0.023913504 | 7.942013538 |
| K03040 | 0.043675737 | 0.661218866 |
| K03061 | 0.034949289 | 1.539143329 |
| K03078 | 0.005443556 | 0.356693361 |
| K03090 | 0.001625811 | 0.297271392 |
| K03147 | 0.003863721 | 0.616827827 |
| K03178 | 0.031004562 | 1.993395831 |
| K03205 | 0.002713845 | 0.627623637 |
| K03239 | 0.010550427 | 4.624538496 |
| K03242 | 0.041354664 | 5.52385772 |
| K03322 | 0.04154755 | 2.404570563 |
| K03407 | 0.012370409 | 0.615307777 |
| K03415 | 0.002713845 | 0.45648598 |
| K03430 | 0.039506106 | 1.896706814 |
| K03475 | 0.02330201 | 1.861850916 |
| K03481 | 0.032155624 | 2.839457744 |
| K03492 | 0.000796499 | 6.120967002 |
| K03503 | 0.045893331 | 1.856822388 |
| K03618 | 0.024518525 | 0.250699828 |
| K03719 | 0.001097603 | 0.450778553 |
| K03779 | 0.003741021 | 0.44264738 |
| K03800 | 0.037548769 | 1.89103629 |
| K03827 | 0.010957418 | 1.584284073 |
| K03830 | 0.039506106 | 1.694889493 |
| K03885 | 0.01863044 | 1.698554944 |
| K03929 | 0.012370409 | 0.542444682 |
| K03933 | 0.010718362 | 11.48915001 |
| K04043 | 0.002713845 | 0.658318916 |
| K04109 | 0.008769332 | 0 |
| K04562 | 0.02330201 | 0.44272987 |
| K04564 | 0.048203031 | 1.606812022 |
| K04749 | 0.01759175 | 2.77639022 |
| K04767 | 0.045893331 | 2.783864387 |
| K05305 | 0.00313086 | 0.546264153 |
| K05343 | 0.004745183 | 0.41668462 |
| K05559 | 0.039325583 | 0.628270342 |
| K05791 | 0.036199744 | 4.715804637 |
| K05873 | 0.036507526 | 0.293171504 |
| K05910 | 0.010476343 | 37.15568168 |
| K05995 | 0.017596653 | 0.626464811 |
| K06012 | 0.002915706 | 0.602822687 |
| K06156 | 0.008627722 | 0.239125105 |
| K06200 | 0.004745183 | 0.623513168 |
| K06215 | 0.033876069 | 0.661169648 |
| K06237 | 0.027212404 | 0.020602887 |
| K06298 | 0.00360407 | 0.633107783 |
| K06330 | 0.033049928 | 0.564730845 |
| K06342 | 0.027900127 | 0.036525944 |
| K06406 | 0.015676022 | 0.651654385 |
| K06446 | 0.022313659 | 0.621160609 |
| K06603 | 0.014098914 | 0.310893802 |
| K06880 | 0.031340413 | 0.315804912 |
| K06883 | 0.00180994 | 4.684802433 |
| K06886 | 0.044564659 | 2.023710844 |
| K06902 | 0.010957418 | 0.623031879 |
| K06952 | 0.008546917 | 0.542468446 |
| K06970 | 0.018061243 | 2.843680612 |
| K06974 | 0.04912159 | 0.09752736 |
| K07078 | 0.013938741 | 2.418081734 |
| K07099 | 0.019715823 | 0.656045642 |
| K07106 | 0.00252456 | 2.161360309 |
| K07118 | 0.032155624 | 1.982411733 |
| K07126 | 0.004433372 | 0.628156149 |
| K07160 | 0.009101346 | 0.639592028 |
| K07223 | 0.011645316 | 2.088536354 |
| K07301 | 0.005076254 | 0.643625333 |
| K07302 | 0.024518525 | 0.472967975 |
| K07339 | 0.033671672 | 0.247941797 |
| K07341 | 0.027428263 | 0.538453726 |
| K07374 | 0.046244662 | 2.823693123 |
| K07399 | 0.006195239 | 0.496183119 |
| K07404 | 0.006195239 | 1.87528285 |
| K07469 | 0.0020443 | 0.511922892 |
| K07481 | 0.022049513 | 0.548509748 |
| K07488 | 0.034412596 | 0.638008621 |
| K07559 | 0.035672944 | 2.170299503 |
| K07649 | 0.042262626 | 0.564358515 |
| K07704 | 0.010305137 | 0.493854376 |
| K07739 | 0.034403204 | 0.429368658 |
| K07741 | 0.04154755 | 0.372392566 |
| K07775 | 0.006185304 | 0.585642583 |
| K07777 | 0.009960308 | 0.323845009 |
| K07813 | 0.007056965 | 0.463822558 |
| K07814 | 0.006195239 | 0.612690291 |
| K07862 | 0.006195239 | 0.597713901 |
| K07874 | 0.046244662 | 3.649376149 |
| K07936 | 0.015342125 | 3.473830635 |
| K08002 | 0.043643259 | #DIV/0! |
| K08084 | 0.029864461 | 0.336085026 |
| K08217 | 0.024614483 | 1.839508069 |
| K08258 | 0.001113388 | 0.205767213 |
| K08344 | 0.004846827 | 1.635081312 |
| K08372 | 0.016612488 | 0.655365349 |
| K08478 | 0.042529661 | 0.374286953 |
| K08693 | 0.005150944 | 0.460229431 |
| K08714 | 0.034115298 | 9.774334951 |
| K08737 | 0.046118846 | 3.269518691 |
| K08981 | 0.013134325 | 0.558231796 |
| K09004 | 0.004433372 | 6.366462656 |
| K09118 | 0.019715823 | 0.557128414 |
| K09165 | 0.039425553 | 0.299947679 |
| K09419 | 0.015342125 | 1.883490647 |
| K09461 | 0.012466778 | 0.546380799 |
| K09495 | 0.035579029 | 3.328282321 |
| K09760 | 0.017596653 | 0.565577762 |
| K09766 | 0.015309028 | 0.301275713 |
| K09768 | 0.006613765 | 0.549647111 |
| K09799 | 0.002340032 | 0.61651823 |
| K09803 | 0.003863721 | 0.299706563 |
| K09935 | 0.017596653 | 0.517806672 |
| K09963 | 0.009101346 | 4.227844075 |
| K09974 | 0.025571047 | 0.484192594 |
| K10005 | 0.013003689 | 0.172728424 |
| K10006 | 0.011783141 | 0.201734841 |
| K10007 | 0.003272854 | 0.185669756 |
| K10008 | 0.00744402 | 0.308921369 |
| K10195 | 0.040036316 | 1.639625575 |
| K10353 | 0.005111259 | 105.9089747 |
| K10536 | 0.007056965 | 0.501651619 |
| K10563 | 0.001743916 | 2.111843927 |
| K10798 | 0.01716964 | 0.214590913 |
| K10901 | 0.013491202 | 3.495311631 |
| K11254 | 0.022112481 | 6.483675341 |
| K11263 | 0.023032134 | 0.367709568 |
| K11493 | 0.031004562 | 3.19822499 |
| K11535 | 0.040203038 | 11.63645481 |
| K11672 | 0.039789284 | 5.251859818 |
| K11694 | 0.009792451 | 0.123102347 |
| K11711 | 0.003288402 | 0.394303273 |
| K11712 | 0.036024305 | 0.178731895 |
| K11838 | 0.0130802 | 3.457220343 |
| K11897 | 0.0081505 | 0.241090807 |
| K12240 | 0.039425553 | 0.149310176 |
| K12255 | 0.035365878 | 4.397800489 |
| K12506 | 0.010305137 | 0.611221913 |
| K12528 | 0.02655988 | 0.475623077 |
| K12532 | 0.03154774 | 14.56988987 |
| K12537 | 0.04616635 | 10.08414907 |
| K12561 | 0.040575452 | 0.074467429 |
| K12585 | 0.028913185 | 3.161028532 |
| K12603 | 0.031921553 | 3.573741134 |
| K12613 | 0.027900127 | 3.086214072 |
| K12818 | 0.035579029 | 2.636838856 |
| K12875 | 0.045540202 | 0 |
| K12982 | 0.021281352 | 0.126461409 |
| K12990 | 0.012590729 | 0.326046533 |
| K12991 | 0.045540202 | 0 |
| K13021 | 0.004207221 | 42.12289099 |
| K13049 | 0.002360785 | 0.61748673 |
| K13126 | 0.011118389 | 3.362830608 |
| K13282 | 0.013604184 | 0.51958982 |
| K13412 | 0.038074475 | 0.042228636 |
| K13531 | 0.01023921 | 21.36297506 |
| K13607 | 0.027676319 | 0.383358643 |
| K13688 | 0.032656262 | 0.336955763 |
| K13694 | 0.007526048 | 3.183058898 |
| K13874 | 0.047110394 | 0.332752553 |
| K13931 | 0.036146492 | 3.932680575 |
| K13989 | 0.018421803 | 5.514676702 |
| K14086 | 0.008104951 | 0.227394731 |
| K14138 | 0.005076254 | 0.634835293 |
| K14161 | 0.047516285 | 0.492265123 |
| K14194 | 0.010889215 | 0.611007852 |
| K14437 | 0.043643259 | #DIV/0! |
| K14560 | 0.041354664 | 2.980201182 |
| K14681 | 0.045540202 | 0 |
| K14731 | 0.031409603 | 0.619672012 |
| K14753 | 0.046244662 | 4.907348785 |
| K14835 | 0.030099868 | 2.127504375 |
| K15023 | 0.008854251 | 0.548928232 |
| K15373 | 0.020405827 | 3.279673052 |
| K15534 | 0.029864191 | 0.259323469 |
| K15667 | 0.008703036 | 0.337470952 |
| K15739 | 0.040813464 | 0.361625247 |
| K15984 | 0.045893331 | 0.450087768 |
| K16139 | 0.019611933 | 0.469458179 |
| K16147 | 0.04154755 | 0.589855219 |
| K16196 | 0.038698613 | 3.456534345 |
| K16435 | 0.017244178 | 21.67816843 |
| K16509 | 0.008627722 | 8.779302445 |
| K16568 | 0.011208202 | 0.108815517 |
| K16850 | 0.028373229 | 0.576336001 |
| K16881 | 0.010777436 | 0.266981791 |
| K16917 | 0.04798975 | 0.283946631 |
| K17301 | 0.017832758 | 3.122942807 |
| K17315 | 0.005150944 | 0.250358057 |
| K17316 | 0.029302634 | 0.163219508 |
| K17317 | 0.031415321 | 0.20165196 |
| K17329 | 0.011496297 | 0.585029788 |
| K17363 | 0.048855826 | 0.404017343 |
| K17462 | 0.016228947 | 0.507992897 |
| K17615 | 0.027900127 | 4.545947914 |
| K17623 | 0.002275498 | 0.063568983 |
| K17818 | 0.04536397 | 0.606602918 |
| K17922 | 0.025840401 | 3.441510382 |
| K18011 | 0.045670056 | 0.419877426 |
| K18013 | 0.040299876 | 0.540594643 |
| K18136 | 0.04651644 | 0.363054258 |
| K18231 | 0.003360054 | 3.889038804 |
| K18236 | 0.042914462 | 17.38890458 |
| K18237 | 0.038277561 | 4.874605266 |
| K18346 | 0.042309964 | 1.578901506 |
| K18480 | 0.021465629 | 0.390674115 |
| K18555 | 0.006489244 | 113.9677658 |
| K18581 | 0.022049513 | 2.25299955 |
| K18590 | 0.049789843 | 4.303332795 |
| K18591 | 0.014025022 | 144.7586622 |
| K18815 | 0.034542546 | 4.158944044 |
| K18843 | 0.016663098 | 0.319647524 |
| K18903 | 0.017129604 | 0.516552409 |
| K18907 | 0.042529661 | 0.23296469 |
| K19123 | 0.00252456 | 0.330231066 |
| K19140 | 0.032155624 | 0.466337928 |
| K19165 | 0.01450002 | 0.418022487 |
| K19167 | 0.045893331 | 0.598743645 |
| K19231 | 0.039225218 | 0.317001944 |
| K19309 | 0.007526048 | 0.352446543 |
| K19310 | 0.008546917 | 0.328323528 |
| K19349 | 0.003498461 | 0.297248866 |
| K19419 | 0.00614465 | 0.195392935 |
| K19430 | 0.009719512 | 0.033120307 |
| K19519 | 0.045540202 | 0 |
| K19545 | 0.00580022 | 0.530421539 |
| K19837 | 0.035365878 | 5.064607471 |
| K19975 | 0.036020126 | 13.65432883 |
| K20165 | 0.035838727 | 0.364894777 |
| K20265 | 0.008546917 | 4.743449867 |
| K20344 | 0.005427586 | 0.493826731 |
| K20345 | 0.003986493 | 0.433380105 |
| K20391 | 0.011818136 | 0.580562895 |
| K20483 | 0.009960308 | 0.458513113 |
| K20485 | 0.007859244 | 0.365281728 |

**Table S6.** **Interrelationship between AMR-associated gut microbial species, functions and metabolites**

| **Species** | **Fuction** | **Metabolite** | **Species-function correlation** | **Function-metabolite correlation** | **Species-metabolite correlation** |
| --- | --- | --- | --- | --- | --- |
| *Acutalibacter muris* | K00033 | 3b-Hydroxy-5-cholenoic acid | -0.500592448 | -0.587596899 | 0.500476983 |
| *Bifidobacterium choerinum* | K00033 | 3b-Hydroxy-5-cholenoic acid | -0.501173592 | -0.587596899 | 0.571186924 |
| *Pantoea sp. PSNIH1* | K00033 | 3b-Hydroxy-5-cholenoic acid | -0.502234338 | -0.587596899 | 0.602821024 |
| *Microbacterium sp. XT11* | K00033 | 3b-Hydroxy-5-cholenoic acid | -0.503944932 | -0.587596899 | 0.511294374 |
| *Aeromonas sp. CU5* | K00033 | 3b-Hydroxy-5-cholenoic acid | -0.504403606 | -0.587596899 | 0.549479379 |
| *endosymbiont of unidentified scaly snail isolate Monju* | K00033 | 3b-Hydroxy-5-cholenoic acid | -0.513284809 | -0.587596899 | 0.535094959 |
| *Pseudomonas putida* | K00033 | 3b-Hydroxy-5-cholenoic acid | -0.523265306 | -0.587596899 | 0.60521494 |
| *Luteitalea pratensis* | K00033 | 3b-Hydroxy-5-cholenoic acid | -0.521696982 | -0.587596899 | 0.599034498 |
| *Thermoplasmatales archaeon BRNA1* | K00033 | 3b-Hydroxy-5-cholenoic acid | -0.524379824 | -0.587596899 | 0.544471476 |
| *Synechococcus sp. SynAce01* | K00033 | 3b-Hydroxy-5-cholenoic acid | -0.530106741 | -0.587596899 | 0.554008598 |
| *[Eubacterium] rectale* | K00033 | 3b-Hydroxy-5-cholenoic acid | -0.543979592 | -0.587596899 | 0.541649049 |
| *Methanocorpusculum labreanum* | K00033 | 3b-Hydroxy-5-cholenoic acid | -0.542694778 | -0.587596899 | 0.509049458 |
| *Labrenzia sp. VG12* | K00033 | 3b-Hydroxy-5-cholenoic acid | -0.628825907 | -0.587596899 | 0.553757475 |
| *[Eubacterium] rectale* | K00036 | 3b-Hydroxy-5-cholenoic acid | -0.512653061 | -0.572797745 | 0.541649049 |
| *endosymbiont of unidentified scaly snail isolate Monju* | K00036 | 3b-Hydroxy-5-cholenoic acid | -0.512773874 | -0.572797745 | 0.535094959 |
| *Gordonibacter urolithinfaciens* | K00036 | 3b-Hydroxy-5-cholenoic acid | -0.522142857 | -0.572797745 | 0.566173362 |
| *Microbacterium sp. XT11* | K00036 | 3b-Hydroxy-5-cholenoic acid | -0.531098695 | -0.572797745 | 0.511294374 |
| *Thermoplasmatales archaeon BRNA1* | K00036 | 3b-Hydroxy-5-cholenoic acid | -0.537104501 | -0.572797745 | 0.544471476 |
| *Gordonibacter pamelaeae* | K00036 | 3b-Hydroxy-5-cholenoic acid | -0.589489796 | -0.572797745 | 0.650599013 |
| *Methanocorpusculum labreanum* | K00036 | 3b-Hydroxy-5-cholenoic acid | -0.612180571 | -0.572797745 | 0.509049458 |
| *Lachnoclostridium phocaeense* | K00179 | 3b-Hydroxy-5-cholenoic acid | 0.50255102 | 0.500916138 | 0.519520789 |
| *Thermincola potens* | K00179 | 3b-Hydroxy-5-cholenoic acid | 0.508469388 | 0.500916138 | 0.571247357 |
| *Slackia heliotrinireducens* | K00179 | 3b-Hydroxy-5-cholenoic acid | 0.509489796 | 0.500916138 | 0.599295278 |
| *Moorella thermoacetica* | K00179 | 3b-Hydroxy-5-cholenoic acid | 0.512653061 | 0.500916138 | 0.518111346 |
| *Acutalibacter muris* | K00179 | 3b-Hydroxy-5-cholenoic acid | 0.516469251 | 0.500916138 | 0.500476983 |
| *Cryobacterium arcticum* | K00179 | 3b-Hydroxy-5-cholenoic acid | 0.518836208 | 0.500916138 | 0.526798438 |
| *Cutibacterium acnes* | K00179 | 3b-Hydroxy-5-cholenoic acid | 0.524042947 | 0.500916138 | 0.504654568 |
| *Geobacter uraniireducens* | K00179 | 3b-Hydroxy-5-cholenoic acid | 0.528841314 | 0.500916138 | 0.53173497 |
| *Rhodoferax ferrireducens* | K00179 | 3b-Hydroxy-5-cholenoic acid | 0.529658058 | 0.500916138 | 0.525529045 |
| *Pseudomonas putida* | K00179 | 3b-Hydroxy-5-cholenoic acid | 0.538265306 | 0.500916138 | 0.60521494 |
| *Bradyrhizobium sp. BTAi1* | K00179 | 3b-Hydroxy-5-cholenoic acid | 0.531630214 | 0.500916138 | 0.52498704 |
| *Plantactinospora sp. KBS50* | K00179 | 3b-Hydroxy-5-cholenoic acid | 0.532256138 | 0.500916138 | 0.519691721 |
| *Geobacter metallireducens* | K00179 | 3b-Hydroxy-5-cholenoic acid | 0.534291158 | 0.500916138 | 0.509685593 |
| *Thermoplasmatales archaeon BRNA1* | K00179 | 3b-Hydroxy-5-cholenoic acid | 0.534436424 | 0.500916138 | 0.544471476 |
| *Ruminococcus champanellensis* | K00179 | 3b-Hydroxy-5-cholenoic acid | 0.542755102 | 0.500916138 | 0.597744891 |
| *Phreatobacter cathodiphilus* | K00179 | 3b-Hydroxy-5-cholenoic acid | 0.539128664 | 0.500916138 | 0.548613563 |
| *Desulfovibrio piger* | K00179 | 3b-Hydroxy-5-cholenoic acid | 0.546734694 | 0.500916138 | 0.517688513 |
| *Xanthomonas oryzae* | K00179 | 3b-Hydroxy-5-cholenoic acid | 0.539410403 | 0.500916138 | 0.528418113 |
| *Gordonibacter urolithinfaciens* | K00179 | 3b-Hydroxy-5-cholenoic acid | 0.547653061 | 0.500916138 | 0.566173362 |
| *Bifidobacterium scardovii* | K00179 | 3b-Hydroxy-5-cholenoic acid | 0.562244898 | 0.500916138 | 0.593375617 |
| *Gemmatirosa kalamazoonesis* | K00179 | 3b-Hydroxy-5-cholenoic acid | 0.570774921 | 0.500916138 | 0.544803213 |
| *Roseiflexus castenholzii* | K00179 | 3b-Hydroxy-5-cholenoic acid | 0.577946609 | 0.500916138 | 0.537800504 |
| *butyrate-producing bacterium SM4/1* | K00179 | 3b-Hydroxy-5-cholenoic acid | 0.590918367 | 0.500916138 | 0.502184637 |
| *Desulfovibrio salexigens* | K00179 | 3b-Hydroxy-5-cholenoic acid | 0.582305863 | 0.500916138 | 0.520727157 |
| *Thermaerobacter marianensis* | K00179 | 3b-Hydroxy-5-cholenoic acid | 0.593265306 | 0.500916138 | 0.6136716 |
| *Rhodothermus marinus* | K00179 | 3b-Hydroxy-5-cholenoic acid | 0.585365857 | 0.500916138 | 0.552309807 |
| *Clostridium sp. enrichment culture clone 7-14* | K00179 | 3b-Hydroxy-5-cholenoic acid | 0.588041631 | 0.500916138 | 0.611670924 |
| *Acidovorax carolinensis* | K00179 | 3b-Hydroxy-5-cholenoic acid | 0.590381494 | 0.500916138 | 0.688187379 |
| *Mordavella sp. Marseille-P3756* | K00179 | 3b-Hydroxy-5-cholenoic acid | 0.607244898 | 0.500916138 | 0.5863284 |
| *Gordonibacter pamelaeae* | K00179 | 3b-Hydroxy-5-cholenoic acid | 0.611938776 | 0.500916138 | 0.650599013 |
| *Oxalobacter formigenes* | K00179 | 3b-Hydroxy-5-cholenoic acid | 0.623163265 | 0.500916138 | 0.652290345 |
| *Pseudomonas citronellolis* | K00179 | 3b-Hydroxy-5-cholenoic acid | 0.623877551 | 0.500916138 | 0.599295278 |
| *Serratia marcescens* | K00179 | 3b-Hydroxy-5-cholenoic acid | 0.626938776 | 0.500916138 | 0.596899225 |
| *Gloeobacter violaceus* | K00179 | 3b-Hydroxy-5-cholenoic acid | 0.616068012 | 0.500916138 | 0.597210283 |
| *Chromohalobacter salexigens* | K00179 | 3b-Hydroxy-5-cholenoic acid | 0.625151055 | 0.500916138 | 0.609827901 |
| *Paenibacillus mucilaginosus* | K00179 | 3b-Hydroxy-5-cholenoic acid | 0.647142857 | 0.500916138 | 0.576603242 |
| *Pectobacterium polaris* | K00179 | 3b-Hydroxy-5-cholenoic acid | 0.6375593 | 0.500916138 | 0.522138341 |
| *Deinococcus proteolyticus* | K00179 | 3b-Hydroxy-5-cholenoic acid | 0.648793825 | 0.500916138 | 0.561792631 |
| *Eggerthella sp. YY7918* | K00179 | 3b-Hydroxy-5-cholenoic acid | 0.675714286 | 0.500916138 | 0.657505285 |
| *Acidovorax avenae* | K00179 | 3b-Hydroxy-5-cholenoic acid | 0.698163265 | 0.500916138 | 0.612684989 |
| *Azospira oryzae* | K00179 | 3b-Hydroxy-5-cholenoic acid | 0.727319118 | 0.500916138 | 0.601430636 |
| *Acidovorax carolinensis* | K01897 | L-Pipecolic acid | 0.542684237 | 0.539393939 | 0.539707626 |
| *Luteitalea pratensis* | K02245 | 3b-Hydroxy-5-cholenoic acid | -0.519618348 | -0.512755462 | 0.599034498 |
| *Verminephrobacter eiseniae* | K02532 | Taurocholate | -0.507034544 | 0.558985201 | -0.527080526 |
| *Paenibacillus mucilaginosus* | K02532 | Taurocholate | -0.606530612 | 0.558985201 | -0.540803383 |
| *Oxalobacter formigenes* | K02532 | Taurocholate | -0.625204082 | 0.558985201 | -0.501057082 |
| *Ruminococcus champanellensis* | K02532 | Taurocholate | -0.633061224 | 0.558985201 | -0.548132488 |
| *Serratia marcescens* | K03929 | 3b-Hydroxy-5-cholenoic acid | 0.507959184 | 0.550669486 | 0.596899225 |
| *Bifidobacterium scardovii* | K03929 | 3b-Hydroxy-5-cholenoic acid | 0.508673469 | 0.550669486 | 0.593375617 |
| *Pseudomonas putida* | K03929 | 3b-Hydroxy-5-cholenoic acid | 0.50877551 | 0.550669486 | 0.60521494 |
| *Acidovorax carolinensis* | K03929 | 3b-Hydroxy-5-cholenoic acid | 0.507883664 | 0.550669486 | 0.688187379 |
| *Thermincola potens* | K03929 | 3b-Hydroxy-5-cholenoic acid | 0.514183673 | 0.550669486 | 0.571247357 |
| *Comamonas kerstersii* | K03929 | 3b-Hydroxy-5-cholenoic acid | 0.515554645 | 0.550669486 | 0.635275793 |
| *Microbacterium sp. XT11* | K03929 | 3b-Hydroxy-5-cholenoic acid | 0.524077951 | 0.550669486 | 0.511294374 |
| *Chromohalobacter salexigens* | K03929 | 3b-Hydroxy-5-cholenoic acid | 0.528074085 | 0.550669486 | 0.609827901 |
| *Ketobacter alkanivorans* | K03929 | 3b-Hydroxy-5-cholenoic acid | 0.529119956 | 0.550669486 | 0.506760393 |
| *Slackia heliotrinireducens* | K03929 | 3b-Hydroxy-5-cholenoic acid | 0.585408163 | 0.550669486 | 0.599295278 |
| *Lachnoclostridium phocaeense* | K07223 | 3b-Hydroxy-5-cholenoic acid | -0.508673469 | -0.509654686 | 0.519520789 |
| *Paenibacillus mucilaginosus* | K07223 | 3b-Hydroxy-5-cholenoic acid | -0.509285714 | -0.509654686 | 0.576603242 |
| *Rhodoferax ferrireducens* | K07223 | 3b-Hydroxy-5-cholenoic acid | -0.538948513 | -0.509654686 | 0.525529045 |
| *Paenibacillus mucilaginosus* | K07469 | 3b-Hydroxy-5-cholenoic acid | 0.514949778 | 0.511625015 | 0.576603242 |
| *Chromohalobacter salexigens* | K07469 | 3b-Hydroxy-5-cholenoic acid | 0.525105424 | 0.511625015 | 0.609827901 |
| *Moorella thermoacetica* | K07469 | 3b-Hydroxy-5-cholenoic acid | 0.531801566 | 0.511625015 | 0.518111346 |
| *Gloeobacter violaceus* | K07469 | 3b-Hydroxy-5-cholenoic acid | 0.536695289 | 0.511625015 | 0.597210283 |
| *Synechococcus sp. SynAce01* | K07469 | 3b-Hydroxy-5-cholenoic acid | 0.536761191 | 0.511625015 | 0.554008598 |
| *Acidovorax avenae* | K07469 | 3b-Hydroxy-5-cholenoic acid | 0.538235885 | 0.511625015 | 0.612684989 |
| *Thermincola potens* | K07469 | 3b-Hydroxy-5-cholenoic acid | 0.543087157 | 0.511625015 | 0.571247357 |
| *Azotobacter chroococcum* | K07469 | 3b-Hydroxy-5-cholenoic acid | 0.544465939 | 0.511625015 | 0.519802678 |
| *Labrenzia sp. VG12* | K07469 | 3b-Hydroxy-5-cholenoic acid | 0.545413286 | 0.511625015 | 0.553757475 |
| *Thermoplasmatales archaeon BRNA1* | K07469 | 3b-Hydroxy-5-cholenoic acid | 0.545904221 | 0.511625015 | 0.544471476 |
| *Thermus thermophilus* | K07469 | 3b-Hydroxy-5-cholenoic acid | 0.546394854 | 0.511625015 | 0.614186547 |
| *Streptomyces gilvosporeus* | K07469 | 3b-Hydroxy-5-cholenoic acid | 0.549876002 | 0.511625015 | 0.521609083 |
| *Bradyrhizobium sp. BTAi1* | K07469 | 3b-Hydroxy-5-cholenoic acid | 0.550515879 | 0.511625015 | 0.52498704 |
| *Pantoea gaviniae* | K07469 | 3b-Hydroxy-5-cholenoic acid | 0.550772291 | 0.511625015 | 0.542132254 |
| *Pectobacterium polaris* | K07469 | 3b-Hydroxy-5-cholenoic acid | 0.55387171 | 0.511625015 | 0.522138341 |
| *Pelodictyon luteolum* | K07469 | 3b-Hydroxy-5-cholenoic acid | 0.555013452 | 0.511625015 | 0.502714166 |
| *Pseudomonas citronellolis* | K07469 | 3b-Hydroxy-5-cholenoic acid | 0.556466455 | 0.511625015 | 0.599295278 |
| *Kiritimatiella glycovorans* | K07469 | 3b-Hydroxy-5-cholenoic acid | 0.559509557 | 0.511625015 | 0.519280963 |
| *Acidovorax carolinensis* | K07469 | 3b-Hydroxy-5-cholenoic acid | 0.570829884 | 0.511625015 | 0.688187379 |
| *Mucilaginibacter mallensis* | K07469 | 3b-Hydroxy-5-cholenoic acid | 0.586770393 | 0.511625015 | 0.522985052 |
| *Roseiflexus castenholzii* | K07469 | 3b-Hydroxy-5-cholenoic acid | 0.61225495 | 0.511625015 | 0.537800504 |
| *Desulfovibrio salexigens* | K07469 | 3b-Hydroxy-5-cholenoic acid | 0.622744697 | 0.511625015 | 0.520727157 |
| *Comamonas kerstersii* | K07469 | 3b-Hydroxy-5-cholenoic acid | 0.627250626 | 0.511625015 | 0.635275793 |
| *Phreatobacter cathodiphilus* | K07469 | 3b-Hydroxy-5-cholenoic acid | 0.629216608 | 0.511625015 | 0.548613563 |
| *Luteitalea pratensis* | K07469 | 3b-Hydroxy-5-cholenoic acid | 0.636758487 | 0.511625015 | 0.599034498 |
| *Xanthomonas oryzae* | K07469 | 3b-Hydroxy-5-cholenoic acid | 0.637413749 | 0.511625015 | 0.528418113 |
| *Azospira oryzae* | K07469 | 3b-Hydroxy-5-cholenoic acid | 0.640535427 | 0.511625015 | 0.601430636 |
| *Gemmatirosa kalamazoonesis* | K07469 | 3b-Hydroxy-5-cholenoic acid | 0.641183049 | 0.511625015 | 0.544803213 |
| *Oxalobacter formigenes* | K07469 | 3b-Hydroxy-5-cholenoic acid | 0.658496369 | 0.511625015 | 0.652290345 |
| *Desulfovibrio piger* | K07469 | 3b-Hydroxy-5-cholenoic acid | 0.759453897 | 0.511625015 | 0.517688513 |
| *Candidatus Saccharibacteria oral taxon TM7x* | K09799 | 6k-PGF1alpha-d4 | 0.622831233 | 0.562920857 | 0.57688513 |
| *Cryobacterium arcticum* | K13049 | 3b-Hydroxy-5-cholenoic acid | 0.500574346 | 0.606293386 | 0.526798438 |
| *Actinomyces succiniciruminis* | K13049 | 3b-Hydroxy-5-cholenoic acid | 0.504655731 | 0.606293386 | 0.550669486 |
| *Gemmatirosa kalamazoonesis* | K13049 | 3b-Hydroxy-5-cholenoic acid | 0.504692675 | 0.606293386 | 0.544803213 |
| *Phreatobacter cathodiphilus* | K13049 | 3b-Hydroxy-5-cholenoic acid | 0.51431964 | 0.606293386 | 0.548613563 |
| *Thermus thermophilus* | K13049 | 3b-Hydroxy-5-cholenoic acid | 0.514822185 | 0.606293386 | 0.614186547 |
| *[Eubacterium] rectale* | K13049 | 3b-Hydroxy-5-cholenoic acid | 0.515880507 | 0.606293386 | 0.541649049 |
| *Xanthomonas oryzae* | K13049 | 3b-Hydroxy-5-cholenoic acid | 0.522418745 | 0.606293386 | 0.528418113 |
| *Rhodothermus marinus* | K13049 | 3b-Hydroxy-5-cholenoic acid | 0.527005997 | 0.606293386 | 0.552309807 |
| *Serratia marcescens* | K13049 | 3b-Hydroxy-5-cholenoic acid | 0.531391107 | 0.606293386 | 0.596899225 |
| *Gloeobacter violaceus* | K13049 | 3b-Hydroxy-5-cholenoic acid | 0.532463582 | 0.606293386 | 0.597210283 |
| *Thermoplasmatales archaeon BRNA1* | K13049 | 3b-Hydroxy-5-cholenoic acid | 0.534142195 | 0.606293386 | 0.544471476 |
| *Pseudomonas putida* | K13049 | 3b-Hydroxy-5-cholenoic acid | 0.545524121 | 0.606293386 | 0.60521494 |
| *Desulfovibrio salexigens* | K13049 | 3b-Hydroxy-5-cholenoic acid | 0.55334001 | 0.606293386 | 0.520727157 |
| *Clostridium sp. enrichment culture clone 7-14* | K13049 | 3b-Hydroxy-5-cholenoic acid | 0.553727283 | 0.606293386 | 0.611670924 |
| *Acutalibacter muris* | K13049 | 3b-Hydroxy-5-cholenoic acid | 0.555807432 | 0.606293386 | 0.500476983 |
| *Plantactinospora sp. KBS50* | K13049 | 3b-Hydroxy-5-cholenoic acid | 0.563948641 | 0.606293386 | 0.519691721 |
| *Acidovorax carolinensis* | K13049 | 3b-Hydroxy-5-cholenoic acid | 0.566342604 | 0.606293386 | 0.688187379 |
| *Bifidobacterium choerinum* | K13049 | 3b-Hydroxy-5-cholenoic acid | 0.569766554 | 0.606293386 | 0.571186924 |
| *Stigmatella aurantiaca* | K13049 | 3b-Hydroxy-5-cholenoic acid | 0.570013081 | 0.606293386 | 0.531876014 |
| *Eggerthella sp. YY7918* | K13049 | 3b-Hydroxy-5-cholenoic acid | 0.57623919 | 0.606293386 | 0.657505285 |
| *Deinococcus proteolyticus* | K13049 | 3b-Hydroxy-5-cholenoic acid | 0.589265874 | 0.606293386 | 0.561792631 |
| *Hymenobacter sedentarius* | K13049 | 3b-Hydroxy-5-cholenoic acid | 0.599887739 | 0.606293386 | 0.670849531 |
| *Rhodoferax ferrireducens* | K13049 | 3b-Hydroxy-5-cholenoic acid | 0.61379828 | 0.606293386 | 0.525529045 |
| *Olsenella umbonata* | K13049 | 3b-Hydroxy-5-cholenoic acid | 0.618383122 | 0.606293386 | 0.594362227 |
| *Moorella thermoacetica* | K13049 | 3b-Hydroxy-5-cholenoic acid | 0.631495702 | 0.606293386 | 0.518111346 |
| *Thermaerobacter marianensis* | K13049 | 3b-Hydroxy-5-cholenoic acid | 0.640016327 | 0.606293386 | 0.6136716 |
| *Paenibacillus mucilaginosus* | K13049 | 3b-Hydroxy-5-cholenoic acid | 0.640781653 | 0.606293386 | 0.576603242 |
| *Gordonibacter pamelaeae* | K13049 | 3b-Hydroxy-5-cholenoic acid | 0.65323095 | 0.606293386 | 0.650599013 |
| *Gordonibacter urolithinfaciens* | K13049 | 3b-Hydroxy-5-cholenoic acid | 0.654353428 | 0.606293386 | 0.566173362 |
| *Ruminococcus champanellensis* | K13049 | 3b-Hydroxy-5-cholenoic acid | 0.667312942 | 0.606293386 | 0.597744891 |
| *Bifidobacterium scardovii* | K13049 | 3b-Hydroxy-5-cholenoic acid | 0.672415113 | 0.606293386 | 0.593375617 |
| *Slackia heliotrinireducens* | K13049 | 3b-Hydroxy-5-cholenoic acid | 0.689099212 | 0.606293386 | 0.599295278 |
| *Pseudomonas putida* | K16881 | 3b-Hydroxy-5-cholenoic acid | 0.501803785 | 0.589625535 | 0.60521494 |
| *Mordavella sp. Marseille-P3756* | K16881 | 3b-Hydroxy-5-cholenoic acid | 0.506421611 | 0.589625535 | 0.5863284 |
| *Ketobacter alkanivorans* | K16881 | 3b-Hydroxy-5-cholenoic acid | 0.507254407 | 0.589625535 | 0.506760393 |
| *Hymenobacter sedentarius* | K16881 | 3b-Hydroxy-5-cholenoic acid | 0.509065641 | 0.589625535 | 0.670849531 |
| *Oxalobacter formigenes* | K16881 | 3b-Hydroxy-5-cholenoic acid | 0.509808017 | 0.589625535 | 0.652290345 |
| *Bifidobacterium choerinum* | K16881 | 3b-Hydroxy-5-cholenoic acid | 0.520841332 | 0.589625535 | 0.571186924 |
| *Mucilaginibacter mallensis* | K16881 | 3b-Hydroxy-5-cholenoic acid | 0.523486303 | 0.589625535 | 0.522985052 |
| *butyrate-producing bacterium SM4/1* | K16881 | 3b-Hydroxy-5-cholenoic acid | 0.525508626 | 0.589625535 | 0.502184637 |
| *Deinococcus proteolyticus* | K16881 | 3b-Hydroxy-5-cholenoic acid | 0.529521959 | 0.589625535 | 0.561792631 |
| *Acidaminococcus fermentans* | K16881 | 3b-Hydroxy-5-cholenoic acid | 0.536180936 | 0.589625535 | 0.578858351 |
| *Geobacter metallireducens* | K16881 | 3b-Hydroxy-5-cholenoic acid | 0.537829882 | 0.589625535 | 0.509685593 |
| *Roseiflexus castenholzii* | K16881 | 3b-Hydroxy-5-cholenoic acid | 0.54004128 | 0.589625535 | 0.537800504 |
| *Pantoea gaviniae* | K16881 | 3b-Hydroxy-5-cholenoic acid | 0.543862455 | 0.589625535 | 0.542132254 |
| *Thermincola potens* | K16881 | 3b-Hydroxy-5-cholenoic acid | 0.544287786 | 0.589625535 | 0.571247357 |
| *Xanthomonas oryzae* | K16881 | 3b-Hydroxy-5-cholenoic acid | 0.546058286 | 0.589625535 | 0.528418113 |
| *Streptomyces violaceoruber* | K16881 | 3b-Hydroxy-5-cholenoic acid | 0.547134527 | 0.589625535 | 0.516177207 |
| *Luteitalea pratensis* | K16881 | 3b-Hydroxy-5-cholenoic acid | 0.564210142 | 0.589625535 | 0.599034498 |
| *Synechococcus sp. SynAce01* | K16881 | 3b-Hydroxy-5-cholenoic acid | 0.565893462 | 0.589625535 | 0.554008598 |
| *Bifidobacterium scardovii* | K16881 | 3b-Hydroxy-5-cholenoic acid | 0.566042879 | 0.589625535 | 0.593375617 |
| *Kiritimatiella glycovorans* | K16881 | 3b-Hydroxy-5-cholenoic acid | 0.575572266 | 0.589625535 | 0.519280963 |
| *Thermus thermophilus* | K16881 | 3b-Hydroxy-5-cholenoic acid | 0.585811858 | 0.589625535 | 0.614186547 |
| *Olsenella umbonata* | K16881 | 3b-Hydroxy-5-cholenoic acid | 0.587182261 | 0.589625535 | 0.594362227 |
| *Gordonibacter urolithinfaciens* | K16881 | 3b-Hydroxy-5-cholenoic acid | 0.592005324 | 0.589625535 | 0.566173362 |
| *Stigmatella aurantiaca* | K16881 | 3b-Hydroxy-5-cholenoic acid | 0.592544567 | 0.589625535 | 0.531876014 |
| *Aeromonas sp. CU5* | K16881 | 3b-Hydroxy-5-cholenoic acid | 0.600207855 | 0.589625535 | 0.549479379 |
| *Slackia heliotrinireducens* | K16881 | 3b-Hydroxy-5-cholenoic acid | 0.600317411 | 0.589625535 | 0.599295278 |
| *Chromohalobacter salexigens* | K16881 | 3b-Hydroxy-5-cholenoic acid | 0.604747162 | 0.589625535 | 0.609827901 |
| *Gemmatirosa kalamazoonesis* | K16881 | 3b-Hydroxy-5-cholenoic acid | 0.611774413 | 0.589625535 | 0.544803213 |
| *Clostridium sp. enrichment culture clone 7-14* | K16881 | 3b-Hydroxy-5-cholenoic acid | 0.611774413 | 0.589625535 | 0.611670924 |
| *Plantactinospora sp. KBS50* | K16881 | 3b-Hydroxy-5-cholenoic acid | 0.611918892 | 0.589625535 | 0.519691721 |
| *Acutalibacter muris* | K16881 | 3b-Hydroxy-5-cholenoic acid | 0.612548935 | 0.589625535 | 0.500476983 |
| *Paenibacillus mucilaginosus* | K16881 | 3b-Hydroxy-5-cholenoic acid | 0.61293947 | 0.589625535 | 0.576603242 |
| *Serratia marcescens* | K16881 | 3b-Hydroxy-5-cholenoic acid | 0.619096572 | 0.589625535 | 0.596899225 |
| *Phreatobacter cathodiphilus* | K16881 | 3b-Hydroxy-5-cholenoic acid | 0.625841485 | 0.589625535 | 0.548613563 |
| *Oceanithermus profundus* | K16881 | 3b-Hydroxy-5-cholenoic acid | 0.627723445 | 0.589625535 | 0.520707555 |
| *Gordonibacter pamelaeae* | K16881 | 3b-Hydroxy-5-cholenoic acid | 0.646495674 | 0.589625535 | 0.650599013 |
| *Pseudomonas citronellolis* | K16881 | 3b-Hydroxy-5-cholenoic acid | 0.651010882 | 0.589625535 | 0.599295278 |
| *Azospira oryzae* | K16881 | 3b-Hydroxy-5-cholenoic acid | 0.652206239 | 0.589625535 | 0.601430636 |
| *Desulfovibrio salexigens* | K16881 | 3b-Hydroxy-5-cholenoic acid | 0.655958101 | 0.589625535 | 0.520727157 |
| *Thermoplasmatales archaeon BRNA1* | K16881 | 3b-Hydroxy-5-cholenoic acid | 0.656346749 | 0.589625535 | 0.544471476 |
| *Geobacter uraniireducens* | K16881 | 3b-Hydroxy-5-cholenoic acid | 0.658530238 | 0.589625535 | 0.53173497 |
| *Acidovorax avenae* | K16881 | 3b-Hydroxy-5-cholenoic acid | 0.662914612 | 0.589625535 | 0.612684989 |
| *Eggerthella sp. YY7918* | K16881 | 3b-Hydroxy-5-cholenoic acid | 0.665480071 | 0.589625535 | 0.657505285 |
| *endosymbiont of unidentified scaly snail isolate Monju* | K16881 | 3b-Hydroxy-5-cholenoic acid | 0.671571062 | 0.589625535 | 0.535094959 |
| *Rhodothermus marinus* | K16881 | 3b-Hydroxy-5-cholenoic acid | 0.675708242 | 0.589625535 | 0.552309807 |
| *Clostridiales bacterium CoAT_53-4c* | K16881 | 3b-Hydroxy-5-cholenoic acid | 0.687365608 | 0.589625535 | 0.507193995 |
| *Gloeobacter violaceus* | K16881 | 3b-Hydroxy-5-cholenoic acid | 0.690609994 | 0.589625535 | 0.597210283 |
| *Labrenzia sp. VG12* | K16881 | 3b-Hydroxy-5-cholenoic acid | 0.703440098 | 0.589625535 | 0.553757475 |
| *Thermaerobacter marianensis* | K16881 | 3b-Hydroxy-5-cholenoic acid | 0.710837386 | 0.589625535 | 0.6136716 |
| *Streptomyces gilvosporeus* | K16881 | 3b-Hydroxy-5-cholenoic acid | 0.715280482 | 0.589625535 | 0.521609083 |
| *Acidovorax carolinensis* | K16881 | 3b-Hydroxy-5-cholenoic acid | 0.71817016 | 0.589625535 | 0.688187379 |
| *Ruminococcus champanellensis* | K16881 | 3b-Hydroxy-5-cholenoic acid | 0.758144451 | 0.589625535 | 0.597744891 |
| *Moorella thermoacetica* | K16881 | 3b-Hydroxy-5-cholenoic acid | 0.781028345 | 0.589625535 | 0.518111346 |
| *Clostridiales bacterium CoAT_53-4c* | K07404 | 3b-Hydroxy-5-cholenoic acid | -0.511841561 | -0.539675828 | 0.507193995 |
| *Slackia heliotrinireducens* | K07404 | 3b-Hydroxy-5-cholenoic acid | -0.538367347 | -0.539675828 | 0.599295278 |
| *Candidatus Symbiobacter mobilis* | K07404 | 3b-Hydroxy-5-cholenoic acid | -0.588451986 | -0.539675828 | 0.541450614 |
| *Gemmatirosa kalamazoonesis* | K07404 | 3b-Hydroxy-5-cholenoic acid | -0.610073338 | -0.539675828 | 0.544803213 |
| *Corynebacterium frankenforstense* | K03322 | 3b-Hydroxy-5-cholenoic acid | -0.505765897 | -0.561381254 | 0.529616971 |
| *Thermincola potens* | K03322 | 3b-Hydroxy-5-cholenoic acid | -0.513469388 | -0.561381254 | 0.571247357 |
| *[Eubacterium] rectale* | K03322 | 3b-Hydroxy-5-cholenoic acid | -0.514285714 | -0.561381254 | 0.541649049 |
| *Comamonas kerstersii* | K03322 | 3b-Hydroxy-5-cholenoic acid | -0.510526346 | -0.561381254 | 0.635275793 |
| *Mucilaginibacter mallensis* | K03322 | 3b-Hydroxy-5-cholenoic acid | -0.510627949 | -0.561381254 | 0.522985052 |
| *Gloeobacter violaceus* | K03322 | 3b-Hydroxy-5-cholenoic acid | -0.521075489 | -0.561381254 | 0.597210283 |
| *Plantactinospora sp. KBS50* | K03322 | 3b-Hydroxy-5-cholenoic acid | -0.521094386 | -0.561381254 | 0.519691721 |
| *Gordonibacter urolithinfaciens* | K03322 | 3b-Hydroxy-5-cholenoic acid | -0.533163265 | -0.561381254 | 0.566173362 |
| *Chromohalobacter salexigens* | K03322 | 3b-Hydroxy-5-cholenoic acid | -0.530229071 | -0.561381254 | 0.609827901 |
| *Azotobacter chroococcum* | K03322 | 3b-Hydroxy-5-cholenoic acid | -0.540102041 | -0.561381254 | 0.519802678 |
| *Ketobacter alkanivorans* | K03322 | 3b-Hydroxy-5-cholenoic acid | -0.53406404 | -0.561381254 | 0.506760393 |
| *Cryobacterium arcticum* | K03322 | 3b-Hydroxy-5-cholenoic acid | -0.539152699 | -0.561381254 | 0.526798438 |
| *Geobacter uraniireducens* | K03322 | 3b-Hydroxy-5-cholenoic acid | -0.539458978 | -0.561381254 | 0.53173497 |
| *Azospira oryzae* | K03322 | 3b-Hydroxy-5-cholenoic acid | -0.545463826 | -0.561381254 | 0.601430636 |
| *Rhodothermus marinus* | K03322 | 3b-Hydroxy-5-cholenoic acid | -0.546586389 | -0.561381254 | 0.552309807 |
| *Streptomyces violaceoruber* | K03322 | 3b-Hydroxy-5-cholenoic acid | -0.553222364 | -0.561381254 | 0.516177207 |
| *Aeromonas sp. CU5* | K03322 | 3b-Hydroxy-5-cholenoic acid | -0.554966792 | -0.561381254 | 0.549479379 |
| *Slackia heliotrinireducens* | K03322 | 3b-Hydroxy-5-cholenoic acid | -0.567755102 | -0.561381254 | 0.599295278 |
| *Desulfovibrio salexigens* | K03322 | 3b-Hydroxy-5-cholenoic acid | -0.561726255 | -0.561381254 | 0.520727157 |
| *Corynebacterium imitans* | K03322 | 3b-Hydroxy-5-cholenoic acid | -0.567288352 | -0.561381254 | 0.515182478 |
| *Gordonibacter pamelaeae* | K03322 | 3b-Hydroxy-5-cholenoic acid | -0.582653061 | -0.561381254 | 0.650599013 |
| *Olsenella umbonata* | K03322 | 3b-Hydroxy-5-cholenoic acid | -0.58877551 | -0.561381254 | 0.594362227 |
| *Oxalobacter formigenes* | K03322 | 3b-Hydroxy-5-cholenoic acid | -0.598061224 | -0.561381254 | 0.652290345 |
| *Thermoplasmatales archaeon BRNA1* | K03322 | 3b-Hydroxy-5-cholenoic acid | -0.59416031 | -0.561381254 | 0.544471476 |
| *Pseudomonas citronellolis* | K03322 | 3b-Hydroxy-5-cholenoic acid | -0.608061224 | -0.561381254 | 0.599295278 |
| *Labrenzia sp. VG12* | K03322 | 3b-Hydroxy-5-cholenoic acid | -0.598135227 | -0.561381254 | 0.553757475 |
| *endosymbiont of unidentified scaly snail isolate Monju* | K03322 | 3b-Hydroxy-5-cholenoic acid | -0.603515843 | -0.561381254 | 0.535094959 |
| *Clostridium sp. enrichment culture clone 7-14* | K03322 | 3b-Hydroxy-5-cholenoic acid | -0.609714682 | -0.561381254 | 0.611670924 |
| *Eggerthella sp. YY7918* | K03322 | 3b-Hydroxy-5-cholenoic acid | -0.626326531 | -0.561381254 | 0.657505285 |
| *Bifidobacterium scardovii* | K03322 | 3b-Hydroxy-5-cholenoic acid | -0.629693878 | -0.561381254 | 0.593375617 |
| *Lachnoclostridium phocaeense* | K03322 | 3b-Hydroxy-5-cholenoic acid | -0.631836735 | -0.561381254 | 0.519520789 |
| *Synechococcus sp. SynAce01* | K03322 | 3b-Hydroxy-5-cholenoic acid | -0.622626851 | -0.561381254 | 0.554008598 |
| *Moorella thermoacetica* | K03322 | 3b-Hydroxy-5-cholenoic acid | -0.637755102 | -0.561381254 | 0.518111346 |
| *Paenibacillus mucilaginosus* | K03322 | 3b-Hydroxy-5-cholenoic acid | -0.638265306 | -0.561381254 | 0.576603242 |
| *Acidovorax avenae* | K03322 | 3b-Hydroxy-5-cholenoic acid | -0.641530612 | -0.561381254 | 0.612684989 |
| *Acidovorax carolinensis* | K03322 | 3b-Hydroxy-5-cholenoic acid | -0.628969188 | -0.561381254 | 0.688187379 |
| *Luteitalea pratensis* | K03322 | 3b-Hydroxy-5-cholenoic acid | -0.632567158 | -0.561381254 | 0.599034498 |
| *Pantoea sp. PSNIH1* | K03322 | 3b-Hydroxy-5-cholenoic acid | -0.6348528 | -0.561381254 | 0.602821024 |
| *Acidaminococcus fermentans* | K03322 | 3b-Hydroxy-5-cholenoic acid | -0.649693878 | -0.561381254 | 0.578858351 |
| *Serratia marcescens* | K03322 | 3b-Hydroxy-5-cholenoic acid | -0.652959184 | -0.561381254 | 0.596899225 |
| *Mordavella sp. Marseille-P3756* | K03322 | 3b-Hydroxy-5-cholenoic acid | -0.672755102 | -0.561381254 | 0.5863284 |
| *butyrate-producing bacterium SM4/1* | K03322 | 3b-Hydroxy-5-cholenoic acid | -0.676938776 | -0.561381254 | 0.502184637 |
| *Ruminococcus champanellensis* | K03322 | 3b-Hydroxy-5-cholenoic acid | -0.68877551 | -0.561381254 | 0.597744891 |
| *Thermaerobacter marianensis* | K03322 | 3b-Hydroxy-5-cholenoic acid | -0.705510204 | -0.561381254 | 0.6136716 |
| *Gordonibacter urolithinfaciens* | K03322 | L-Pipecolic acid | -0.533163265 | -0.506131078 | 0.554474982 |
| *Rhodothermus marinus* | K03322 | L-Pipecolic acid | -0.546586389 | -0.506131078 | 0.553437401 |
| *Gordonibacter pamelaeae* | K03322 | L-Pipecolic acid | -0.582653061 | -0.506131078 | 0.573784355 |
| *Clostridium sp. enrichment culture clone 7-14* | K03322 | L-Pipecolic acid | -0.609714682 | -0.506131078 | 0.515053996 |
| *Eggerthella sp. YY7918* | K03322 | L-Pipecolic acid | -0.626326531 | -0.506131078 | 0.545172657 |
| *Bifidobacterium scardovii* | K03322 | L-Pipecolic acid | -0.629693878 | -0.506131078 | 0.520648344 |
| *Paenibacillus mucilaginosus* | K03322 | L-Pipecolic acid | -0.638265306 | -0.506131078 | 0.507258633 |
| *Acidovorax avenae* | K03322 | L-Pipecolic acid | -0.641530612 | -0.506131078 | 0.530796335 |
| *Acidovorax carolinensis* | K03322 | L-Pipecolic acid | -0.628969188 | -0.506131078 | 0.539707626 |
| *Mordavella sp. Marseille-P3756* | K03322 | L-Pipecolic acid | -0.672755102 | -0.506131078 | 0.558844257 |
| *Ruminococcus champanellensis* | K03322 | L-Pipecolic acid | -0.68877551 | -0.506131078 | 0.543763214 |
| *Kiritimatiella glycovorans* | K01885 | 3b-Hydroxy-5-cholenoic acid | 0.506585058 | 0.505567301 | 0.519280963 |
| *Thermoplasmatales archaeon BRNA1* | K01885 | 3b-Hydroxy-5-cholenoic acid | 0.510628964 | 0.505567301 | 0.544471476 |
| *Actinomyces succiniciruminis* | K01885 | 3b-Hydroxy-5-cholenoic acid | 0.518979592 | 0.505567301 | 0.550669486 |
| *Pectobacterium polaris* | K01885 | 3b-Hydroxy-5-cholenoic acid | 0.515409372 | 0.505567301 | 0.522138341 |
| *Desulfovibrio salexigens* | K01885 | 3b-Hydroxy-5-cholenoic acid | 0.517707343 | 0.505567301 | 0.520727157 |
| *Bifidobacterium choerinum* | K01885 | 3b-Hydroxy-5-cholenoic acid | 0.520869479 | 0.505567301 | 0.571186924 |
| *Plantactinospora sp. KBS50* | K01885 | 3b-Hydroxy-5-cholenoic acid | 0.522795224 | 0.505567301 | 0.519691721 |
| *Thermaerobacter marianensis* | K01885 | 3b-Hydroxy-5-cholenoic acid | 0.531734694 | 0.505567301 | 0.6136716 |
| *Azospira oryzae* | K01885 | 3b-Hydroxy-5-cholenoic acid | 0.527196655 | 0.505567301 | 0.601430636 |
| *Clostridiales bacterium CoAT_53-4c* | K01885 | 3b-Hydroxy-5-cholenoic acid | 0.527250686 | 0.505567301 | 0.507193995 |
| *Stigmatella aurantiaca* | K01885 | 3b-Hydroxy-5-cholenoic acid | 0.527284415 | 0.505567301 | 0.531876014 |
| *Pseudomonas citronellolis* | K01885 | 3b-Hydroxy-5-cholenoic acid | 0.534693878 | 0.505567301 | 0.599295278 |
| *Corynebacterium frankenforstense* | K01885 | 3b-Hydroxy-5-cholenoic acid | 0.535156651 | 0.505567301 | 0.529616971 |
| *Paenibacillus mucilaginosus* | K01885 | 3b-Hydroxy-5-cholenoic acid | 0.547857143 | 0.505567301 | 0.576603242 |
| *Gordonibacter pamelaeae* | K01885 | 3b-Hydroxy-5-cholenoic acid | 0.55755102 | 0.505567301 | 0.650599013 |
| *Acidovorax avenae* | K01885 | 3b-Hydroxy-5-cholenoic acid | 0.559591837 | 0.505567301 | 0.612684989 |
| *Cryobacterium arcticum* | K01885 | 3b-Hydroxy-5-cholenoic acid | 0.553445708 | 0.505567301 | 0.526798438 |
| *Gordonibacter urolithinfaciens* | K01885 | 3b-Hydroxy-5-cholenoic acid | 0.56255102 | 0.505567301 | 0.566173362 |
| *Rhodothermus marinus* | K01885 | 3b-Hydroxy-5-cholenoic acid | 0.556179205 | 0.505567301 | 0.552309807 |
| *Ruminococcus champanellensis* | K01885 | 3b-Hydroxy-5-cholenoic acid | 0.56622449 | 0.505567301 | 0.597744891 |
| *Deinococcus proteolyticus* | K01885 | 3b-Hydroxy-5-cholenoic acid | 0.558560162 | 0.505567301 | 0.561792631 |
| *Gemmatirosa kalamazoonesis* | K01885 | 3b-Hydroxy-5-cholenoic acid | 0.567137128 | 0.505567301 | 0.544803213 |
| *Acidovorax carolinensis* | K01885 | 3b-Hydroxy-5-cholenoic acid | 0.572060015 | 0.505567301 | 0.688187379 |
| *Xanthomonas oryzae* | K01885 | 3b-Hydroxy-5-cholenoic acid | 0.57321611 | 0.505567301 | 0.528418113 |
| *Serratia marcescens* | K01885 | 3b-Hydroxy-5-cholenoic acid | 0.586326531 | 0.505567301 | 0.596899225 |
| *Slackia heliotrinireducens* | K01885 | 3b-Hydroxy-5-cholenoic acid | 0.592653061 | 0.505567301 | 0.599295278 |
| *Bifidobacterium scardovii* | K01885 | 3b-Hydroxy-5-cholenoic acid | 0.611020408 | 0.505567301 | 0.593375617 |
| *Olsenella umbonata* | K01885 | 3b-Hydroxy-5-cholenoic acid | 0.630102041 | 0.505567301 | 0.594362227 |
| *Clostridium sp. enrichment culture clone 7-14* | K01885 | 3b-Hydroxy-5-cholenoic acid | 0.635691602 | 0.505567301 | 0.611670924 |
| *Paenibacillus mucilaginosus* | K01885 | L-Pipecolic acid | 0.547857143 | 0.515010571 | 0.507258633 |
| *Gordonibacter pamelaeae* | K01885 | L-Pipecolic acid | 0.55755102 | 0.515010571 | 0.573784355 |
| *Acidovorax avenae* | K01885 | L-Pipecolic acid | 0.559591837 | 0.515010571 | 0.530796335 |
| *Gordonibacter urolithinfaciens* | K01885 | L-Pipecolic acid | 0.56255102 | 0.515010571 | 0.554474982 |
| *Rhodothermus marinus* | K01885 | L-Pipecolic acid | 0.556179205 | 0.515010571 | 0.553437401 |
| *Ruminococcus champanellensis* | K01885 | L-Pipecolic acid | 0.56622449 | 0.515010571 | 0.543763214 |
| *Deinococcus proteolyticus* | K01885 | L-Pipecolic acid | 0.558560162 | 0.515010571 | 0.502452315 |
| *Sulfurifustis variabilis* | K01885 | L-Pipecolic acid | 0.570938664 | 0.515010571 | 0.516934312 |
| *Acidovorax carolinensis* | K01885 | L-Pipecolic acid | 0.572060015 | 0.515010571 | 0.539707626 |
| *Bifidobacterium scardovii* | K01885 | L-Pipecolic acid | 0.611020408 | 0.515010571 | 0.520648344 |
| *Clostridium sp. enrichment culture clone 7-14* | K01885 | L-Pipecolic acid | 0.635691602 | 0.515010571 | 0.515053996 |
| *endosymbiont of unidentified scaly snail isolate Monju* | K07814 | 3b-Hydroxy-5-cholenoic acid | 0.500613633 | 0.556589147 | 0.535094959 |
| *Actinomyces succiniciruminis* | K07814 | 3b-Hydroxy-5-cholenoic acid | 0.510510204 | 0.556589147 | 0.550669486 |
| *Rhodoferax ferrireducens* | K07814 | 3b-Hydroxy-5-cholenoic acid | 0.505359943 | 0.556589147 | 0.525529045 |
| *Gordonibacter pamelaeae* | K07814 | 3b-Hydroxy-5-cholenoic acid | 0.515102041 | 0.556589147 | 0.650599013 |
| *Acidovorax avenae* | K07814 | 3b-Hydroxy-5-cholenoic acid | 0.517959184 | 0.556589147 | 0.612684989 |
| *Phreatobacter cathodiphilus* | K07814 | 3b-Hydroxy-5-cholenoic acid | 0.517162624 | 0.556589147 | 0.548613563 |
| *butyrate-producing bacterium SM4/1* | K07814 | 3b-Hydroxy-5-cholenoic acid | 0.52877551 | 0.556589147 | 0.502184637 |
| *Thermaerobacter marianensis* | K07814 | 3b-Hydroxy-5-cholenoic acid | 0.534795918 | 0.556589147 | 0.6136716 |
| *Gordonibacter urolithinfaciens* | K07814 | 3b-Hydroxy-5-cholenoic acid | 0.537959184 | 0.556589147 | 0.566173362 |
| *Moorella thermoacetica* | K07814 | 3b-Hydroxy-5-cholenoic acid | 0.540612245 | 0.556589147 | 0.518111346 |
| *Pseudomonas putida* | K07814 | 3b-Hydroxy-5-cholenoic acid | 0.545306122 | 0.556589147 | 0.60521494 |
| *Hymenobacter sedentarius* | K07814 | 3b-Hydroxy-5-cholenoic acid | 0.53942334 | 0.556589147 | 0.670849531 |
| *Cryobacterium arcticum* | K07814 | 3b-Hydroxy-5-cholenoic acid | 0.540275721 | 0.556589147 | 0.526798438 |
| *Methanocorpusculum labreanum* | K07814 | 3b-Hydroxy-5-cholenoic acid | 0.54939282 | 0.556589147 | 0.509049458 |
| *Streptomyces violaceoruber* | K07814 | 3b-Hydroxy-5-cholenoic acid | 0.5494113 | 0.556589147 | 0.516177207 |
| *Corynebacterium imitans* | K07814 | 3b-Hydroxy-5-cholenoic acid | 0.551028983 | 0.556589147 | 0.515182478 |
| *Olsenella umbonata* | K07814 | 3b-Hydroxy-5-cholenoic acid | 0.559285714 | 0.556589147 | 0.594362227 |
| *Gloeobacter violaceus* | K07814 | 3b-Hydroxy-5-cholenoic acid | 0.553098319 | 0.556589147 | 0.597210283 |
| *Pantoea sp. PSNIH1* | K07814 | 3b-Hydroxy-5-cholenoic acid | 0.553811022 | 0.556589147 | 0.602821024 |
| *Acidovorax carolinensis* | K07814 | 3b-Hydroxy-5-cholenoic acid | 0.570934114 | 0.556589147 | 0.688187379 |
| *Mordavella sp. Marseille-P3756* | K07814 | 3b-Hydroxy-5-cholenoic acid | 0.58255102 | 0.556589147 | 0.5863284 |
| *Ruminococcus champanellensis* | K07814 | 3b-Hydroxy-5-cholenoic acid | 0.585 | 0.556589147 | 0.597744891 |
| *Desulfovibrio salexigens* | K07814 | 3b-Hydroxy-5-cholenoic acid | 0.576484336 | 0.556589147 | 0.520727157 |
| *Synechococcus sp. SynAce01* | K07814 | 3b-Hydroxy-5-cholenoic acid | 0.581495173 | 0.556589147 | 0.554008598 |
| *Labrenzia sp. VG12* | K07814 | 3b-Hydroxy-5-cholenoic acid | 0.586863191 | 0.556589147 | 0.553757475 |
| *Deinococcus proteolyticus* | K07814 | 3b-Hydroxy-5-cholenoic acid | 0.59042536 | 0.556589147 | 0.561792631 |
| *Azotobacter chroococcum* | K07814 | 3b-Hydroxy-5-cholenoic acid | 0.608265306 | 0.556589147 | 0.519802678 |
| *Kiritimatiella glycovorans* | K07814 | 3b-Hydroxy-5-cholenoic acid | 0.601225193 | 0.556589147 | 0.519280963 |
| *Bradyrhizobium sp. BTAi1* | K07814 | 3b-Hydroxy-5-cholenoic acid | 0.601875494 | 0.556589147 | 0.52498704 |
| *Paenibacillus mucilaginosus* | K07814 | 3b-Hydroxy-5-cholenoic acid | 0.616020408 | 0.556589147 | 0.576603242 |
| *Ketobacter alkanivorans* | K07814 | 3b-Hydroxy-5-cholenoic acid | 0.609152311 | 0.556589147 | 0.506760393 |
| *Acidaminococcus fermentans* | K07814 | 3b-Hydroxy-5-cholenoic acid | 0.620510204 | 0.556589147 | 0.578858351 |
| *Slackia heliotrinireducens* | K07814 | 3b-Hydroxy-5-cholenoic acid | 0.628163265 | 0.556589147 | 0.599295278 |
| *Thermincola potens* | K07814 | 3b-Hydroxy-5-cholenoic acid | 0.633877551 | 0.556589147 | 0.571247357 |
| *Luteitalea pratensis* | K07814 | 3b-Hydroxy-5-cholenoic acid | 0.625322075 | 0.556589147 | 0.599034498 |
| *Lachnoclostridium phocaeense* | K07814 | 3b-Hydroxy-5-cholenoic acid | 0.640102041 | 0.556589147 | 0.519520789 |
| *Microbacterium sp. XT11* | K07814 | 3b-Hydroxy-5-cholenoic acid | 0.627220951 | 0.556589147 | 0.511294374 |
| *Rhodothermus marinus* | K07814 | 3b-Hydroxy-5-cholenoic acid | 0.630166347 | 0.556589147 | 0.552309807 |
| *Bifidobacterium scardovii* | K07814 | 3b-Hydroxy-5-cholenoic acid | 0.666734694 | 0.556589147 | 0.593375617 |
| *Azospira oryzae* | K07814 | 3b-Hydroxy-5-cholenoic acid | 0.6511889 | 0.556589147 | 0.601430636 |
| *Serratia marcescens* | K07814 | 3b-Hydroxy-5-cholenoic acid | 0.684081633 | 0.556589147 | 0.596899225 |
| *Thermoplasmatales archaeon BRNA1* | K07814 | 3b-Hydroxy-5-cholenoic acid | 0.695752487 | 0.556589147 | 0.544471476 |
| *Clostridium sp. enrichment culture clone 7-14* | K07814 | 3b-Hydroxy-5-cholenoic acid | 0.562423367 | 0.556589147 | 0.611670924 |
| *Chromohalobacter salexigens* | K07814 | 3b-Hydroxy-5-cholenoic acid | 0.565427169 | 0.556589147 | 0.609827901 |
| *Rhodoferax ferrireducens* | K07814 | Taurocholate | 0.505359943 | -0.529668781 | -0.510860494 |
| *Synechococcus sp. JA-2-3B'a(2-13)* | K07814 | Taurocholate | 0.526029904 | -0.529668781 | -0.500725508 |
| *Verminephrobacter eiseniae* | K07814 | Taurocholate | 0.566832858 | -0.529668781 | -0.527080526 |
| *Ruminococcus champanellensis* | K07814 | Taurocholate | 0.585 | -0.529668781 | -0.548132488 |
| *Ottowia sp. oral taxon 894* | K07814 | Taurocholate | 0.585455492 | -0.529668781 | -0.502114468 |
| *butyrate-producing bacterium SS3/4* | K07814 | Taurocholate | 0.601938776 | -0.529668781 | -0.520789288 |
| *Paenibacillus mucilaginosus* | K07814 | Taurocholate | 0.616020408 | -0.529668781 | -0.540803383 |
| *Oxalobacter formigenes* | K07814 | Taurocholate | 0.687040816 | -0.529668781 | -0.501057082 |

**Table S7. Spearman correlation between species and clinical indicators**

| **Species** | **Clinical indicators** | **R value** | ***P* value** | **Relation** |
| --- | --- | --- | --- | --- |
| *Barnesiella viscericola* | HGB | 0.5287198 | 9.41E-05 | positive |
| *Microbacterium aurum* | HGB | 0.5045452 | 0.000218 | positive |
| *Corynebacterium glutamicum* | CO2 | 0.4823155 | 0.000449 | positive |
| *Bifidobacterium choerinum* | HGB | 0.4816639 | 0.000458 | positive |
| *Hymenobacter sp. APR13* | CO2 | 0.481491 | 0.000461 | positive |
| *Gordonibacter pamelaeae* | CO2 | 0.4761501 | 0.000544 | positive |
| *Prevotella fusca* | HGB | 0.4739085 | 0.000582 | positive |
| *Agromyces sp. 30A* | HGB | 0.473614 | 0.000588 | positive |
| *Eggerthella sp. YY7918* | CO2 | 0.470884 | 0.000639 | positive |
| *Faecalibacterium phage FP_Toutatis* | HGB | 0.4621495 | 0.000829 | positive |
| *Martelella mediterranea* | ALB | 0.4603912 | 0.000873 | positive |
| *Martelella mediterranea* | HGB | 0.4603869 | 0.000873 | positive |
| *Halomonas hydrothermalis* | Cr | 0.4583097 | 0.000928 | positive |
| *Acinetobacter sp. TGL-Y2* | HGB | 0.457662 | 0.000945 | positive |
| *Microbacterium aurum* | ALB | 0.4568391 | 0.000968 | positive |
| *Acutalibacter muris* | CO2 | 0.455335 | 0.001011 | positive |
| *Mucilaginibacter sp. PAMC 26640* | HGB | 0.4525711 | 0.001095 | positive |
| *Hydrogenophaga crassostreae* | HGB | 0.4515429 | 0.001127 | positive |
| *Fervidobacterium pennivorans* | CO2 | 0.4494014 | 0.001198 | positive |
| *Francisella philomiragia* | CO2 | 0.4469036 | 0.001286 | positive |
| *Halomonas hydrothermalis* | BUN | 0.4454482 | 0.001339 | positive |
| *Streptomyces lincolnensis* | HGB | 0.4427533 | 0.001444 | positive |
| *Erysipelotrichaceae bacterium I46* | CysC | 0.4390623 | 0.001797 | positive |
| *Desulfotalea psychrophila* | HGB | 0.4353243 | 0.001771 | positive |
| *Erysipelotrichaceae bacterium I46* | BUN | 0.4348538 | 0.001794 | positive |
| *Acidovorax carolinensis* | CO2 | 0.4341763 | 0.001827 | positive |
| *Mucilaginibacter gotjawali* | HGB | 0.4337108 | 0.00185 | positive |
| *Spirosoma rigui* | CO2 | 0.4333402 | 0.001869 | positive |
| *Acutalibacter muris* | HGB | 0.4294251 | 0.002076 | positive |
| *Hymenobacter sp. PAMC 26554* | HGB | 0.4281811 | 0.002146 | positive |
| *Muricauda ruestringensis* | HGB | 0.4263024 | 0.002256 | positive |
| *Rhodoferax koreense* | CO2 | 0.4253728 | 0.002312 | positive |
| *Aureitalea sp. RR4-38* | HGB | 0.4241664 | 0.002387 | positive |
| *Prevotella ruminicola* | HGB | 0.4237414 | 0.002414 | positive |
| *Frondihabitans sp. 762G35* | ALB | 0.4235455 | 0.002426 | positive |
| *Pseudomonas fulva* | HGB | 0.4229457 | 0.002464 | positive |
| *Hymenobacter sp. APR13* | HGB | 0.4228314 | 0.002472 | positive |
| *Micromonospora viridifaciens* | HGB | 0.4224338 | 0.002498 | positive |
| *Erysipelotrichaceae bacterium I46* | Cr | 0.4215742 | 0.002554 | positive |
| *Acidovorax avenae* | CO2 | 0.4212904 | 0.002573 | positive |
| *Vibrio campbellii* | WBC | 0.4202519 | 0.002644 | positive |
| *Bifidobacterium dentium* | HGB | 0.4171666 | 0.002863 | positive |
| *Gemmatirosa kalamazoonesis* | HGB | 0.4164649 | 0.002915 | positive |
| *Plantibacter flavus* | HGB | 0.4164007 | 0.00292 | positive |
| *Agrococcus carbonis* | HGB | 0.4068235 | 0.003721 | positive |
| *Corynebacterium glutamicum* | WBC | 0.4061328 | 0.003786 | positive |
| *Bifidobacterium choerinum* | ALB | 0.4051137 | 0.003883 | positive |
| *Mucilaginibacter sp. PAMC 26640* | CO2 | 0.4034369 | 0.004048 | positive |
| *Stenotrophomonas acidaminiphila* | ALB | 0.402735 | 0.004118 | positive |
| *Bifidobacterium catenulatum* | HGB | 0.4009288 | 0.004305 | positive |
| *Hymenobacter sp. PAMC 26554* | CO2 | 0.4006964 | 0.00433 | positive |
| *Acidovorax sp. RAC01* | ALB | 0.4005875 | 0.004341 | positive |
| *Microbacterium sp. XT11* | HGB | 0.3970933 | 0.004727 | positive |
| *Vibrio campbellii* | CO2 | 0.3966797 | 0.004775 | positive |
| *Halomonas hydrothermalis* | CysC | 0.3946254 | 0.00551 | positive |
| *Bifidobacterium bifidum* | PLT | 0.3939587 | 0.005098 | positive |
| *Devosia sp. H5989* | ALB | 0.3935215 | 0.005152 | positive |
| *Pseudomonas citronellolis* | CO2 | 0.393068 | 0.005208 | positive |
| *Streptomyces sp. SF2575* | CRP | 0.3926101 | 0.005777 | positive |
| *Libanicoccus massiliensis* | CO2 | 0.3916876 | 0.005383 | positive |
| *[Mycobacterium] stephanolepidis* | BUN | 0.3911222 | 0.005456 | positive |
| *Salmonella bongori* | HGB | 0.3910235 | 0.005468 | positive |
| *Olsenella sp. oral taxon 807* | CO2 | 0.3908184 | 0.005495 | positive |
| *Bifidobacterium dentium* | CO2 | 0.3904041 | 0.00555 | positive |
| *Prevotella jejuni* | HGB | 0.3875577 | 0.005936 | positive |
| *Barnesiella viscericola* | CO2 | 0.38545 | 0.006237 | positive |
| *Spirosoma linguale* | HGB | 0.385371 | 0.006248 | positive |
| *Streptomyces glaucescens* | ALB | 0.383929 | 0.006462 | positive |
| *Planococcus sp. MB-3u-03* | HGB | 0.3822381 | 0.006721 | positive |
| *Aspergillus aculeatus* | HGB | 0.380365 | 0.007018 | positive |
| *Rhodococcus opacus* | CO2 | 0.3787211 | 0.007288 | positive |
| *Desulfovibrio piger* | CO2 | 0.3769117 | 0.007596 | positive |
| *Corynebacterium sphenisci* | CO2 | 0.3764154 | 0.007682 | positive |
| *Tannerella forsythia* | HGB | 0.3756156 | 0.007823 | positive |
| *Rhodococcus virus Pepy6* | CRP | 0.3750347 | 0.008628 | positive |
| *Aspergillus aculeatus* | ALB | 0.3738364 | 0.008145 | positive |
| *Agrococcus carbonis* | CO2 | 0.3722548 | 0.008441 | positive |
| *Sulfurifustis variabilis* | CO2 | 0.3708103 | 0.008718 | positive |
| *Polaribacter sp. KT25b* | HGB | 0.3704447 | 0.00879 | positive |
| *Bifidobacterium dentium* | PLT | 0.3701662 | 0.008845 | positive |
| *Gramella sp. MAR_2010_147* | HGB | 0.3701642 | 0.008845 | positive |
| *Bifidobacterium angulatum* | HGB | 0.3700707 | 0.008864 | positive |
| *Fusobacterium gonidiaformans* | CO2 | 0.3700189 | 0.008874 | positive |
| *Actinomyces radingae* | HGB | 0.3697977 | 0.008918 | positive |
| *Conexibacter woesei* | CO2 | 0.3695199 | 0.008973 | positive |
| *Frondihabitans sp. 762G35* | HGB | 0.3688528 | 0.009108 | positive |
| *Aureitalea sp. RR4-38* | CO2 | 0.3687776 | 0.009123 | positive |
| *Plantactinospora sp. KBS50* | CO2 | 0.3673531 | 0.009416 | positive |
| *Petrimonas sp. IBARAKI* | HGB | 0.3673318 | 0.00942 | positive |
| *Romboutsia ilealis* | HGB | 0.3667866 | 0.009535 | positive |
| *Pantoea gaviniae* | HGB | 0.3663928 | 0.009618 | positive |
| *Agrococcus jejuensis* | HGB | 0.3661255 | 0.009675 | positive |
| *Capnocytophaga stomatis* | HGB | 0.3658669 | 0.00973 | positive |
| *Propionibacterium freudenreichii* | ALB | 0.3649121 | 0.009937 | positive |
| *Lactobacillus fermentum* | BUN | 0.36481 | 0.009959 | positive |
| *Xanthomonas oryzae* | HGB | 0.3647393 | 0.009975 | positive |
| *Flavobacterium commune* | CO2 | 0.3646504 | 0.009994 | positive |
| *Nocardia terpenica* | HGB | 0.3639913 | 0.01014 | positive |
| *Rhizobium sp. ACO-34A* | HGB | 0.3639729 | 0.010144 | positive |
| *[Mycobacterium] stephanolepidis* | CysC | 0.3620481 | 0.01145 | positive |
| *Candidatus Pelagibacter ubique* | ALB | 0.3618062 | 0.010636 | positive |
| *Caldicellulosiruptor bescii* | ALB | 0.3607587 | 0.010881 | positive |
| *Prevotella dentalis* | HGB | 0.3601521 | 0.011025 | positive |
| *Deinococcus proteolyticus* | CO2 | 0.3593381 | 0.011221 | positive |
| *Propionibacterium freudenreichii* | HGB | 0.3590293 | 0.011296 | positive |
| *Collimonas fungivorans* | CO2 | 0.3579526 | 0.011561 | positive |
| *Faecalibacterium phage FP_Taranis* | HGB | 0.3577881 | 0.011602 | positive |
| *Mordavella sp. Marseille-P3756* | CO2 | 0.3576367 | 0.01164 | positive |
| *Fluviicola taffensis* | CO2 | 0.3570465 | 0.011788 | positive |
| *Slackia heliotrinireducens* | CO2 | 0.356972 | 0.011807 | positive |
| *Cutibacterium acnes* | CO2 | 0.3556708 | 0.012141 | positive |
| *[Eubacterium] minutum* | CO2 | 0.3552848 | 0.012241 | positive |
| *Pelagibaca abyssi* | CO2 | 0.3544786 | 0.012453 | positive |
| *Ottowia sp. oral taxon 894* | HGB | 0.3530463 | 0.012838 | positive |
| *Faecalibacterium phage FP_Lugh* | HGB | 0.3526083 | 0.012958 | positive |
| *Erysipelotrichaceae bacterium I46* | PRO | 0.3517561 | 0.013194 | positive |
| *Dictyoglomus turgidum* | HGB | 0.3516852 | 0.013213 | positive |
| *Myxococcus stipitatus* | HGB | 0.3499046 | 0.013718 | positive |
| *Salipiger profundus* | BUN | 0.3497971 | 0.013749 | positive |
| *Vibrio campbellii* | HGB | 0.3497726 | 0.013756 | positive |
| *Thermoplasmatales archaeon BRNA1* | ALB | 0.3490161 | 0.013976 | positive |
| *Agrococcus carbonis* | ALB | 0.3486888 | 0.014072 | positive |
| *Pectobacterium polaris* | CO2 | 0.3472868 | 0.01449 | positive |
| *Bacillus sp. OxB-1* | HGB | 0.3470705 | 0.014556 | positive |
| *Romboutsia ilealis* | TBA | 0.3467253 | 0.01466 | positive |
| *Clostridium sp. enrichment culture clone 7-14* | CO2 | 0.3460589 | 0.014865 | positive |
| *Micromonospora viridifaciens* | ALB | 0.3446896 | 0.015292 | positive |
| *Caldithrix abyssi* | Cr | 0.3442794 | 0.015422 | positive |
| *Faecalibacterium phage FP_Lugh* | ALB | 0.3438194 | 0.015569 | positive |
| *Synechococcus sp. JA-2-3B'a(2-13)* | HGB | 0.3430921 | 0.015804 | positive |
| *Acidovorax carolinensis* | ALB | 0.3430004 | 0.015834 | positive |
| *Enterococcus avium* | BUN | 0.3427802 | 0.015906 | positive |
| *Sterolibacterium denitrificans* | CO2 | 0.3426349 | 0.015954 | positive |
| *Bifidobacterium scardovii* | CO2 | 0.3420939 | 0.016132 | positive |
| *Thermaerobacter marianensis* | CO2 | 0.3416849 | 0.016267 | positive |
| *Clostridiales bacterium CoAT_53-4c* | CO2 | 0.3395501 | 0.016992 | positive |
| *Collimonas fungivorans* | ALB | 0.3394315 | 0.017033 | positive |
| *Pantoea gaviniae* | CO2 | 0.3390761 | 0.017156 | positive |
| *Serratia marcescens* | CO2 | 0.3386684 | 0.017299 | positive |
| *Mycobacterium sp. djl-10* | CO2 | 0.3373304 | 0.017774 | positive |
| *Tessaracoccus sp. T2.5-30* | ALB | 0.3367115 | 0.017997 | positive |
| *Candidatus Phytoplasma australiense* | Cr | 0.3359483 | 0.018276 | positive |
| *Salipiger profundus* | CysC | 0.3359342 | 0.019577 | positive |
| *Caldicellulosiruptor saccharolyticus* | HGB | 0.3358596 | 0.018309 | positive |
| *Fluviicola taffensis* | HGB | 0.335796 | 0.018332 | positive |
| *Enterococcus sp. CR-Ec1* | BUN | 0.3349658 | 0.018641 | positive |
| *Halobacillus mangrovi* | ALB | 0.3348735 | 0.018675 | positive |
| *Fervidobacterium pennivorans* | WBC | 0.3339735 | 0.019015 | positive |
| *Plantibacter flavus* | ALB | 0.3332908 | 0.019276 | positive |
| *Acidovorax sp. RAC01* | HGB | 0.332849 | 0.019446 | positive |
| *Caldicellulosiruptor bescii* | PLT | 0.3308574 | 0.020231 | positive |
| *Corynebacterium uterequi* | HGB | 0.3307384 | 0.020279 | positive |
| *Hymenobacter sedentarius* | HGB | 0.3306108 | 0.02033 | positive |
| *Pseudomonas fulva* | ALB | 0.3304086 | 0.020412 | positive |
| *Mycobacterium sp. djl-10* | HGB | 0.3303378 | 0.02044 | positive |
| *Alkalitalea saponilacus* | HGB | 0.3302284 | 0.020485 | positive |
| *[Mycobacterium] stephanolepidis* | Cr | 0.3300262 | 0.020567 | positive |
| *Tessaracoccus sp. T2.5-30* | CO2 | 0.3296848 | 0.020706 | positive |
| *Sulfurifustis variabilis* | HGB | 0.329389 | 0.020827 | positive |
| *Actinobacillus suis* | HGB | 0.329361 | 0.020838 | positive |
| *Bifidobacterium adolescentis* | HGB | 0.3288678 | 0.021042 | positive |
| *Neomicrococcus aestuarii* | ALB | 0.3285098 | 0.02119 | positive |
| *Sinomonas sp. R1AF57* | WBC | 0.3284468 | 0.021217 | positive |
| *Enterococcus sp. CR-Ec1* | CysC | 0.3275565 | 0.023047 | positive |
| *Ruminococcus champanellensis* | CO2 | 0.3272669 | 0.021714 | positive |
| *Xanthomonas sacchari* | ALB | 0.3268391 | 0.021896 | positive |
| *Agrobacterium rhizogenes* | CO2 | 0.3267408 | 0.021938 | positive |
| *Myxococcus stipitatus* | CO2 | 0.3266681 | 0.02197 | positive |
| *Brachyspira pilosicoli* | HGB | 0.3261119 | 0.02221 | positive |
| *Microbacterium aurum* | WBC | 0.3260673 | 0.022229 | positive |
| *Syntrophus aciditrophicus* | CO2 | 0.3257971 | 0.022346 | positive |
| *Methylosinus trichosporium* | CO2 | 0.3254527 | 0.022497 | positive |
| *Thauera aromatica* | CO2 | 0.3251565 | 0.022627 | positive |
| *Francisella philomiragia* | ALB | 0.3250827 | 0.02266 | positive |
| *Nocardia terpenica* | CO2 | 0.3248139 | 0.022778 | positive |
| *Methanocorpusculum labreanum* | HGB | 0.3237955 | 0.023233 | positive |
| *Vibrio campbellii* | ALB | 0.3234354 | 0.023396 | positive |
| *Sterolibacterium denitrificans* | HGB | 0.3220813 | 0.024016 | positive |
| *Acutalibacter muris* | ALB | 0.3214838 | 0.024294 | positive |
| *Sneathia amnii* | ALB | 0.3214459 | 0.024311 | positive |
| *Corynebacterium sphenisci* | ALB | 0.3211799 | 0.024436 | positive |
| *Rhodoferax ferrireducens* | CO2 | 0.3206818 | 0.024671 | positive |
| *Corynebacterium glyciniphilum* | HGB | 0.320452 | 0.02478 | positive |
| *Methylosinus trichosporium* | HGB | 0.3199992 | 0.024996 | positive |
| *Acidovorax carolinensis* | HGB | 0.3192825 | 0.025341 | positive |
| *Actinoplanes sp. N902-109* | CO2 | 0.3187636 | 0.025593 | positive |
| *Janibacter indicus* | CO2 | 0.3181181 | 0.02591 | positive |
| *Roseiflexus castenholzii* | CO2 | 0.3180132 | 0.025962 | positive |
| *[Eubacterium] rectale* | CO2 | 0.3178084 | 0.026063 | positive |
| *Pseudomonas guangdongensis* | CO2 | 0.3169335 | 0.0265 | positive |
| *Polaribacter sp. KT25b* | CO2 | 0.3159903 | 0.026977 | positive |
| *Bifidobacterium kashiwanohense* | HGB | 0.3157519 | 0.027099 | positive |
| *Erysipelotrichaceae bacterium I46* | CRP | 0.3157439 | 0.028806 | positive |
| *Faecalibacterium phage FP_Taranis* | ALB | 0.3157022 | 0.027125 | positive |
| *Dictyoglomus turgidum* | ALB | 0.315578 | 0.027189 | positive |
| *Tannerella forsythia* | CO2 | 0.3154054 | 0.027277 | positive |
| *Azospira oryzae* | CO2 | 0.315233 | 0.027366 | positive |
| *Virgibacillus phasianinus* | CO2 | 0.3150074 | 0.027483 | positive |
| *Petrimonas sp. IBARAKI* | CO2 | 0.3146465 | 0.02767 | positive |
| *Xanthomonas oryzae* | CO2 | 0.3139731 | 0.028023 | positive |
| *[Eubacterium] sulci* | HGB | 0.3137615 | 0.028135 | positive |
| *Fusobacterium gonidiaformans* | HGB | 0.3133612 | 0.028347 | positive |
| *Bifidobacterium angulatum* | WBC | 0.3131571 | 0.028456 | positive |
| *Verminephrobacter eiseniae* | HGB | 0.3131226 | 0.028474 | positive |
| *Salinicola tamaricis* | HGB | 0.313004 | 0.028538 | positive |
| *Mycolicibacterium rutilum* | CO2 | 0.3129753 | 0.028553 | positive |
| *Xanthomonas sacchari* | HGB | 0.3128886 | 0.028599 | positive |
| *[Eubacterium] rectale* | HGB | 0.3127408 | 0.028679 | positive |
| *Pseudomonas putida* | CO2 | 0.31244 | 0.02884 | positive |
| *Corynebacterium glutamicum* | ALB | 0.3122539 | 0.028941 | positive |
| *Thermotoga sp. RQ7* | HGB | 0.3119656 | 0.029097 | positive |
| *Flavobacterium gilvum* | HGB | 0.3118763 | 0.029146 | positive |
| *Haliscomenobacter hydrossis* | HGB | 0.3112098 | 0.029511 | positive |
| *Paludisphaera borealis* | ALB | 0.3108306 | 0.02972 | positive |
| *Paraburkholderia sprentiae* | CO2 | 0.3106557 | 0.029817 | positive |
| *Lactobacillus acidophilus* | UA | 0.3105929 | 0.029851 | positive |
| *Caldicellulosiruptor bescii* | HGB | 0.3101943 | 0.030073 | positive |
| *Chitinophaga pinensis* | CO2 | 0.3100881 | 0.030133 | positive |
| *Dictyoglomus turgidum* | CO2 | 0.309679 | 0.030362 | positive |
| *Candidatus Symbiobacter mobilis* | HGB | 0.309645 | 0.030381 | positive |
| *Streptomyces lincolnensis* | ALB | 0.3094895 | 0.030469 | positive |
| *Candidatus Pelagibacter ubique* | CO2 | 0.3092008 | 0.030633 | positive |
| *Stigmatella aurantiaca* | HGB | 0.3090488 | 0.030719 | positive |
| *Clostridium chauvoei* | HGB | 0.308607 | 0.030971 | positive |
| *Intrasporangium calvum* | Cr | 0.3083393 | 0.031124 | positive |
| *endosymbiont of unidentified scaly snail isolate Monju* | HGB | 0.3079245 | 0.031364 | positive |
| *Chromohalobacter salexigens* | CO2 | 0.307627 | 0.031536 | positive |
| *Jatrophihabitans sp. GAS493* | HGB | 0.3075756 | 0.031566 | positive |
| *[Eubacterium] siraeum* | CO2 | 0.3072761 | 0.03174 | positive |
| *Enterococcus avium* | CysC | 0.3067797 | 0.033935 | positive |
| *Methanocorpusculum labreanum* | CO2 | 0.3065789 | 0.03215 | positive |
| *Caldithrix abyssi* | BUN | 0.3059808 | 0.032505 | positive |
| *Mucilaginibacter mallensis* | CO2 | 0.3059752 | 0.032508 | positive |
| *Rufibacter tibetensis* | HGB | 0.3059203 | 0.032541 | positive |
| *Porphyrobacter HT-58-2* | CO2 | 0.3057321 | 0.032653 | positive |
| *Devosia sp. H5989* | HGB | 0.3055631 | 0.032754 | positive |
| *Collimonas fungivorans* | WBC | 0.3053295 | 0.032895 | positive |
| *Coprococcus catus* | HGB | 0.3049325 | 0.033134 | positive |
| *Sterolibacterium denitrificans* | ALB | 0.3044181 | 0.033447 | positive |
| *Cryobacterium arcticum* | CO2 | 0.3038011 | 0.033825 | positive |
| *Rhodoferax koreense* | HGB | 0.3037387 | 0.033864 | positive |
| *Kiritimatiella glycovorans* | CO2 | 0.3034942 | 0.034015 | positive |
| *Salinicola tamaricis* | ALB | 0.3032812 | 0.034147 | positive |
| *Geobacter metallireducens* | CO2 | 0.3025285 | 0.034616 | positive |
| *Thermoplasmatales archaeon BRNA1* | HGB | 0.3023472 | 0.03473 | positive |
| *Thermotoga sp. RQ7* | ALB | 0.3022485 | 0.034792 | positive |
| *Enterococcus sp. CR-Ec1* | Cr | 0.3020405 | 0.034924 | positive |
| *Achromobacter sp. AONIH1* | CO2 | 0.3013457 | 0.035365 | positive |
| *Hydrogenophaga crassostreae* | TBA | 0.3012664 | 0.035416 | positive |
| *Actinomyces succiniciruminis* | HGB | 0.3009518 | 0.035618 | positive |
| *Salipiger profundus* | Cr | 0.3002787 | 0.036052 | positive |
| *Bordetella bronchiseptica* | Cr | -0.300003 | 0.036232 | negtive |
| *Pelagibaca abyssi* | PRO | -0.30006 | 0.036195 | negtive |
| *Libanicoccus massiliensis* | BUN | -0.300207 | 0.036099 | negtive |
| *Ottowia sp. oral taxon 894* | CysC | -0.30028 | 0.038109 | negtive |
| *Paenibacillus alvei* | CRP | -0.300936 | 0.037669 | negtive |
| *Libanicoccus massiliensis* | Cr | -0.300972 | 0.035605 | negtive |
| *Pelagibaca abyssi* | BUN | -0.301352 | 0.035361 | negtive |
| *Fervidobacterium pennivorans* | PRO | -0.301363 | 0.035355 | negtive |
| *Xanthomonas phaseoli* | PRO | -0.301368 | 0.035351 | negtive |
| *Bacteroidetes bacterium UKL13-3* | BUN | -0.301387 | 0.035339 | negtive |
| *Lactobacillus terrae* | ALP | -0.301539 | 0.035242 | negtive |
| *Agrococcus jejuensis* | CRP | -0.301928 | 0.037013 | negtive |
| *Dokdonia sp. 4H-3-7-5* | BUN | -0.302019 | 0.034937 | negtive |
| *Streptomyces gilvosporeus* | PLT | -0.302022 | 0.034936 | negtive |
| *Spirosoma linguale* | PRO | -0.302054 | 0.034915 | negtive |
| *Geobacter uraniireducens* | PRO | -0.302091 | 0.034892 | negtive |
| *Prevotella dentalis* | PLT | -0.302217 | 0.034812 | negtive |
| *Pseudoalteromonas piscicida* | WBC | -0.302542 | 0.034608 | negtive |
| *Paraburkholderia sprentiae* | CRP | -0.302579 | 0.036587 | negtive |
| *Acidaminococcus fermentans* | PRO | -0.302691 | 0.034514 | negtive |
| *Vibrio crassostreae* | CRP | -0.302864 | 0.036401 | negtive |
| *Tessaracoccus sp. T2.5-30* | BUN | -0.302964 | 0.034344 | negtive |
| *Thermus thermophilus* | PRO | -0.303103 | 0.034257 | negtive |
| *Bacillus oceanisediminis* | CysC | -0.303178 | 0.036198 | negtive |
| *Ruminococcus champanellensis* | CRP | -0.303339 | 0.036095 | negtive |
| *Thermaerobacter marianensis* | PRO | -0.303446 | 0.034045 | negtive |
| *Romboutsia ilealis* | BUN | -0.303779 | 0.033839 | negtive |
| *Actinomyces succiniciruminis* | BUN | -0.304034 | 0.033682 | negtive |
| *Bifidobacterium pseudocatenulatum* | CysC | -0.304481 | 0.035366 | negtive |
| *Muricauda ruestringensis* | Cr | -0.304619 | 0.033325 | negtive |
| *Faecalibacterium phage FP_Taranis* | PRO | -0.30472 | 0.033263 | negtive |
| *Oxalobacter formigenes* | PRO | -0.305548 | 0.032764 | negtive |
| *Alcanivorax dieselolei* | PRO | -0.305724 | 0.032658 | negtive |
| *Pseudoalteromonas piscicida* | ALP | -0.305865 | 0.032574 | negtive |
| *Cellulomonas fimi* | CRP | -0.306036 | 0.034392 | negtive |
| *Collimonas fungivorans* | CRP | -0.306066 | 0.034374 | negtive |
| *Planococcus sp. PAMC 21323* | Cr | -0.306255 | 0.032342 | negtive |
| *Thermoanaerobacterium saccharolyticum* | BUN | -0.3063 | 0.032315 | negtive |
| *Ruminococcus champanellensis* | PRO | -0.306356 | 0.032282 | negtive |
| *Trichomonas vaginalis* | CysC | -0.306508 | 0.034102 | negtive |
| *Hymenobacter sp. APR13* | CRP | -0.306698 | 0.033985 | negtive |
| *Bifidobacterium catenulatum* | PRO | -0.306787 | 0.032027 | negtive |
| *Bifidobacterium pseudocatenulatum* | Cr | -0.306789 | 0.032026 | negtive |
| *Pantoea sp. PSNIH1* | CRP | -0.306893 | 0.033866 | negtive |
| *Streptococcus himalayensis* | PRO | -0.307003 | 0.0319 | negtive |
| *Terrisporobacter glycolicus* | PRO | -0.307159 | 0.031809 | negtive |
| *Bacillus sp. OxB-1* | Cr | -0.307308 | 0.031722 | negtive |
| *Francisella philomiragia* | UA | -0.307478 | 0.031623 | negtive |
| *Brachybacterium sp. VM2412* | BUN | -0.3075 | 0.03161 | negtive |
| *Geobacter metallireducens* | BUN | -0.307887 | 0.031386 | negtive |
| *Methylobacterium nodulans* | BUN | -0.307979 | 0.031332 | negtive |
| *Gramella sp. MAR_2010_147* | BUN | -0.308503 | 0.03103 | negtive |
| *Bacillus sp. OxB-1* | BUN | -0.308685 | 0.030926 | negtive |
| *Spirosoma rigui* | BUN | -0.308727 | 0.030902 | negtive |
| *Conexibacter woesei* | UA | -0.309363 | 0.030541 | negtive |
| *Mycolicibacterium rutilum* | BUN | -0.30951 | 0.030458 | negtive |
| *Stenotrophomonas acidaminiphila* | PRO | -0.309515 | 0.030455 | negtive |
| *Coprococcus sp. ART55/1* | Cr | -0.309596 | 0.030409 | negtive |
| *Clostridium sp. enrichment culture clone 7-14* | Cr | -0.30978 | 0.030305 | negtive |
| *Thermaerobacter marianensis* | ALP | -0.309996 | 0.030184 | negtive |
| *[Eubacterium] rectale* | CRP | -0.310081 | 0.031965 | negtive |
| *Cellulomonas fimi* | UA | -0.310114 | 0.030118 | negtive |
| *Bartonella vinsonii* | CRP | -0.310205 | 0.031893 | negtive |
| *Paenibacillus alvei* | UA | -0.310418 | 0.029948 | negtive |
| *Sphingobacterium sp. G1-14* | UA | -0.310422 | 0.029946 | negtive |
| *Agrobacterium rhizogenes* | CysC | -0.310639 | 0.031641 | negtive |
| *Romboutsia ilealis* | CysC | -0.31067 | 0.031623 | negtive |
| *Rhodothermus marinus* | UA | -0.310676 | 0.029805 | negtive |
| *Hymenobacter sp. PAMC 26554* | Cr | -0.310704 | 0.02979 | negtive |
| *Francisella philomiragia* | BUN | -0.310774 | 0.029751 | negtive |
| *Ketobacter alkanivorans* | BUN | -0.310863 | 0.029702 | negtive |
| *Gallibacterium anatis* | PRO | -0.310991 | 0.029631 | negtive |
| *Dokdonia sp. 4H-3-7-5* | Cr | -0.311245 | 0.029491 | negtive |
| *Enterococcus avium* | HGB | -0.311447 | 0.02938 | negtive |
| *Cellulomonas fimi* | PRO | -0.311778 | 0.0292 | negtive |
| *Candidatus Saccharibacteria oral taxon TM7x* | CRP | -0.311984 | 0.030872 | negtive |
| *Actinobacillus suis* | PRO | -0.312023 | 0.029066 | negtive |
| *Micromonospora auratinigra* | PRO | -0.312064 | 0.029044 | negtive |
| *Vibrio campbellii* | PRO | -0.312305 | 0.028913 | negtive |
| *Acinetobacter sp. TGL-Y2* | UA | -0.312368 | 0.028879 | negtive |
| *Elizabethkingia anophelis* | UA | -0.312379 | 0.028873 | negtive |
| *Flavobacterium gilvum* | UA | -0.312459 | 0.02883 | negtive |
| *Rhodococcus virus Pepy6* | ALB | -0.31301 | 0.028534 | negtive |
| *Actinomyces radingae* | CRP | -0.31318 | 0.030202 | negtive |
| *Corynebacterium uterequi* | PRO | -0.313227 | 0.028418 | negtive |
| *Arthrobacter sp. YC-RL1* | BUN | -0.31333 | 0.028364 | negtive |
| *Vibrio owensii* | PRO | -0.313469 | 0.02829 | negtive |
| *Dialister sp. Marseille-P5638* | CRP | -0.313669 | 0.029931 | negtive |
| *Agrococcus carbonis* | BUN | -0.314046 | 0.027985 | negtive |
| *Cutibacterium acnes* | Cr | -0.314094 | 0.02796 | negtive |
| *Arthrobacter sp. YC-RL1* | PRO | -0.314316 | 0.027843 | negtive |
| *Bacillus oceanisediminis* | UA | -0.31453 | 0.027731 | negtive |
| *Acidovorax carolinensis* | CysC | -0.314917 | 0.02925 | negtive |
| *Virgibacillus phasianinus* | Cr | -0.315018 | 0.027478 | negtive |
| *Corynebacterium sphenisci* | PRO | -0.315117 | 0.027426 | negtive |
| *Parvimonas micra* | CRP | -0.315192 | 0.029102 | negtive |
| *Candidatus Symbiobacter mobilis* | CRP | -0.315414 | 0.028982 | negtive |
| *Bifidobacterium scardovii* | ALP | -0.315763 | 0.027094 | negtive |
| *Rhodothermus marinus* | PRO | -0.315874 | 0.027037 | negtive |
| *Caulobacter mirabilis* | CysC | -0.316157 | 0.028586 | negtive |
| *Friedmanniella sagamiharensis* | Cr | -0.316172 | 0.026885 | negtive |
| *Clostridium sp. enrichment culture clone 7-14* | BUN | -0.316391 | 0.026774 | negtive |
| *Actinomyces sp. Chiba101* | Cr | -0.316391 | 0.026774 | negtive |
| *Mycolicibacterium rutilum* | CysC | -0.316557 | 0.028374 | negtive |
| *Acutalibacter muris* | CRP | -0.316672 | 0.028313 | negtive |
| *Polaribacter vadi* | BUN | -0.316686 | 0.026624 | negtive |
| *Brachybacterium sp. VM2412* | CRP | -0.317241 | 0.028015 | negtive |
| *Acidovorax carolinensis* | BUN | -0.317305 | 0.026314 | negtive |
| *Erysipelotrichaceae bacterium I46* | TBA | -0.317426 | 0.026253 | negtive |
| *Streptomyces glaucescens* | CysC | -0.317431 | 0.027916 | negtive |
| *Roseiflexus castenholzii* | PRO | -0.317576 | 0.026179 | negtive |
| *[Eubacterium] siraeum* | CysC | -0.317618 | 0.027819 | negtive |
| *Pseudomonas guangdongensis* | PRO | -0.317684 | 0.026125 | negtive |
| *Desulfitobacterium metallireducens* | UA | -0.317839 | 0.026048 | negtive |
| *Polaribacter sp. KT25b* | BUN | -0.317883 | 0.026026 | negtive |
| *Thauera aromatica* | CRP | -0.318041 | 0.027601 | negtive |
| *Serratia grimesii* | BUN | -0.31828 | 0.02583 | negtive |
| *Streptomyces gilvosporeus* | PRO | -0.318321 | 0.02581 | negtive |
| *Rhodoferax antarcticus* | PRO | -0.318403 | 0.02577 | negtive |
| *Dokdonia sp. 4H-3-7-5* | PRO | -0.3185 | 0.025722 | negtive |
| *Caldicellulosiruptor saccharolyticus* | CysC | -0.318595 | 0.027316 | negtive |
| *Mordavella sp. Marseille-P3756* | CRP | -0.318617 | 0.027305 | negtive |
| *Granulosicoccus antarcticus* | ALP | -0.318753 | 0.025599 | negtive |
| *Streptomyces sp. SF2575* | WBC | -0.318855 | 0.025549 | negtive |
| *Brachybacterium sp. VM2412* | PRO | -0.319039 | 0.025459 | negtive |
| *Frondihabitans sp. 762G35* | CysC | -0.319148 | 0.027035 | negtive |
| *Nocardia terpenica* | UA | -0.319386 | 0.025291 | negtive |
| *Virgibacillus phasianinus* | BUN | -0.319441 | 0.025264 | negtive |
| *Desulfobacula toluolica* | PRO | -0.319577 | 0.025198 | negtive |
| *Actinomyces sp. Chiba101* | CRP | -0.319605 | 0.026804 | negtive |
| *Prosthecochloris aestuarii* | BUN | -0.319668 | 0.025155 | negtive |
| *Frondihabitans sp. 762G35* | BUN | -0.31985 | 0.025067 | negtive |
| *Slackia heliotrinireducens* | Cr | -0.319853 | 0.025066 | negtive |
| *Libanicoccus massiliensis* | CysC | -0.319952 | 0.02663 | negtive |
| *Enterococcus avium* | ALB | -0.320081 | 0.024957 | negtive |
| *Sulfurifustis variabilis* | BUN | -0.320393 | 0.024808 | negtive |
| *Flavobacterium commune* | UA | -0.320653 | 0.024684 | negtive |
| *Cellulomonas fimi* | CysC | -0.320911 | 0.026154 | negtive |
| *Salmonella bongori* | BUN | -0.321089 | 0.024479 | negtive |
| *Cladophialophora bantiana* | HGB | -0.321089 | 0.024479 | negtive |
| *Corynebacterium glutamicum* | Cr | -0.321161 | 0.024445 | negtive |
| *Yersinia rohdei* | CRP | -0.321421 | 0.025904 | negtive |
| *Fluviicola taffensis* | PRO | -0.321591 | 0.024243 | negtive |
| *Caldicellulosiruptor hydrothermalis* | PRO | -0.321609 | 0.024235 | negtive |
| *Candidatus Nanopelagicus abundans* | Cr | -0.32167 | 0.024207 | negtive |
| *Rhodoferax ferrireducens* | BUN | -0.322059 | 0.024026 | negtive |
| *Pseudopedobacter saltans* | BUN | -0.322413 | 0.023863 | negtive |
| *Phycisphaera mikurensis* | UA | -0.322414 | 0.023862 | negtive |
| *Desulfovibrio salexigens* | Cr | -0.322437 | 0.023851 | negtive |
| *Ruminococcus bicirculans* | CysC | -0.322558 | 0.025353 | negtive |
| *butyrate-producing bacterium SM4/1* | UA | -0.322686 | 0.023737 | negtive |
| *Ottowia sp. oral taxon 894* | BUN | -0.322716 | 0.023723 | negtive |
| *Planococcus sp. PAMC 21323* | CRP | -0.322756 | 0.025259 | negtive |
| *Candidatus Pelagibacter ubique* | CysC | -0.322783 | 0.025245 | negtive |
| *Phreatobacter cathodiphilus* | UA | -0.322825 | 0.023674 | negtive |
| *Salinicola tamaricis* | PRO | -0.322868 | 0.023654 | negtive |
| *Bifidobacterium adolescentis* | CRP | -0.323456 | 0.024925 | negtive |
| *endosymbiont of unidentified scaly snail isolate Monju* | CRP | -0.323677 | 0.024821 | negtive |
| *Yersinia rohdei* | PRO | -0.323727 | 0.023264 | negtive |
| *Bacillus sp. OxB-1* | CRP | -0.323873 | 0.024729 | negtive |
| *[Eubacterium] siraeum* | Cr | -0.323986 | 0.023147 | negtive |
| *Dialister sp. Marseille-P5638* | BUN | -0.324395 | 0.022965 | negtive |
| *Jiangella sp. DSM 45060* | Cr | -0.324483 | 0.022925 | negtive |
| *Cutibacterium acnes* | CRP | -0.32472 | 0.024333 | negtive |
| *Agrococcus carbonis* | CysC | -0.324763 | 0.024313 | negtive |
| *Stenotrophomonas sp. LM091* | PRO | -0.324972 | 0.022708 | negtive |
| *Agromyces sp. 30A* | BUN | -0.325256 | 0.022583 | negtive |
| *Pseudopedobacter saltans* | PRO | -0.32531 | 0.02256 | negtive |
| *Halomonas sp. HG01* | PRO | -0.325326 | 0.022553 | negtive |
| *Agrococcus carbonis* | UA | -0.325577 | 0.022442 | negtive |
| *Acidovorax sp. RAC01* | Cr | -0.325589 | 0.022437 | negtive |
| *Mucilaginibacter mallensis* | PRO | -0.325848 | 0.022324 | negtive |
| *Bifidobacterium adolescentis* | ALP | -0.325968 | 0.022272 | negtive |
| *Arthrobacter sp. YC-RL1* | Cr | -0.326153 | 0.022192 | negtive |
| *Polaribacter sp. KT25b* | Cr | -0.326194 | 0.022174 | negtive |
| *Micromonospora viridifaciens* | BUN | -0.326211 | 0.022167 | negtive |
| *Sinomonas sp. R1AF57* | Cr | -0.326282 | 0.022136 | negtive |
| *Deinococcus proteolyticus* | CysC | -0.326698 | 0.02343 | negtive |
| *Caulobacter mirabilis* | PRO | -0.326735 | 0.021941 | negtive |
| *Parvimonas micra* | Cr | -0.327099 | 0.021785 | negtive |
| *Olsenella umbonata* | Cr | -0.327252 | 0.02172 | negtive |
| *Agrococcus jejuensis* | Cr | -0.327264 | 0.021715 | negtive |
| *Hymenobacter sp. APR13* | CysC | -0.327396 | 0.023118 | negtive |
| *Bifidobacterium scardovii* | CysC | -0.327986 | 0.022857 | negtive |
| *Gramella sp. MAR_2010_147* | Cr | -0.328227 | 0.021309 | negtive |
| *Candidatus Symbiobacter mobilis* | UA | -0.328279 | 0.021287 | negtive |
| *Vibrio crassostreae* | Cr | -0.328283 | 0.021285 | negtive |
| *Fluviicola taffensis* | CRP | -0.328301 | 0.022719 | negtive |
| *Bifidobacterium pseudocatenulatum* | PRO | -0.328939 | 0.021012 | negtive |
| *Streptococcus himalayensis* | CRP | -0.328948 | 0.022437 | negtive |
| *Bifidobacterium angulatum* | UA | -0.329225 | 0.020894 | negtive |
| *[Ruminococcus] torques* | CysC | -0.329235 | 0.022313 | negtive |
| *Rufibacter tibetensis* | CysC | -0.329359 | 0.022259 | negtive |
| *Xanthomonas oryzae* | CysC | -0.329414 | 0.022236 | negtive |
| *Clostridiales bacterium CoAT_53-4c* | BUN | -0.329469 | 0.020794 | negtive |
| *Tessaracoccus sp. T2.5-30* | Cr | -0.329513 | 0.020776 | negtive |
| *Myroides odoratimimus* | CysC | -0.330139 | 0.021926 | negtive |
| *Klebsiella phage K5-4* | CysC | -0.330351 | 0.021836 | negtive |
| *Agrococcus jejuensis* | CysC | -0.330386 | 0.021821 | negtive |
| *Mucilaginibacter sp. PAMC 26640* | Cr | -0.330858 | 0.020231 | negtive |
| *Labrenzia sp. VG12* | PRO | -0.331091 | 0.020138 | negtive |
| *Slackia heliotrinireducens* | BUN | -0.331182 | 0.020102 | negtive |
| *Faecalibacterium sp.* | UA | -0.331216 | 0.020088 | negtive |
| *Erysipelotrichaceae bacterium I46* | WBC | -0.331282 | 0.020062 | negtive |
| *Gallibacterium anatis* | CRP | -0.331286 | 0.021443 | negtive |
| *Grimontia hollisae* | PRO | -0.331407 | 0.020012 | negtive |
| *Paludisphaera borealis* | UA | -0.331591 | 0.019939 | negtive |
| *Bifidobacterium bifidum* | PRO | -0.331688 | 0.019901 | negtive |
| *Brachyspira pilosicoli* | BUN | -0.3321 | 0.019738 | negtive |
| *Enterococcus sp. CR-Ec1* | ALB | -0.332379 | 0.019629 | negtive |
| *Sinomonas sp. R1AF57* | CysC | -0.332464 | 0.020957 | negtive |
| *Comamonas kerstersii* | UA | -0.332466 | 0.019595 | negtive |
| *Halomonas sp. 1513* | PRO | -0.332606 | 0.019541 | negtive |
| *Actinomyces radingae* | BUN | -0.332661 | 0.019519 | negtive |
| *Enterobacter cloacae complex sp.* | CysC | -0.332672 | 0.020872 | negtive |
| *Streptomyces glaucescens* | Cr | -0.333055 | 0.019367 | negtive |
| *Gordonia iterans* | Cr | -0.33307 | 0.019361 | negtive |
| *Chromohalobacter salexigens* | PRO | -0.333403 | 0.019233 | negtive |
| *Dokdonia sp. 4H-3-7-5* | UA | -0.333477 | 0.019204 | negtive |
| *Libanicoccus massiliensis* | ALP | -0.333622 | 0.019149 | negtive |
| *Libanicoccus massiliensis* | PRO | -0.333898 | 0.019043 | negtive |
| *Candidatus Nanopelagicus abundans* | CRP | -0.334022 | 0.020327 | negtive |
| *Aeromonas sp. CU5* | UA | -0.334068 | 0.018979 | negtive |
| *Synechococcus sp. JA-2-3B'a(2-13)* | UA | -0.334429 | 0.018842 | negtive |
| *Stenotrophomonas acidaminiphila* | UA | -0.334478 | 0.018824 | negtive |
| *Faecalibacterium phage FP_oengus* | PRO | -0.334545 | 0.018798 | negtive |
| *Candidatus Arthromitus sp. SFB-rat-Yit* | CRP | -0.334829 | 0.020008 | negtive |
| *Vibrio crassostreae* | PRO | -0.334925 | 0.018656 | negtive |
| *Stenotrophomonas sp. LM091* | CRP | -0.335247 | 0.019844 | negtive |
| *Pseudopedobacter saltans* | Cr | -0.335528 | 0.018431 | negtive |
| *[Eubacterium] sulci* | PRO | -0.335677 | 0.018376 | negtive |
| *Agrococcus jejuensis* | BUN | -0.335782 | 0.018337 | negtive |
| *Streptococcus himalayensis* | ALP | -0.335817 | 0.018325 | negtive |
| *Micromonospora auratinigra* | Cr | -0.335891 | 0.018297 | negtive |
| *Dictyoglomus turgidum* | Cr | -0.335964 | 0.018271 | negtive |
| *Bifidobacterium angulatum* | CRP | -0.335971 | 0.019562 | negtive |
| *Clostridiales bacterium CoAT_53-4c* | Cr | -0.336097 | 0.018221 | negtive |
| *Thermoplasmatales archaeon BRNA1* | CysC | -0.336172 | 0.019485 | negtive |
| *Fusobacterium gonidiaformans* | PRO | -0.336629 | 0.018028 | negtive |
| *Fusobacterium gonidiaformans* | BUN | -0.336931 | 0.017918 | negtive |
| *Virgibacillus phasianinus* | PRO | -0.336965 | 0.017905 | negtive |
| *Pasteurellaceae bacterium NI1060* | Cr | -0.337008 | 0.01789 | negtive |
| *Labrenzia sp. VG12* | UA | -0.337017 | 0.017887 | negtive |
| *Corynebacterium humireducens* | Cr | -0.337259 | 0.0178 | negtive |
| *Ottowia sp. oral taxon 894* | PRO | -0.337299 | 0.017785 | negtive |
| *Deinococcus proteolyticus* | PRO | -0.337352 | 0.017766 | negtive |
| *Prevotella jejuni* | UA | -0.337381 | 0.017756 | negtive |
| *Hymenobacter sp. PAMC 26628* | BUN | -0.337505 | 0.017712 | negtive |
| *Muricauda ruestringensis* | BUN | -0.337548 | 0.017696 | negtive |
| *Stigmatella aurantiaca* | CysC | -0.337753 | 0.018885 | negtive |
| *Actinomyces sp. Chiba101* | CysC | -0.337866 | 0.018843 | negtive |
| *Arenibacter algicola* | PRO | -0.337909 | 0.017567 | negtive |
| *Tannerella forsythia* | PRO | -0.337994 | 0.017537 | negtive |
| *Vibrio tritonius* | PRO | -0.338296 | 0.01743 | negtive |
| *Desulfovibrio salexigens* | PRO | -0.338323 | 0.01742 | negtive |
| *Caulobacter mirabilis* | BUN | -0.338396 | 0.017395 | negtive |
| *Kiritimatiella glycovorans* | ALP | -0.338592 | 0.017326 | negtive |
| *Olsenella sp. oral taxon 807* | BUN | -0.338632 | 0.017312 | negtive |
| *Plantactinospora sp. KBS50* | UA | -0.338714 | 0.017283 | negtive |
| *Xylella fastidiosa* | PRO | -0.338748 | 0.017271 | negtive |
| *Fastidiosipila sanguinis* | PRO | -0.339072 | 0.017158 | negtive |
| *Lachnoclostridium phocaeense* | UA | -0.339269 | 0.017089 | negtive |
| *Jiangella sp. DSM 45060* | CRP | -0.339276 | 0.018322 | negtive |
| *Paenibacillus mucilaginosus* | CRP | -0.339333 | 0.018301 | negtive |
| *Geobacter uraniireducens* | UA | -0.339698 | 0.016941 | negtive |
| *Halobacillus mangrovi* | BUN | -0.339724 | 0.016932 | negtive |
| *Desulfotalea psychrophila* | CysC | -0.339792 | 0.018134 | negtive |
| *[Mycobacterium] stephanolepidis* | ALB | -0.339793 | 0.016908 | negtive |
| *Fermentimonas caenicola* | CysC | -0.340047 | 0.018042 | negtive |
| *Salmonella bongori* | CRP | -0.340279 | 0.017958 | negtive |
| *Coprococcus sp. ART55/1* | CRP | -0.340366 | 0.017927 | negtive |
| *Xanthomonas oryzae* | UA | -0.340441 | 0.016686 | negtive |
| *[Clostridium] sphenoides* | HGB | -0.340504 | 0.016665 | negtive |
| *Gramella sp. MAR_2010_147* | CysC | -0.34077 | 0.017783 | negtive |
| *Gramella sp. MAR_2010_147* | PRO | -0.341224 | 0.016422 | negtive |
| *Agrobacterium rhizogenes* | PRO | -0.34156 | 0.016309 | negtive |
| *Methylobacterium nodulans* | CysC | -0.341942 | 0.017369 | negtive |
| *butyrate-producing bacterium SS3/4* | PRO | -0.341983 | 0.016168 | negtive |
| *Romboutsia ilealis* | Cr | -0.342 | 0.016163 | negtive |
| *Pseudopedobacter saltans* | UA | -0.342263 | 0.016076 | negtive |
| *Polaribacter vadi* | CysC | -0.342304 | 0.017243 | negtive |
| *Prevotella melaninogenica* | CysC | -0.342317 | 0.017239 | negtive |
| *Syntrophus aciditrophicus* | PRO | -0.342535 | 0.015986 | negtive |
| *Mycobacterium sp. djl-10* | CRP | -0.342692 | 0.017109 | negtive |
| *Sulfurifustis variabilis* | PRO | -0.342918 | 0.015861 | negtive |
| *Caulobacter mirabilis* | Cr | -0.343298 | 0.015738 | negtive |
| *Ruegeria sp. TM1040* | UA | -0.343345 | 0.015722 | negtive |
| *Micromonospora auratinigra* | CysC | -0.343465 | 0.016845 | negtive |
| *Azotobacter chroococcum* | CRP | -0.343465 | 0.016845 | negtive |
| *Pelobacter carbinolicus* | UA | -0.343658 | 0.015622 | negtive |
| *Cellulomonas fimi* | Cr | -0.34377 | 0.015585 | negtive |
| *Mahella australiensis* | CysC | -0.344055 | 0.016645 | negtive |
| *Methylobacterium nodulans* | Cr | -0.34419 | 0.015451 | negtive |
| *Roseburia hominis* | PRO | -0.3443 | 0.015416 | negtive |
| *Paenibacillus alvei* | BUN | -0.344675 | 0.015297 | negtive |
| *Synechococcus sp. JA-2-3B'a(2-13)* | PRO | -0.344877 | 0.015233 | negtive |
| *Bifidobacterium scardovii* | Cr | -0.345215 | 0.015127 | negtive |
| *Blautia obeum* | UA | -0.345392 | 0.015072 | negtive |
| *Mucilaginibacter gotjawali* | BUN | -0.345498 | 0.015039 | negtive |
| *[Ruminococcus] torques* | Cr | -0.345878 | 0.014921 | negtive |
| *Paenibacillus alvei* | PRO | -0.345955 | 0.014897 | negtive |
| *Bifidobacterium adolescentis* | BUN | -0.34598 | 0.014889 | negtive |
| *Prosthecochloris aestuarii* | CRP | -0.346053 | 0.015984 | negtive |
| *Faecalibacterium phage FP_Taranis* | CysC | -0.346235 | 0.015925 | negtive |
| *[Clostridium] sphenoides* | ALB | -0.346337 | 0.014779 | negtive |
| *Oceanithermus profundus* | PRO | -0.346618 | 0.014693 | negtive |
| *Mucilaginibacter sp. PAMC 26640* | PRO | -0.346653 | 0.014682 | negtive |
| *Mycobacterium sp. djl-10* | UA | -0.346676 | 0.014675 | negtive |
| *Bifidobacterium bifidum* | CRP | -0.346945 | 0.015696 | negtive |
| *Gemmatirosa kalamazoonesis* | CysC | -0.346978 | 0.015685 | negtive |
| *Mycolicibacterium rutilum* | Cr | -0.347047 | 0.014563 | negtive |
| *Vibrio owensii* | UA | -0.347049 | 0.014562 | negtive |
| *Geobacillus sp. WCH70* | PRO | -0.347112 | 0.014543 | negtive |
| *Caldithrix abyssi* | HGB | -0.347351 | 0.014471 | negtive |
| *Arthrobacter sp. YC-RL1* | UA | -0.347498 | 0.014426 | negtive |
| *Agromyces sp. 30A* | CysC | -0.347978 | 0.015368 | negtive |
| *Xanthomonas oryzae* | Cr | -0.348026 | 0.014268 | negtive |
| *Paenibacillus kribbensis* | PRO | -0.348289 | 0.01419 | negtive |
| *Stigmatella aurantiaca* | CRP | -0.348835 | 0.0151 | negtive |
| *Streptomyces sp. SF2575* | HGB | -0.348903 | 0.014009 | negtive |
| *[Eubacterium] sulci* | CysC | -0.349754 | 0.014818 | negtive |
| *Pseudoalteromonas piscicida* | PRO | -0.349833 | 0.013739 | negtive |
| *Bordetella bronchiseptica* | UA | -0.350074 | 0.013669 | negtive |
| *Corynebacterium uterequi* | CRP | -0.350082 | 0.014718 | negtive |
| *Bifidobacterium angulatum* | BUN | -0.350101 | 0.013662 | negtive |
| *Kyrpidia sp. EA-1* | PRO | -0.350333 | 0.013595 | negtive |
| *Alkalitalea saponilacus* | BUN | -0.350353 | 0.013589 | negtive |
| *Thermoanaerobacterium saccharolyticum* | Cr | -0.350525 | 0.01354 | negtive |
| *Tannerella forsythia* | UA | -0.350801 | 0.013462 | negtive |
| *Hymenobacter sp. APR13* | UA | -0.350844 | 0.01345 | negtive |
| *Clostridiales bacterium CoAT_53-4c* | PRO | -0.351006 | 0.013404 | negtive |
| *Corynebacterium glutamicum* | CysC | -0.351115 | 0.014408 | negtive |
| *Paraburkholderia sprentiae* | PRO | -0.35181 | 0.013178 | negtive |
| *Gemmatirosa kalamazoonesis* | Cr | -0.35193 | 0.013145 | negtive |
| *Corynebacterium imitans* | PRO | -0.352224 | 0.013064 | negtive |
| *Grimontia hollisae* | ALP | -0.352394 | 0.013017 | negtive |
| *Mucilaginibacter sp. PAMC 26640* | BUN | -0.352395 | 0.013017 | negtive |
| *Phoenicibacter massiliensis* | CysC | -0.352469 | 0.014009 | negtive |
| *Mucilaginibacter gotjawali* | UA | -0.352646 | 0.012948 | negtive |
| *Lachnoclostridium sp. YL32* | HGB | -0.352752 | 0.012919 | negtive |
| *Mahella australiensis* | BUN | -0.352776 | 0.012912 | negtive |
| *Phoenicibacter massiliensis* | UA | -0.352791 | 0.012908 | negtive |
| *Alkalitalea saponilacus* | Cr | -0.352905 | 0.012877 | negtive |
| *Aspergillus aculeatus* | BUN | -0.35302 | 0.012845 | negtive |
| *Sneathia amnii* | BUN | -0.353305 | 0.012768 | negtive |
| *Synechococcus sp. SynAce01* | PRO | -0.353334 | 0.01276 | negtive |
| *Chromohalobacter salexigens* | CRP | -0.353356 | 0.013753 | negtive |
| *Olsenella sp. oral taxon 807* | CRP | -0.353361 | 0.013752 | negtive |
| *Trichomonas vaginalis* | Cr | -0.353481 | 0.01272 | negtive |
| *Corynebacterium humireducens* | CRP | -0.353843 | 0.013615 | negtive |
| *Hymenobacter sp. PAMC 26628* | PRO | -0.353914 | 0.012604 | negtive |
| *Anaerostipes hadrus* | PRO | -0.354325 | 0.012494 | negtive |
| *Corynebacterium sphenisci* | UA | -0.354623 | 0.012415 | negtive |
| *Thermotoga sp. RQ7* | Cr | -0.354801 | 0.012368 | negtive |
| *[Clostridium] saccharolyticum* | UA | -0.354832 | 0.01236 | negtive |
| *Agrobacterium rhizogenes* | CRP | -0.35513 | 0.013254 | negtive |
| *Pseudomonas fulva* | Cr | -0.355234 | 0.012254 | negtive |
| *Lactobacillus terrae* | PRO | -0.355464 | 0.012195 | negtive |
| *Bifidobacterium catenulatum* | CysC | -0.355563 | 0.013134 | negtive |
| *Paenibacillus mucilaginosus* | PRO | -0.355565 | 0.012168 | negtive |
| *Pontibacter akesuensis* | UA | -0.355647 | 0.012147 | negtive |
| *Bacillus oceanisediminis* | Cr | -0.355685 | 0.012137 | negtive |
| *Acidovorax carolinensis* | CRP | -0.355913 | 0.013038 | negtive |
| *Pectobacterium polaris* | PRO | -0.355937 | 0.012072 | negtive |
| *Bradyrhizobium sp. BTAi1* | UA | -0.356848 | 0.011839 | negtive |
| *Bifidobacterium breve* | ALP | -0.357198 | 0.01175 | negtive |
| *Barnesiella viscericola* | PRO | -0.357236 | 0.01174 | negtive |
| *Mycolicibacterium thermoresistibile* | CysC | -0.357627 | 0.012577 | negtive |
| *Brevundimonas naejangsanensis* | CysC | -0.357783 | 0.012535 | negtive |
| *Acutalibacter muris* | CysC | -0.358579 | 0.012326 | negtive |
| *Pseudomonas guangdongensis* | CRP | -0.358607 | 0.012319 | negtive |
| *Paenibacillus mucilaginosus* | UA | -0.358608 | 0.011399 | negtive |
| *Aureitalea sp. RR4-38* | CysC | -0.358807 | 0.012267 | negtive |
| *[Eubacterium] minutum* | ALP | -0.358932 | 0.011319 | negtive |
| *Candidatus Pelagibacter ubique* | Cr | -0.359047 | 0.011291 | negtive |
| *Conexibacter woesei* | PRO | -0.359192 | 0.011256 | negtive |
| *Acidovorax carolinensis* | Cr | -0.359431 | 0.011198 | negtive |
| *Verminephrobacter eiseniae* | PRO | -0.359686 | 0.011136 | negtive |
| *Coprococcus catus* | BUN | -0.359707 | 0.011131 | negtive |
| *Caldicellulosiruptor hydrothermalis* | CRP | -0.359722 | 0.012031 | negtive |
| *Frondihabitans sp. 762G35* | Cr | -0.359924 | 0.011079 | negtive |
| *Desulfitobacterium metallireducens* | PRO | -0.360146 | 0.011026 | negtive |
| *Sulfurifustis variabilis* | Cr | -0.360313 | 0.010986 | negtive |
| *Frondihabitans sp. 762G35* | PRO | -0.360346 | 0.010978 | negtive |
| *Cutibacterium acnes* | PRO | -0.360384 | 0.010969 | negtive |
| *Enterobacter cloacae complex sp.* | UA | -0.360552 | 0.010929 | negtive |
| *Thermoanaerobacter kivui* | PRO | -0.360739 | 0.010885 | negtive |
| *Planococcus sp. PAMC 21323* | CysC | -0.361193 | 0.011661 | negtive |
| *Gloeobacter violaceus* | PRO | -0.361564 | 0.010692 | negtive |
| *Corynebacterium glutamicum* | BUN | -0.361673 | 0.010666 | negtive |
| *Comamonas kerstersii* | PRO | -0.362022 | 0.010586 | negtive |
| *Rhodoferax ferrireducens* | PRO | -0.362595 | 0.010454 | negtive |
| *Francisella philomiragia* | Cr | -0.362712 | 0.010428 | negtive |
| *Aeromonas sp. CU5* | CRP | -0.362863 | 0.011252 | negtive |
| *Prevotella fusca* | UA | -0.362945 | 0.010375 | negtive |
| *[Eubacterium] minutum* | UA | -0.362996 | 0.010363 | negtive |
| *[Eubacterium] siraeum* | BUN | -0.363075 | 0.010345 | negtive |
| *Methylosinus trichosporium* | PRO | -0.3632 | 0.010317 | negtive |
| *Mogibacterium pumilum* | CRP | -0.363311 | 0.011145 | negtive |
| *Brachybacterium sp. VM2412* | Cr | -0.363334 | 0.010287 | negtive |
| *Stenotrophomonas acidaminiphila* | CRP | -0.363354 | 0.011134 | negtive |
| *[Mycobacterium] stephanolepidis* | WBC | -0.363497 | 0.01025 | negtive |
| *Chitinophaga pinensis* | UA | -0.363558 | 0.010237 | negtive |
| *Actinobacillus suis* | Cr | -0.364054 | 0.010126 | negtive |
| *Rhodococcus virus Pepy6* | HGB | -0.364082 | 0.01012 | negtive |
| *Slackia heliotrinireducens* | CysC | -0.36414 | 0.010948 | negtive |
| *Actinomyces succiniciruminis* | CysC | -0.364248 | 0.010923 | negtive |
| *Polaribacter sp. KT25b* | PRO | -0.364293 | 0.010073 | negtive |
| *Mycobacterium sp. djl-10* | PRO | -0.364297 | 0.010072 | negtive |
| *Thauera aromatica* | UA | -0.36431 | 0.010069 | negtive |
| *Brachybacterium sp. VM2412* | CysC | -0.364527 | 0.010857 | negtive |
| *Faecalibacterium phage FP_oengus* | BUN | -0.364606 | 0.010004 | negtive |
| *Bifidobacterium angulatum* | Cr | -0.364852 | 0.00995 | negtive |
| *Corynebacterium uterequi* | Cr | -0.365158 | 0.009883 | negtive |
| *Prevotella fusca* | Cr | -0.365524 | 0.009804 | negtive |
| *Pasteurellaceae bacterium NI1060* | BUN | -0.365789 | 0.009747 | negtive |
| *Alkalitalea saponilacus* | CysC | -0.365887 | 0.010543 | negtive |
| *Dictyoglomus turgidum* | CysC | -0.365889 | 0.010543 | negtive |
| *Francisella philomiragia* | CysC | -0.365889 | 0.010543 | negtive |
| *Faecalibacterium phage FP_oengus* | UA | -0.366058 | 0.009689 | negtive |
| *Brevundimonas naejangsanensis* | CRP | -0.36609 | 0.010497 | negtive |
| *Agrobacterium rhizogenes* | UA | -0.366564 | 0.009582 | negtive |
| *Corynebacterium glyciniphilum* | CysC | -0.366571 | 0.010388 | negtive |
| *[Ruminococcus] torques* | BUN | -0.366749 | 0.009543 | negtive |
| *Pelobacter carbinolicus* | CRP | -0.367062 | 0.010278 | negtive |
| *Serratia marcescens* | CRP | -0.367062 | 0.010278 | negtive |
| *Candidatus Pelagibacter ubique* | BUN | -0.367274 | 0.009432 | negtive |
| *Stigmatella aurantiaca* | BUN | -0.36753 | 0.009379 | negtive |
| *Pasteurellaceae bacterium NI1060* | CysC | -0.368917 | 0.009872 | negtive |
| *Desulfotalea psychrophila* | CRP | -0.368945 | 0.009866 | negtive |
| *Synechococcus sp. JA-2-3B'a(2-13)* | Cr | -0.368967 | 0.009085 | negtive |
| *[Eubacterium] sulci* | BUN | -0.369045 | 0.009069 | negtive |
| *Sinomonas sp. R1AF57* | UA | -0.369207 | 0.009036 | negtive |
| *Propionibacterium freudenreichii* | UA | -0.369272 | 0.009023 | negtive |
| *Faecalibacterium phage FP_Taranis* | BUN | -0.369514 | 0.008975 | negtive |
| *Thermoplasmatales archaeon BRNA1* | Cr | -0.370005 | 0.008877 | negtive |
| *Prevotella fusca* | BUN | -0.370423 | 0.008794 | negtive |
| *Thermoplasmatales archaeon BRNA1* | BUN | -0.370518 | 0.008776 | negtive |
| *Olsenella umbonata* | CRP | -0.370542 | 0.009527 | negtive |
| *Propionibacterium freudenreichii* | BUN | -0.370678 | 0.008744 | negtive |
| *Coprococcus catus* | CysC | -0.370817 | 0.00947 | negtive |
| *Pseudomonas fulva* | BUN | -0.371051 | 0.008672 | negtive |
| *Actinomyces radingae* | CysC | -0.371088 | 0.009414 | negtive |
| *Rufibacter tibetensis* | BUN | -0.371112 | 0.00866 | negtive |
| *Devosia sp. H5989* | Cr | -0.371255 | 0.008632 | negtive |
| *Pseudomonas putida* | PRO | -0.371573 | 0.008571 | negtive |
| *Burkholderia ubonensis* | UA | -0.371714 | 0.008544 | negtive |
| *Actinobacillus suis* | BUN | -0.372678 | 0.008361 | negtive |
| *Bifidobacterium angulatum* | CysC | -0.372757 | 0.009074 | negtive |
| *Salmonella bongori* | Cr | -0.373012 | 0.008298 | negtive |
| *Sterolibacterium denitrificans* | CRP | -0.373012 | 0.009023 | negtive |
| *Prevotella dentalis* | CysC | -0.373911 | 0.008846 | negtive |
| *Muricauda ruestringensis* | CysC | -0.374066 | 0.008815 | negtive |
| *Methanocorpusculum labreanum* | CysC | -0.374317 | 0.008766 | negtive |
| *Bacillus oceanisediminis* | CRP | -0.374576 | 0.008716 | negtive |
| *Martelella mediterranea* | BUN | -0.374632 | 0.008 | negtive |
| *Polaribacter vadi* | Cr | -0.374929 | 0.007946 | negtive |
| *Devosia sp. H5989* | CysC | -0.374929 | 0.008648 | negtive |
| *Planococcus sp. MB-3u-03* | CRP | -0.374987 | 0.008637 | negtive |
| *Sinomonas sp. R1AF57* | PRO | -0.375194 | 0.007899 | negtive |
| *Corynebacterium glutamicum* | UA | -0.375278 | 0.007884 | negtive |
| *Devosia sp. H5989* | BUN | -0.375453 | 0.007852 | negtive |
| *Bifidobacterium choerinum* | UA | -0.375536 | 0.007838 | negtive |
| *Acutalibacter muris* | BUN | -0.376011 | 0.007753 | negtive |
| *Faecalibacterium phage FP_Toutatis* | PRO | -0.376383 | 0.007688 | negtive |
| *Caldicellulosiruptor bescii* | PRO | -0.376508 | 0.007666 | negtive |
| *Acinetobacter sp. TGL-Y2* | BUN | -0.376663 | 0.007639 | negtive |
| *Mycolicibacterium thermoresistibile* | BUN | -0.377049 | 0.007572 | negtive |
| *Ruminococcus bicirculans* | BUN | -0.377261 | 0.007536 | negtive |
| *Desulfobacula toluolica* | UA | -0.377493 | 0.007496 | negtive |
| *Gemmatirosa kalamazoonesis* | BUN | -0.378014 | 0.007407 | negtive |
| *Tannerella sp. oral taxon HOT-286* | UA | -0.378253 | 0.007367 | negtive |
| *Thermincola potens* | PRO | -0.378687 | 0.007294 | negtive |
| *Xanthomonas oryzae* | BUN | -0.378978 | 0.007246 | negtive |
| *Bifidobacterium dentium* | PRO | -0.378989 | 0.007244 | negtive |
| *Bifidobacterium dentium* | BUN | -0.379241 | 0.007202 | negtive |
| *Myxococcus stipitatus* | CRP | -0.379448 | 0.007817 | negtive |
| *Candidatus Symbiobacter mobilis* | PRO | -0.379807 | 0.007109 | negtive |
| *Lysinibacillus fusiformis* | CRP | -0.380542 | 0.007626 | negtive |
| *Halobacillus mangrovi* | PRO | -0.380822 | 0.006945 | negtive |
| *Francisella philomiragia* | PRO | -0.381236 | 0.006879 | negtive |
| *Mahella australiensis* | PRO | -0.381473 | 0.006841 | negtive |
| *Phoenicibacter massiliensis* | BUN | -0.381956 | 0.006765 | negtive |
| *Photobacterium gaetbulicola* | UA | -0.382036 | 0.006753 | negtive |
| *Geobacter daltonii* | CRP | -0.382056 | 0.007369 | negtive |
| *Pseudomonas putida* | UA | -0.382233 | 0.006722 | negtive |
| *Luteitalea pratensis* | PRO | -0.382281 | 0.006714 | negtive |
| *Mordavella sp. Marseille-P3756* | PRO | -0.382298 | 0.006712 | negtive |
| *Blautia obeum* | CRP | -0.382558 | 0.007286 | negtive |
| *Caldicellulosiruptor bescii* | CysC | -0.382755 | 0.007253 | negtive |
| *Hymenobacter sp. PAMC 26554* | BUN | -0.383065 | 0.006593 | negtive |
| *Bradyrhizobium sp. BTAi1* | PRO | -0.383106 | 0.006587 | negtive |
| *Sneathia amnii* | PRO | -0.383195 | 0.006574 | negtive |
| *Hymenobacter sedentarius* | PRO | -0.383447 | 0.006535 | negtive |
| *Aeromonas sp. ASNIH4* | ALP | -0.383598 | 0.006512 | negtive |
| *Hymenobacter sp. PAMC 26628* | Cr | -0.383881 | 0.006469 | negtive |
| *Caldicellulosiruptor bescii* | Cr | -0.38404 | 0.006446 | negtive |
| *Moorella thermoacetica* | UA | -0.38407 | 0.006441 | negtive |
| *Brevundimonas naejangsanensis* | UA | -0.384189 | 0.006423 | negtive |
| *Corynebacterium glyciniphilum* | BUN | -0.384639 | 0.006356 | negtive |
| *Prevotella ruminicola* | UA | -0.384682 | 0.00635 | negtive |
| *Coprococcus catus* | Cr | -0.384712 | 0.006345 | negtive |
| *Dialister sp. Marseille-P5638* | Cr | -0.384712 | 0.006345 | negtive |
| *Blautia obeum* | PRO | -0.384886 | 0.00632 | negtive |
| *Micromonospora auratinigra* | CRP | -0.384906 | 0.006906 | negtive |
| *Rufibacter tibetensis* | Cr | -0.385107 | 0.006287 | negtive |
| *Rhodoferax koreense* | PRO | -0.38514 | 0.006282 | negtive |
| *Pseudomonas fulva* | UA | -0.385405 | 0.006243 | negtive |
| *Slackia heliotrinireducens* | PRO | -0.386395 | 0.0061 | negtive |
| *Rufibacter tibetensis* | UA | -0.386482 | 0.006088 | negtive |
| *Actinomyces succiniciruminis* | Cr | -0.386549 | 0.006078 | negtive |
| *Candidatus Nanopelagicus abundans* | PRO | -0.386869 | 0.006033 | negtive |
| *Plesiomonas shigelloides* | PRO | -0.387051 | 0.006007 | negtive |
| *Planococcus sp. PAMC 21323* | UA | -0.387511 | 0.005942 | negtive |
| *Acidithiobacillus caldus* | UA | -0.387982 | 0.005877 | negtive |
| *Rhodococcus opacus* | PRO | -0.388063 | 0.005866 | negtive |
| *Frondihabitans sp. 762G35* | CRP | -0.388133 | 0.006412 | negtive |
| *Anaerolinea thermophila* | PRO | -0.388329 | 0.005829 | negtive |
| *Vibrio campbellii* | Cr | -0.388412 | 0.005817 | negtive |
| *Rhodoferax ferrireducens* | UA | -0.388707 | 0.005777 | negtive |
| *Ketobacter alkanivorans* | Cr | -0.389159 | 0.005716 | negtive |
| *Kluyvera georgiana* | PRO | -0.389249 | 0.005704 | negtive |
| *Acidovorax avenae* | PRO | -0.389251 | 0.005703 | negtive |
| *Mucilaginibacter sp. PAMC 26640* | CysC | -0.38937 | 0.006231 | negtive |
| *Prevotella jejuni* | Cr | -0.389508 | 0.005669 | negtive |
| *Martelella mediterranea* | CysC | -0.389513 | 0.00621 | negtive |
| *Faecalibacterium sp.* | PRO | -0.3898 | 0.00563 | negtive |
| *Variovorax sp. PAMC 28711* | UA | -0.390159 | 0.005582 | negtive |
| *Faecalibacterium phage FP_oengus* | CysC | -0.390251 | 0.006104 | negtive |
| *Brevundimonas naejangsanensis* | Cr | -0.390691 | 0.005512 | negtive |
| *Prevotella ruminicola* | Cr | -0.390886 | 0.005486 | negtive |
| *Faecalibacterium phage FP_Lugh* | BUN | -0.390911 | 0.005483 | negtive |
| *Brachyspira pilosicoli* | CRP | -0.391095 | 0.005985 | negtive |
| *Novosphingobium resinovorum* | UA | -0.391362 | 0.005425 | negtive |
| *Corynebacterium glyciniphilum* | PRO | -0.39156 | 0.005399 | negtive |
| *Trichomonas vaginalis* | PRO | -0.391834 | 0.005364 | negtive |
| *Ketobacter alkanivorans* | PRO | -0.391938 | 0.005351 | negtive |
| *Faecalibacterium phage FP_Taranis* | Cr | -0.392019 | 0.00534 | negtive |
| *Brachyspira pilosicoli* | CysC | -0.392042 | 0.005854 | negtive |
| *Microbacterium aurum* | CRP | -0.392153 | 0.005839 | negtive |
| *Jatrophihabitans sp. GAS493* | CRP | -0.392212 | 0.005831 | negtive |
| *Myroides odoratimimus* | UA | -0.392274 | 0.005308 | negtive |
| *Faecalibacterium prausnitzii* | UA | -0.39254 | 0.005274 | negtive |
| *Dictyoglomus turgidum* | PRO | -0.392838 | 0.005237 | negtive |
| *Methanocorpusculum labreanum* | BUN | -0.393005 | 0.005216 | negtive |
| *Corynebacterium uterequi* | CysC | -0.393225 | 0.005694 | negtive |
| *Dictyoglomus turgidum* | BUN | -0.393226 | 0.005188 | negtive |
| *Rhizobium sp. ACO-34A* | CRP | -0.393302 | 0.005684 | negtive |
| *Thermotoga sp. RQ7* | PRO | -0.393389 | 0.005168 | negtive |
| *Acinetobacter sp. TGL-Y2* | Cr | -0.393604 | 0.005142 | negtive |
| *Propionibacterium freudenreichii* | PRO | -0.39394 | 0.0051 | negtive |
| *Mycolicibacterium thermoresistibile* | CRP | -0.39436 | 0.005544 | negtive |
| *Acetobacter ghanensis* | UA | -0.395112 | 0.004959 | negtive |
| *Faecalibacterium sp.* | BUN | -0.395336 | 0.004932 | negtive |
| *Ruminococcus bicirculans* | PRO | -0.395342 | 0.004931 | negtive |
| *Faecalibacterium sp.* | CysC | -0.39557 | 0.005388 | negtive |
| *Corynebacterium humireducens* | PRO | -0.395809 | 0.004876 | negtive |
| *Fervidobacterium pennivorans* | UA | -0.396208 | 0.004829 | negtive |
| *Acutalibacter muris* | Cr | -0.396413 | 0.004806 | negtive |
| *Phoenicibacter massiliensis* | Cr | -0.396499 | 0.004795 | negtive |
| *Isoptericola dokdonensis* | PRO | -0.396581 | 0.004786 | negtive |
| *Cryobacterium arcticum* | CRP | -0.396584 | 0.005261 | negtive |
| *Deinococcus proteolyticus* | CRP | -0.396746 | 0.00524 | negtive |
| *Sneathia amnii* | Cr | -0.397314 | 0.004702 | negtive |
| *butyrate-producing bacterium SS3/4* | UA | -0.397439 | 0.004688 | negtive |
| *Streptomyces lincolnensis* | CysC | -0.397954 | 0.005092 | negtive |
| *Streptomyces violaceoruber* | ALP | -0.398008 | 0.004623 | negtive |
| *Ottowia sp. oral taxon 894* | Cr | -0.398362 | 0.004584 | negtive |
| *Actinoplanes sp. N902-109* | UA | -0.398415 | 0.004578 | negtive |
| *Barnesiella viscericola* | BUN | -0.398745 | 0.004541 | negtive |
| *Micromonospora viridifaciens* | CysC | -0.398815 | 0.004989 | negtive |
| *Mucilaginibacter gotjawali* | Cr | -0.398973 | 0.004516 | negtive |
| *Aspergillus aculeatus* | CysC | -0.398997 | 0.004967 | negtive |
| *Octadecabacter arcticus* | PRO | -0.3992 | 0.004491 | negtive |
| *Sneathia amnii* | CysC | -0.399207 | 0.004942 | negtive |
| *Bifidobacterium scardovii* | PRO | -0.399222 | 0.004489 | negtive |
| *Micromonospora viridifaciens* | Cr | -0.399254 | 0.004485 | negtive |
| *Corynebacterium frankenforstense* | UA | -0.399418 | 0.004467 | negtive |
| *Agromyces sp. 30A* | Cr | -0.399873 | 0.004418 | negtive |
| *Candidatus Pelagibacter ubique* | PRO | -0.40001 | 0.004403 | negtive |
| *Cryobacterium arcticum* | CysC | -0.400375 | 0.004806 | negtive |
| *Prevotella ruminicola* | PRO | -0.400408 | 0.00436 | negtive |
| *Tessaracoccus sp. T2.5-30* | CysC | -0.401026 | 0.004731 | negtive |
| *Bifidobacterium dentium* | Cr | -0.401042 | 0.004293 | negtive |
| *Bifidobacterium kashiwanohense* | BUN | -0.401143 | 0.004283 | negtive |
| *Caldicellulosiruptor bescii* | BUN | -0.401756 | 0.004219 | negtive |
| *Mucilaginibacter gotjawali* | PRO | -0.401834 | 0.004211 | negtive |
| *Propionibacterium freudenreichii* | Cr | -0.402062 | 0.004187 | negtive |
| *Bifidobacterium dentium* | CRP | -0.402459 | 0.004571 | negtive |
| *Gemmatirosa kalamazoonesis* | PRO | -0.402673 | 0.004125 | negtive |
| *Actinomyces radingae* | Cr | -0.402776 | 0.004114 | negtive |
| *Bifidobacterium adolescentis* | CysC | -0.402899 | 0.004523 | negtive |
| *Pantoea gaviniae* | PRO | -0.404946 | 0.003899 | negtive |
| *Janibacter indicus* | CRP | -0.405045 | 0.004293 | negtive |
| *Achromobacter sp. AONIH1* | UA | -0.405095 | 0.003885 | negtive |
| *Barnesiella viscericola* | Cr | -0.40594 | 0.003804 | negtive |
| *Enterococcus sp. CR-Ec1* | HGB | -0.406786 | 0.003725 | negtive |
| *Mogibacterium pumilum* | PRO | -0.407253 | 0.003682 | negtive |
| *Roseiflexus castenholzii* | UA | -0.407283 | 0.003679 | negtive |
| *Bradyrhizobium sp. BTAi1* | CRP | -0.407358 | 0.004058 | negtive |
| *Verminephrobacter eiseniae* | CRP | -0.407519 | 0.004042 | negtive |
| *Prevotella ruminicola* | BUN | -0.407573 | 0.003652 | negtive |
| *Lactobacillus terrae* | UA | -0.407642 | 0.003646 | negtive |
| *Faecalibacterium sp.* | Cr | -0.407813 | 0.00363 | negtive |
| *Methanocorpusculum labreanum* | CRP | -0.408174 | 0.003977 | negtive |
| *Cryobacterium arcticum* | PRO | -0.40854 | 0.003565 | negtive |
| *Photobacterium gaetbulicola* | PRO | -0.408854 | 0.003537 | negtive |
| *Faecalibacterium phage FP_Lugh* | PRO | -0.408884 | 0.003534 | negtive |
| *Microbacterium sp. XT11* | Cr | -0.409083 | 0.003517 | negtive |
| *Olsenella umbonata* | PRO | -0.409194 | 0.003507 | negtive |
| *Gloeobacter violaceus* | UA | -0.409217 | 0.003505 | negtive |
| *Synechococcus sp. JA-2-3B'a(2-13)* | BUN | -0.409248 | 0.003502 | negtive |
| *Cryobacterium arcticum* | UA | -0.40969 | 0.003463 | negtive |
| *Bifidobacterium kashiwanohense* | Cr | -0.409818 | 0.003452 | negtive |
| *Ruminococcus bicirculans* | Cr | -0.409869 | 0.003448 | negtive |
| *Martelella mediterranea* | CRP | -0.410117 | 0.003791 | negtive |
| *Plantibacter flavus* | CysC | -0.41042 | 0.003763 | negtive |
| *Paenibacillus alvei* | CysC | -0.410514 | 0.003754 | negtive |
| *Microbacterium sp. XT11* | CysC | -0.410557 | 0.00375 | negtive |
| *[Eubacterium] siraeum* | PRO | -0.411296 | 0.003326 | negtive |
| *Cryobacterium arcticum* | BUN | -0.411304 | 0.003325 | negtive |
| *Acidovorax sp. RAC01* | CRP | -0.411373 | 0.003675 | negtive |
| *Mycolicibacterium thermoresistibile* | UA | -0.411793 | 0.003284 | negtive |
| *Actinomyces radingae* | PRO | -0.411888 | 0.003276 | negtive |
| *Deinococcus proteolyticus* | UA | -0.41217 | 0.003253 | negtive |
| *Hymenobacter sp. PAMC 26554* | CysC | -0.412537 | 0.003571 | negtive |
| *Desulfovibrio piger* | UA | -0.413818 | 0.00312 | negtive |
| *Rhodococcus opacus* | CRP | -0.413908 | 0.003451 | negtive |
| *Prevotella jejuni* | BUN | -0.414309 | 0.003081 | negtive |
| *Hymenobacter sedentarius* | CRP | -0.414342 | 0.003413 | negtive |
| *Bifidobacterium adolescentis* | Cr | -0.414972 | 0.003029 | negtive |
| *endosymbiont of unidentified scaly snail isolate Monju* | PRO | -0.415231 | 0.003009 | negtive |
| *Coprococcus catus* | UA | -0.415246 | 0.003008 | negtive |
| *Faecalibacterium prausnitzii* | BUN | -0.415431 | 0.002994 | negtive |
| *Acidovorax carolinensis* | PRO | -0.415966 | 0.002953 | negtive |
| *Enterococcus gallinarum* | ALB | -0.415978 | 0.002952 | negtive |
| *Brachyspira pilosicoli* | Cr | -0.416299 | 0.002928 | negtive |
| *Kluyvera georgiana* | CysC | -0.416479 | 0.003235 | negtive |
| *Streptomyces gilvosporeus* | UA | -0.416546 | 0.002909 | negtive |
| *Bifidobacterium scardovii* | UA | -0.416777 | 0.002892 | negtive |
| *Geobacter metallireducens* | UA | -0.416779 | 0.002892 | negtive |
| *Microbacterium sp. XT11* | BUN | -0.417267 | 0.002856 | negtive |
| *Serratia marcescens* | UA | -0.417287 | 0.002854 | negtive |
| *Dialister sp. Marseille-P5638* | CysC | -0.417338 | 0.003166 | negtive |
| *Corynebacterium frankenforstense* | CRP | -0.418217 | 0.003096 | negtive |
| *Dialister sp. Marseille-P5638* | UA | -0.419073 | 0.002726 | negtive |
| *Friedmanniella sagamiharensis* | CRP | -0.419214 | 0.003019 | negtive |
| *Gordonia iterans* | PRO | -0.419418 | 0.002702 | negtive |
| *Stigmatella aurantiaca* | Cr | -0.419501 | 0.002696 | negtive |
| *Sinomonas sp. R1AF57* | CRP | -0.419702 | 0.002982 | negtive |
| *Mordavella sp. Marseille-P3756* | UA | -0.419992 | 0.002662 | negtive |
| *Faecalibacterium phage FP_oengus* | Cr | -0.420228 | 0.002645 | negtive |
| *Thermoplasmatales archaeon BRNA1* | UA | -0.420265 | 0.002643 | negtive |
| *Acinetobacter sp. TGL-Y2* | CysC | -0.420724 | 0.002905 | negtive |
| *Bifidobacterium kashiwanohense* | CysC | -0.420813 | 0.002899 | negtive |
| *Trichomonas vaginalis* | CRP | -0.421231 | 0.002868 | negtive |
| *Ruminococcus champanellensis* | UA | -0.421319 | 0.002571 | negtive |
| *Micromonospora auratinigra* | UA | -0.42183 | 0.002537 | negtive |
| *Mycolicibacterium rutilum* | ALP | -0.422032 | 0.002524 | negtive |
| *Bifidobacterium scardovii* | CRP | -0.42263 | 0.002767 | negtive |
| *Lactobacillus koreensis* | CRP | -0.422848 | 0.002752 | negtive |
| *Corynebacterium glyciniphilum* | Cr | -0.423525 | 0.002427 | negtive |
| *Prevotella enoeca* | UA | -0.424431 | 0.00237 | negtive |
| *Martelella mediterranea* | Cr | -0.425185 | 0.002324 | negtive |
| *Plantibacter flavus* | BUN | -0.425957 | 0.002277 | negtive |
| *Methanocorpusculum labreanum* | Cr | -0.426245 | 0.002259 | negtive |
| *Aspergillus aculeatus* | PRO | -0.426407 | 0.00225 | negtive |
| *[Eubacterium] sulci* | Cr | -0.426556 | 0.002241 | negtive |
| *Desulfotalea psychrophila* | PRO | -0.427008 | 0.002214 | negtive |
| *Prosthecochloris aestuarii* | PRO | -0.427171 | 0.002205 | negtive |
| *Faecalibacterium prausnitzii* | CysC | -0.427435 | 0.002444 | negtive |
| *Desulfotalea psychrophila* | Cr | -0.427543 | 0.002183 | negtive |
| *Faecalibacterium prausnitzii* | PRO | -0.427896 | 0.002163 | negtive |
| *Streptomyces violaceoruber* | CRP | -0.427966 | 0.002411 | negtive |
| *Clostridium sp. enrichment culture clone 7-14* | UA | -0.428484 | 0.002129 | negtive |
| *Synechococcus sp. JA-2-3B'a(2-13)* | CysC | -0.428905 | 0.002352 | negtive |
| *Acutalibacter muris* | PRO | -0.429081 | 0.002096 | negtive |
| *Acidovorax sp. RAC01* | PRO | -0.429167 | 0.002091 | negtive |
| *Mucilaginibacter gotjawali* | CysC | -0.429308 | 0.002328 | negtive |
| *Rhodoferax koreense* | CRP | -0.429601 | 0.00231 | negtive |
| *Stigmatella aurantiaca* | PRO | -0.429696 | 0.002061 | negtive |
| *Pseudomonas fulva* | CRP | -0.429741 | 0.002301 | negtive |
| *Gemmatirosa kalamazoonesis* | UA | -0.431404 | 0.001969 | negtive |
| *Kluyvera georgiana* | BUN | -0.431968 | 0.00194 | negtive |
| *Actinomyces succiniciruminis* | PRO | -0.431992 | 0.001938 | negtive |
| *Serratia marcescens* | PRO | -0.43237 | 0.001919 | negtive |
| *Micromonospora viridifaciens* | UA | -0.432851 | 0.001894 | negtive |
| *Microbacterium aurum* | BUN | -0.43314 | 0.001879 | negtive |
| *Acidovorax carolinensis* | UA | -0.433311 | 0.001871 | negtive |
| *Rhodoferax koreense* | UA | -0.434119 | 0.00183 | negtive |
| *Ruminococcus bicirculans* | UA | -0.434636 | 0.001805 | negtive |
| *Mahella australiensis* | CRP | -0.434658 | 0.002021 | negtive |
| *Mycolicibacterium rutilum* | UA | -0.434685 | 0.001802 | negtive |
| *Eggerthella sp. YY7918* | UA | -0.43484 | 0.001795 | negtive |
| *Faecalibacterium phage FP_Lugh* | CysC | -0.434942 | 0.002006 | negtive |
| *Prevotella fusca* | CysC | -0.435089 | 0.001998 | negtive |
| *Mahella australiensis* | Cr | -0.435191 | 0.001778 | negtive |
| *Francisella philomiragia* | CRP | -0.435885 | 0.001956 | negtive |
| *Coprococcus sp. ART55/1* | PRO | -0.435927 | 0.001742 | negtive |
| *Cladophialophora bantiana* | WBC | -0.436458 | 0.001717 | negtive |
| *Vibrio campbellii* | BUN | -0.436459 | 0.001717 | negtive |
| *Corynebacterium frankenforstense* | PRO | -0.436834 | 0.0017 | negtive |
| *Brachyspira pilosicoli* | UA | -0.436983 | 0.001693 | negtive |
| *Barnesiella viscericola* | CysC | -0.437152 | 0.001891 | negtive |
| *Erysipelotrichaceae bacterium I46* | ALB | -0.437254 | 0.00168 | negtive |
| *Novosphingobium resinovorum* | CRP | -0.438799 | 0.001809 | negtive |
| *Planococcus sp. MB-3u-03* | CysC | -0.440367 | 0.001734 | negtive |
| *Dictyoglomus turgidum* | CRP | -0.440633 | 0.001722 | negtive |
| *Desulfotalea psychrophila* | BUN | -0.440935 | 0.001519 | negtive |
| *Serratia grimesii* | PRO | -0.441057 | 0.001514 | negtive |
| *Propionibacterium freudenreichii* | CysC | -0.441984 | 0.00166 | negtive |
| *Coprococcus catus* | CRP | -0.443889 | 0.001576 | negtive |
| *Clostridium sp. enrichment culture clone 7-14* | PRO | -0.444242 | 0.001385 | negtive |
| *Clostridiales bacterium CoAT_53-4c* | UA | -0.444701 | 0.001368 | negtive |
| *Faecalibacterium prausnitzii* | Cr | -0.444977 | 0.001357 | negtive |
| *Bifidobacterium choerinum* | BUN | -0.445074 | 0.001354 | negtive |
| *Streptomyces sp. SF2575* | CO2 | -0.445389 | 0.001342 | negtive |
| *Microbacterium sp. XT11* | CRP | -0.446088 | 0.001484 | negtive |
| *Geobacter daltonii* | UA | -0.446455 | 0.001302 | negtive |
| *Devosia sp. H5989* | PRO | -0.447215 | 0.001275 | negtive |
| *Lactobacillus koreensis* | PRO | -0.447838 | 0.001252 | negtive |
| *Azospira oryzae* | UA | -0.448714 | 0.001222 | negtive |
| *Bifidobacterium dentium* | CysC | -0.449 | 0.001369 | negtive |
| *Cryobacterium arcticum* | Cr | -0.449391 | 0.001199 | negtive |
| *Xanthomonas oryzae* | PRO | -0.449399 | 0.001198 | negtive |
| *Microbacterium aurum* | PRO | -0.449486 | 0.001195 | negtive |
| *Faecalibacterium phage FP_Toutatis* | BUN | -0.449998 | 0.001178 | negtive |
| *Spirosoma rigui* | UA | -0.450476 | 0.001162 | negtive |
| *Methylosinus trichosporium* | CRP | -0.450572 | 0.001311 | negtive |
| *Parvimonas micra* | PRO | -0.452042 | 0.001111 | negtive |
| *Prevotella jejuni* | PRO | -0.45215 | 0.001108 | negtive |
| *Faecalibacterium phage FP_Lugh* | Cr | -0.452642 | 0.001093 | negtive |
| *Aeromonas schubertii* | UA | -0.45271 | 0.00109 | negtive |
| *Faecalibacterium phage FP_Toutatis* | CysC | -0.454168 | 0.001185 | negtive |
| *Streptomyces lincolnensis* | PRO | -0.454264 | 0.001043 | negtive |
| *Tessaracoccus sp. T2.5-30* | PRO | -0.454269 | 0.001043 | negtive |
| *Kluyvera georgiana* | Cr | -0.454441 | 0.001038 | negtive |
| *Actinomyces succiniciruminis* | CRP | -0.455742 | 0.001133 | negtive |
| *Bartonella vinsonii* | PRO | -0.455952 | 0.000993 | negtive |
| *Romboutsia ilealis* | CRP | -0.457863 | 0.001067 | negtive |
| *Olsenella sp. oral taxon 807* | UA | -0.458057 | 0.000934 | negtive |
| *Paraburkholderia sprentiae* | UA | -0.459771 | 0.000889 | negtive |
| *Gordonia iterans* | CRP | -0.459837 | 0.001008 | negtive |
| *Jatrophihabitans sp. GAS493* | PRO | -0.46005 | 0.000882 | negtive |
| *Polaribacter vadi* | UA | -0.460408 | 0.000872 | negtive |
| *Paenibacillus alvei* | Cr | -0.460504 | 0.00087 | negtive |
| *Pseudomonas citronellolis* | UA | -0.46071 | 0.000865 | negtive |
| *Prevotella dentalis* | UA | -0.461884 | 0.000835 | negtive |
| *Microbacterium aurum* | Cr | -0.462828 | 0.000812 | negtive |
| *Aspergillus aculeatus* | CRP | -0.46357 | 0.000906 | negtive |
| *[Mycobacterium] stephanolepidis* | HGB | -0.463575 | 0.000795 | negtive |
| *Hymenobacter sp. PAMC 26628* | UA | -0.463829 | 0.000789 | negtive |
| *Prevotella jejuni* | CysC | -0.463914 | 0.000897 | negtive |
| *[Eubacterium] rectale* | CysC | -0.465054 | 0.000867 | negtive |
| *Agromyces sp. 30A* | CRP | -0.465107 | 0.000866 | negtive |
| *Micromonospora viridifaciens* | CRP | -0.465471 | 0.000857 | negtive |
| *Prevotella ruminicola* | CysC | -0.465869 | 0.000847 | negtive |
| *Geobacter metallireducens* | PRO | -0.466354 | 0.000732 | negtive |
| *Aspergillus aculeatus* | Cr | -0.467755 | 0.000702 | negtive |
| *Caldicellulosiruptor hydrothermalis* | UA | -0.46816 | 0.000693 | negtive |
| *Vibrio campbellii* | CysC | -0.46824 | 0.00079 | negtive |
| *Pseudomonas fulva* | PRO | -0.468462 | 0.000687 | negtive |
| *Planococcus sp. MB-3u-03* | Cr | -0.469468 | 0.000666 | negtive |
| *Mycolicibacterium thermoresistibile* | Cr | -0.470506 | 0.000646 | negtive |
| *Pasteurellaceae bacterium NI1060* | PRO | -0.470865 | 0.000639 | negtive |
| *Faecalibacterium phage FP_Toutatis* | Cr | -0.471092 | 0.000635 | negtive |
| *Bifidobacterium breve* | PRO | -0.472524 | 0.000608 | negtive |
| *Sulfurifustis variabilis* | UA | -0.472672 | 0.000605 | negtive |
| *Streptomyces violaceoruber* | UA | -0.474526 | 0.000572 | negtive |
| *Libanicoccus massiliensis* | UA | -0.476477 | 0.000538 | negtive |
| *Oceanithermus profundus* | UA | -0.477101 | 0.000528 | negtive |
| *Bifidobacterium choerinum* | Cr | -0.477736 | 0.000518 | negtive |
| *Thermoplasmatales archaeon BRNA1* | CRP | -0.477797 | 0.000594 | negtive |
| *Microbacterium aurum* | CysC | -0.47972 | 0.00056 | negtive |
| *Chromohalobacter salexigens* | UA | -0.479841 | 0.000485 | negtive |
| *Thermaerobacter marianensis* | UA | -0.481018 | 0.000468 | negtive |
| *Martelella mediterranea* | PRO | -0.481318 | 0.000463 | negtive |
| *Corynebacterium glyciniphilum* | CRP | -0.481644 | 0.000528 | negtive |
| *Bifidobacterium choerinum* | CysC | -0.482031 | 0.000522 | negtive |
| *Rhizobium sp. ACO-34A* | PRO | -0.483222 | 0.000436 | negtive |
| *Bordetella bronchiseptica* | PRO | -0.484208 | 0.000423 | negtive |
| *Thermincola potens* | UA | -0.485407 | 0.000407 | negtive |
| *Slackia heliotrinireducens* | UA | -0.488825 | 0.000365 | negtive |
| *Plantibacter flavus* | PRO | -0.488889 | 0.000365 | negtive |
| *Gordonibacter pamelaeae* | UA | -0.489081 | 0.000362 | negtive |
| *Bifidobacterium choerinum* | CRP | -0.489234 | 0.000418 | negtive |
| *Plantibacter flavus* | Cr | -0.489419 | 0.000359 | negtive |
| *Bifidobacterium choerinum* | PRO | -0.491438 | 0.000336 | negtive |
| *Pelodictyon luteolum* | UA | -0.492342 | 0.000326 | negtive |
| *Mycolicibacterium thermoresistibile* | PRO | -0.493093 | 0.000319 | negtive |
| *Kiritimatiella glycovorans* | UA | -0.493465 | 0.000315 | negtive |
| *Bifidobacterium angulatum* | PRO | -0.493751 | 0.000312 | negtive |
| *Caldicellulosiruptor saccharolyticus* | PRO | -0.495269 | 0.000297 | negtive |
| *Bifidobacterium adolescentis* | PRO | -0.496508 | 0.000285 | negtive |
| *[Eubacterium] rectale* | PRO | -0.498125 | 0.00027 | negtive |
| *Methylobacterium nodulans* | CRP | -0.498699 | 0.000309 | negtive |
| *Desulfovibrio salexigens* | UA | -0.499604 | 0.000257 | negtive |
| *Caldicellulosiruptor saccharolyticus* | CRP | -0.49962 | 0.0003 | negtive |
| *Salmonella bongori* | PRO | -0.499728 | 0.000256 | negtive |
| *Gordonibacter urolithinfaciens* | UA | -0.500255 | 0.000252 | negtive |
| *Denitrobacterium detoxificans* | UA | -0.500408 | 0.000251 | negtive |
| *Isoptericola dokdonensis* | CRP | -0.500816 | 0.000289 | negtive |
| *Actinomyces succiniciruminis* | UA | -0.500816 | 0.000247 | negtive |
| *Devosia sp. H5989* | CRP | -0.502292 | 0.000275 | negtive |
| *[Eubacterium] rectale* | Cr | -0.505141 | 0.000214 | negtive |
| *Pectobacterium polaris* | UA | -0.506346 | 0.000206 | negtive |
| *Streptomyces lincolnensis* | BUN | -0.50712 | 0.0002 | negtive |
| *Acidovorax avenae* | UA | -0.508623 | 0.00019 | negtive |
| *Erysipelotrichaceae bacterium I46* | HGB | -0.508633 | 0.00019 | negtive |
| *Bifidobacterium kashiwanohense* | PRO | -0.511492 | 0.000173 | negtive |
| *Plantibacter flavus* | CRP | -0.511697 | 0.000201 | negtive |
| *Candidatus Arthromitus sp. SFB-rat-Yit* | PRO | -0.515251 | 0.000152 | negtive |
| *Thermus thermophilus* | UA | -0.516443 | 0.000145 | negtive |
| *Planococcus sp. PAMC 21323* | PRO | -0.518065 | 0.000137 | negtive |
| *Thermoanaerobacterium saccharolyticum* | PRO | -0.51939 | 0.000131 | negtive |
| *Bacillus sp. OxB-1* | PRO | -0.520398 | 0.000127 | negtive |
| *Streptomyces lincolnensis* | Cr | -0.522824 | 0.000116 | negtive |
| *Stigmatella aurantiaca* | UA | -0.523659 | 0.000113 | negtive |
| *Planococcus sp. MB-3u-03* | BUN | -0.525352 | 0.000106 | negtive |
| *Acutalibacter muris* | UA | -0.526512 | 0.000102 | negtive |
| *Bacillus oceanisediminis* | PRO | -0.527459 | 9.85E-05 | negtive |
| *Dialister sp. Marseille-P5638* | PRO | -0.528254 | 9.57E-05 | negtive |
| *Brachyspira pilosicoli* | PRO | -0.537633 | 6.79E-05 | negtive |
| *Propionibacterium freudenreichii* | CRP | -0.53855 | 7.86E-05 | negtive |
| *[Eubacterium] rectale* | BUN | -0.540199 | 6.17E-05 | negtive |
| *Methanocorpusculum labreanum* | PRO | -0.542164 | 5.73E-05 | negtive |
| *Clavibacter michiganensis* | CRP | -0.543107 | 6.65E-05 | negtive |
| *Ottowia sp. oral taxon 894* | UA | -0.54767 | 4.64E-05 | negtive |
| *Planococcus sp. MB-3u-03* | PRO | -0.553427 | 3.72E-05 | negtive |
| *Olsenella umbonata* | UA | -0.56317 | 2.52E-05 | negtive |
| *Thermoplasmatales archaeon BRNA1* | PRO | -0.568102 | 2.06E-05 | negtive |
| *Tessaracoccus sp. T2.5-30* | UA | -0.569583 | 1.94E-05 | negtive |
| *Coprococcus catus* | PRO | -0.573151 | 1.68E-05 | negtive |
| *Microbacterium sp. XT11* | PRO | -0.575041 | 1.55E-05 | negtive |
| *[Ruminococcus] torques* | PRO | -0.590291 | 8.04E-06 | negtive |
| *Phoenicibacter massiliensis* | PRO | -0.62042 | 1.98E-06 | negtive |

**Table S8. Spearman correlation between metabolites and clinical indicators**

| **Metabolites** | **Clinical indicators** | **R value** | ***P* value** | **Relation** |
| --- | --- | --- | --- | --- |
| (S)-2-aminobutyric acid | BUN | 0.3866216 | 0.009531 | positive |
| (S)-2-aminobutyric acid | Cr | 0.3969692 | 0.00763 | positive |
| (S)-2-aminobutyric acid | PRO | 0.4109339 | 0.00559 | positive |
| 1,3,5(10)-Estratrien-3,17.beta.-diol 17-glucosiduronate | CRP | 0.4437729 | 0.002875 | positive |
| 11beta-Hydroxyprogesterone | BUN | -0.326567 | 0.0305 | negtive |
| 11beta-Hydroxyprogesterone | Cr | -0.326414 | 0.030582 | negtive |
| 11beta-Hydroxyprogesterone | CysC | -0.321112 | 0.035766 | negtive |
| 2-Isopropylmalic acid | CysC | -0.425556 | 0.004442 | negtive |
| 2-Isopropylmalic acid | BUN | -0.404948 | 0.006397 | negtive |
| 2-Isopropylmalic acid | Cr | -0.393374 | 0.00825 | negtive |
| 2-Isopropylmalic acid | ALP | -0.373498 | 0.012515 | negtive |
| 2-Isopropylmalic acid | PRO | -0.321475 | 0.033347 | negtive |
| 3-Aminosalicylic acid | BUN | 0.364982 | 0.01485 | positive |
| 3-Aminosalicylic acid | Cr | 0.3792071 | 0.011131 | positive |
| 3b-Hydroxy-5-cholenoic acid | CRP | -0.386939 | 0.010369 | negtive |
| 3b-Hydroxy-5-cholenoic acid | PRO | -0.373388 | 0.012543 | negtive |
| 3b-Hydroxy-5-cholenoic acid | CO2 | 0.4830007 | 0.000897 | positive |
| 3-Hydorxy-3-methylglutaric acid | CysC | -0.340143 | 0.025631 | negtive |
| 3-Hydorxy-3-methylglutaric acid | CRP | -0.334192 | 0.028505 | negtive |
| 6k-PGF1alpha-d4 | CRP | -0.53504 | 0.000219 | negtive |
| 6k-PGF1alpha-d4 | Cr | -0.380969 | 0.010731 | negtive |
| 6k-PGF1alpha-d4 | BUN | -0.360964 | 0.016075 | negtive |
| 6k-PGF1alpha-d4 | HGB | 0.3313476 | 0.028012 | positive |
| 9,10-DiHOME | Cr | -0.330361 | 0.028511 | negtive |
| 9,10-DiHOME | HGB | 0.3377631 | 0.024941 | positive |
| alpha-ketoglutarate | HGB | -0.35151 | 0.019297 | negtive |
| alpha-ketoglutarate | UA | 0.3178743 | 0.03549 | positive |
| alpha-ketoglutarate | PRO | 0.4678085 | 0.001365 | positive |
| alpha-ketoglutarate | CysC | 0.4766832 | 0.001231 | positive |
| alpha-ketoglutarate | BUN | 0.4820611 | 0.000922 | positive |
| alpha-ketoglutarate | Cr | 0.5356123 | 0.000179 | positive |
| Aminopterin | ALB | -0.517587 | 0.000321 | negtive |
| Aminopterin | HGB | -0.403962 | 0.00654 | negtive |
| Aminopterin | Cr | 0.4644934 | 0.001492 | positive |
| Aminopterin | CysC | 0.5049277 | 0.000554 | positive |
| Aminopterin | BUN | 0.5101149 | 0.000404 | positive |
| Arg-Glu | CRP | -0.348268 | 0.022101 | negtive |
| Arg-Glu | HGB | 0.346223 | 0.021325 | positive |
| Betaine | Cr | -0.364828 | 0.014896 | negtive |
| Betaine | PRO | -0.359465 | 0.016553 | negtive |
| Betaine | BUN | -0.328258 | 0.029599 | negtive |
| Betaine | ALB | 0.3771058 | 0.011624 | positive |
| Cytidine | CysC | -0.491863 | 0.000808 | negtive |
| Cytidine | Cr | -0.369692 | 0.013517 | negtive |
| Cytidine | UA | -0.346138 | 0.021359 | negtive |
| Cytidine | ALB | 0.3322055 | 0.027584 | positive |
| Cytidine | HGB | 0.3407241 | 0.023621 | positive |
| D-Glucono-1,5-lactone | Cr | -0.427841 | 0.003768 | negtive |
| D-Glucono-1,5-lactone | BUN | -0.419046 | 0.004637 | negtive |
| D-Glucono-1,5-lactone | CysC | -0.389306 | 0.009871 | negtive |
| D-Glucono-1,5-lactone | PRO | -0.344358 | 0.022082 | negtive |
| D-Glucono-1,5-lactone | ALB | 0.318672 | 0.035006 | positive |
| D-Glucono-1,5-lactone | PLT | 0.3211643 | 0.033528 | positive |
| D-Glucono-1,5-lactone | HGB | 0.3271882 | 0.030166 | positive |
| Dioscin | ALP | -0.318379 | 0.035183 | negtive |
| L-Glutamate | CysC | 0.313182 | 0.040863 | positive |
| L-Glutamate | BUN | 0.406217 | 0.006218 | positive |
| L-Glutamate | Cr | 0.4128282 | 0.005353 | positive |
| L-Glutamate | PRO | 0.4238195 | 0.004146 | positive |
| L-Pipecolic acid | CRP | -0.426897 | 0.004305 | negtive |
| L-Pipecolic acid | UA | -0.386101 | 0.009636 | negtive |
| L-Pipecolic acid | BUN | -0.355466 | 0.017888 | negtive |
| L-Pipecolic acid | CysC | -0.341653 | 0.024942 | negtive |
| L-Pipecolic acid | Cr | -0.341004 | 0.023499 | negtive |
| L-Pipecolic acid | HGB | 0.3534845 | 0.018582 | positive |
| L-Pipecolic acid | CO2 | 0.5198612 | 0.000298 | positive |
| N1-Methyl-2-pyridone-5-carboxamide | HGB | -0.507808 | 0.000434 | negtive |
| N1-Methyl-2-pyridone-5-carboxamide | CO2 | -0.330121 | 0.028633 | negtive |
| N1-Methyl-2-pyridone-5-carboxamide | PRO | 0.3026648 | 0.045829 | positive |
| N1-Methyl-2-pyridone-5-carboxamide | BUN | 0.5146261 | 0.000352 | positive |
| N1-Methyl-2-pyridone-5-carboxamide | CysC | 0.5179927 | 0.000374 | positive |
| N1-Methyl-2-pyridone-5-carboxamide | Cr | 0.5253921 | 0.00025 | positive |
| N6,N6,N6-Trimethyl-L-lysine | CRP | -0.334116 | 0.028543 | negtive |
| N6,N6,N6-Trimethyl-L-lysine | Cr | -0.328317 | 0.029568 | negtive |
| N6,N6,N6-Trimethyl-L-lysine | PRO | -0.310441 | 0.040274 | negtive |
| N6,N6,N6-Trimethyl-L-lysine | HGB | 0.3082238 | 0.041798 | positive |
| N-Acetyl-D-lactosamine | Cr | -0.473022 | 0.001184 | negtive |
| N-Acetyl-D-lactosamine | PRO | -0.429522 | 0.003619 | negtive |
| N-Acetyl-D-lactosamine | CysC | -0.390515 | 0.009625 | negtive |
| N-Acetyl-D-lactosamine | BUN | -0.381617 | 0.010587 | negtive |
| N-Acetyl-D-lactosamine | ALB | 0.335448 | 0.026015 | positive |
| N-Acetyl-D-lactosamine | HGB | 0.4813705 | 0.00094 | positive |
| N-Acetyl-L-Histidine | CRP | -0.404723 | 0.007102 | negtive |
| N-Acetyl-L-Histidine | Cr | -0.38837 | 0.009184 | negtive |
| Perseitol | TBA | 0.3077792 | 0.04211 | positive |
| Phenol | HGB | -0.336142 | 0.025689 | negtive |
| Phenol | CysC | 0.3282861 | 0.031615 | positive |
| Phenol | Cr | 0.3804758 | 0.010842 | positive |
| Promethazine | CRP | -0.378917 | 0.01222 | negtive |
| Promethazine | CO2 | 0.3697356 | 0.013505 | positive |
| Ramipril | ALB | -0.405442 | 0.006327 | negtive |
| Ramipril | Cr | 0.3052687 | 0.043903 | positive |
| Ramipril | BUN | 0.3100021 | 0.040572 | positive |
| Ramipril | CysC | 0.3229241 | 0.034677 | positive |
| Uridine | PRO | -0.348283 | 0.020515 | negtive |

**Table S9. Area under the ROC curves of microbial biomarkers**

| **Species** | **AUC** |
| --- | --- |
| *Tannerella forsythia* | 0.8295 |
| *Faecalibacterium prausnitzii* | 0.8253 |
| *Faecalibacterium sp.* | 0.8168 |
| *Barnesiella viscericola* | 0.8126 |
| *Bifidobacterium kashiwanohense* | 0.8063 |
| *Bifidobacterium adolescentis* | 0.7937 |
| *【Eubacterium】 rectale* | 0.7916 |
| *Coprococcus catus* | *0.7579* |
| *Phoenicibacter massiliensis* | *0.7579* |
| *Dialister sp.Marseille-P5638* | 0.7495 |
| *Bacteroides heparinolyticus* | 0.7495 |
| *Ruminococcus bicirculans* | 0.7411 |
| *Coprococcus sp.ART55/1* | 0.7368 |
| *Desulfovibrio piger* | 0.7284 |
| *【Eubacterium】 sulci* | 0.7263 |
| *【Eubacterium】 siraeum* | 0.7158 |
| *Bifidobacterium pseudocatenulatum* | 0.7116 |
| *Ruminococcus champanellensis* | 0.7116 |
| *【Ruminococcus】 torques* | 0.7053 |
| *Bifidobacterium breve* | 0.7011 |
| *Roseburia intestinalis* | 0.6863 |
| *Lactobacillus johnsonii* | 0.6842 |
| *Blautia obeum* | 0.6779 |
| *Acidaminococcus fermentans* | 0.6779 |
| *Lactobacillus fermentum* | 0.6716 |
| *Mordavella sp.Marseille-P3756* | 0.6611 |
| *butyrate-producing bacterium SS3/4* | 0.6589 |
| *Roseburia hominis* | 0.6547 |
| *Turicibacter sp.H121* | 0.64 |
| *Streptococcus sp.I-P16* | 0.6316 |
| *Romboutsia ilealis* | 0.6232 |
| *Clostridium chauvoei* | 0.6105 |
| *Anaerostipes hadrus* | 0.6084 |
| *Elizabethkingia miricola* | 0.6063 |
| *Lachnoclostridium phocaeense* | 0.6042 |
| *【Clostridium】 sphenoides* | 0.5832 |
| *Burkholderiales bacterium YL45* | 0.5768 |
| *Candidatus Saccharibacteria oral taxon TM7x* | 0.5768 |
| *Lachnoclostridium sp.YL32* | 0.5705 |
| *Clostridioides difficile* | 0.5621 |
| *Cupriavidus metallidurans* | 0.5579 |
| *Klebsiella phage KP8* | 0.5411 |
| *Fusobacterium varium* | 0.5389 |
| *【Clostridium】 saccharolyticum* | 0.5284 |
| *Actinomyces pacaensis* | 0.5284 |
| *Klebsiella variicola* | 0.4926 |
| *Streptococcus sanguinis* | 0.4653 |
| *Enterococcus phage IME-EFm1* | 0.4526 |
| *butyrate-producing bacterium SM4/1* | 0.4526 |
| *Actinomyces meyeri* | 0.4316 |

**Table S10. Antibiotic resistance and virulence genes of key species associated with AMR after kidney transplantation**

| **Species** | **Virulence gene** | **Antibiotic resistance gene** |
| --- | --- | --- |
| *Tannerella forsythia* | coxFIC1, neuB, per, cap8M, aslA, fepC, bprB, algC, oatA, fimE, nagL, wbcC, wbkA, mgtB, kpsF, wbcJ, ddhB, kfoC, lsgC, fepA, wcbT, prsA2, hlyD, hlyB, futC, Cj1135, chuV, gluP, gmhA/lpcA, pvdO, algB, licD, galE, clpC, cylG, pgaC, rffG, flhF, pdgA, algU, cpsJ, tviB, bfmR, yhxB/manB, pchD, Cj1437c, manC, clbI, tagT, acrB, eptC, clbG, adeG | SAT-4, macB, Bifidobacterium ileS conferring resistance to mupirocin, srmB, tetA(48), vanXA, vanTrL, mef(B), mel, AcrE, mtrC, arnA, rosB, AAC(6')-Isa, lsaA, cpxA, tsnR, mtrD, MCR-1.6, adeJ, macA, bcrA |
| *Faecalibacterium prausnitzii* | pilH, algU, clbM, AHA_1389, iap/cwhA, cheY, recN, iroC, phoR, galF, rffG, bopD, cylG, cylI, fabZ, phoP, pilR, hspX, tlpB, cdpA, flmH, cheW-2, coxH2, bsc1, bfmR, pspA, fbpA, exsA, hlyB, ybtP, fbpC, pchR, lpg2936, hitC, algB, lap, pdxA, clpP, feoB, feoA, cylA, bfmS, kdsA, wbkC, chpD, manC, algI, kdtB, yhxB/manB, wzm, Cj1432c, pvdL, lytC, tagB-5, CBU_1434, fepC, fliA, htpB, cgs, pvdN, Cj1437c, cpsJ, cpsD, cps4B, sfaX, msbA, pvdM, lirB, fimE, bauE, inlF, pce, essC, fimB, gtrB, gtrA, prt, per, ddhB, ddhA, wzt, cap8D, lpxE, pppA, algR, pscN, cdsN, rtxA, cps4A, virD4, bplC, pdgA, mgtB, cps4E, yscN, eccA1, pvdH, fctB, fctA, sipA, cpsE, fbpB, cpsL, cpsG, cap8O, clpC, plcD, pebA, cpsA, cpsB, mucP, wzb, ppkA, hlyD, chuW, relA, wbpL, bplA, allS, entE, Cj1436c, shuU, gspG, bexA, gtcA, msrA/B(pilB), clbJ, hasC, llsG, hddC, flgR, hldD, adsA, ptxR, lspA, IlpA, hlyA, cps4H, Cj1438c, vipF, lsgC, bepA, pkn5, lspG, hgpC, clbB, mtrD, ricA, ebpC, srtC4, caf1R, coxFIC1, algZ, phzH, srtC-1/srtB, pilB, fsrA, chuV, clbG, ybtA, orfM, mgtC, acpXL, fliB, lspE, wcbQ, etgA, kfoC, cba, wbcA, rtxB, ybtQ, fleN, cpsK, kdsB, allB, cpsO, mf3, pilD, wbkA, cps4F, cps4K, EF3023, mucD, flhF, exeG, pfbA, cyaB, bprB, ugd, bprC, virB4, cps4C, papX, fleS/flrB, algJ, irtB, allR, fsrC, virB11, srtB, aslA, iroD, vpdC, wcsT, neuD, lpeA, mntA, fliI, ascN, pgaB, galE, glf, acrB, tssH-5/clpV, srtC, p97, algW, fleR/flrC, eccA3, algC, neuB, spaD, lipF, spvR, cheA, irp1, CBU_1566, ylxH, chpA, kpsT, capA, clbP, icaR, clbA, sda, wbbO, galU, srtG1, tagT, clpE, wcbJ, kpsF, cps4I, cpsI, wbbM, lgtC, icaA, irtA, cap8J, bplH, shuV, pvdI, pgaC, bsh, wcaJ, tviC, cpsC, wbaP/rfbP, inlB, lasA, pilS, rpoN, ureG, cylR2, ibeA, srtG2, entA, iraA, lpg2370, wecA, isdE, ideR, fes, srtD, inlJ, inlA, mntB, AHA_3493, nanI, wbpB, cap8B, tviE, wcbT, cap8C, clbD, cpsM, hldE, chuX, Cj1138, barB, lpsB/lpcC, cpsN, adeF, toxA, rrgB, chuU, cheB, lidL, fleQ/flrC, gnd, Cj1427c, allC, lmb, clbS, ipgF, manB, wcbE, wcsS, clbF, eccCb5, luxS, shuT, tapD, pilE, mshG, gbs0631, bauD, bauC, pchD, waaV, clbK, allD, rtxE, barA, pseA, cloSI, pilT, pilC, llsB, lpxD, pilX, bsaS, dep/capD, vscN, mbtJ, futA, tagAB-5, bscN, ankN/ankX/legA8, neuC, ccmA, rfaE, wcaI, icaB, clbI, aur, kfiC, licD, licC, wbpZ, lafS, cpa, pvdE, pvdJ, wbcG, nanB, icmE, wzx, cheB-2, lsgF, cps4G, Cj1417c, fleR, fleQ, CBU_0062, bsaN, gbs0628, ceg34, psaA, srtC1, pitB, btpA, ctrD, fbaB, pavA, cps4J, wcbR, fliC, fliD, fliW, pchI, clbC, pspC/cbpA, ebpA, cps4L, cap8F, iga, vip, cap8M, hasB, bauB, fepD, iroB, afaG-VII, tcnA, chuT, bepC, fimK, farB, lgtA, lsgA, fepB, pilW, flpF, rfpB, Cj1136, waaG, wbbN, gmd, fcl, tcpN/toxT, rhlC, bplF, Cj1434c, srtE, fss1, clbL, lpsA, spa47, nanH, capC, capB, pilG, bplE, tviB, wcbL, wcbN, gmhA, algA, vscN2, ptmB, xcpA/pilD, scpA, cps4D, flaB, clpV/tssH, phzF1, wbcH, nagK, ssaN, phzE1, ybtS, wcbP, cap8N, mucC, dotB, entF, pvdD, flhG, AHA_1831, neuA, bplB, flgJ, algD, farA, cheV3, cap8E, tcpI, Cj1420c, cylZ, Cj1137c, pvdS, phzD1, escN, manAoAg, aut, plcA, fsrB, cheD, lfgL, iroE, vasH/clpV, p30/p32, pilA, acm, gtrII, wzt2, eccA5, cap8P, xcpS, mshE, gmhA2, bplG, pmm, cbpA, lgtF, oatA, vipD, mtrC, rfbD, prsA2, pipB2, fbp54, katA, p200, tcpC, cna, entB, licA, licB, geh, lip, clbN, cdsD, colA, lpnE, cbpD, cheV, flgG, flhA, flhB, fliP, fliN, fliM, pomA2, flgE, flgD, fliG, fliF, fliE, flgC, phtA, mbtL, flmD, nueB, wcbM, futB, zmpC, gspE, cdtA, ylfB/legC2, wcbK, neuA1, srtC-2/srtC, xcpT, nanJ | kdpE, cdeA, tetM, SAT-3, evgS, blaI, golS, patA, MexR, vanSO, vanHD, evgA, vanRI, AAC(3)-Ic, Pseudomonas aeruginosa CpxR, vanSD, MdtK, efrB, Escherichia coli soxS with mutation conferring antibiotic resistance, lsaC, poxtA, vgaE, vgaB, gadX, carA, vmlR, LRA-3, tetA(60), Bifidobacterium ileS conferring resistance to mupirocin, tlrC, arnA, adeR, marA, oleB, srmB, QnrA6, mexM, gadW, tetT, Shigella flexneri parC conferring resistance to fluoroquinolones, QnrB20, rosB, Chlamydia trachomatis intrinsic murA conferring resistance to fosfomycin, PmrF, vanHM, emrR, novA, lsaB, smeR, arlR, vanTE, CTX-M-159, mecI, vanC, baeR, vgaA, abcA, tet(D), THIN-B, eatAv, mel, Escherichia coli UhpA with mutation conferring resistance to fosfomycin, bcrC, Enterococcus faecalis chloramphenicol acetyltransferase, vanUG, mepA, mtrA, Streptococcus pneumoniae PBP2b conferring resistance to amoxicillin, patB, tsnR, tetS, hmrM, ramA, vanRO, Bacillus clausii chloramphenicol acetyltransferase, clbB, cpxA, YojI, Enterococcus faecium cls conferring resistance to daptomycin, vgaD, Escherichia coli EF-Tu mutants conferring resistance to kirromycin, clbC, LRA-12, PmpM, msrC, Erm(37), lmrD, macB, vanRG, vanXYG, vanSB, mecB, vanE, Pseudomonas aeruginosa soxR, tet(35), Enterococcus faecalis cls with mutation conferring resistance to daptomycin, RlmA(II), NmcR, mecD, vanRA, Bifidobacterium adolescentis rpoB mutants conferring resistance to rifampicin, pgpB, abeM, mtrR, mecC, vanXYE, Escherichia coli fabI mutations conferring resistance to isoniazid and triclosan, lsaA, MexZ, mexN, tetA(46), tetX, optrA, KHM-1, myrA, vanSE, mepR, vgaALC, TEM-208, Streptococcus pneumoniae PBP2x conferring resistance to amoxicillin, vanHO, Staphylococcus aureus fusA with mutation conferring resistance to fusidic acid, lsaE, VIM-23, otrC, LRA-2, adeL, mecA, tet37, tet44, vanSN, ugd, vanRF, BJP-1, vanRD, vanSC, vanSF, catB9, msrE, ROB-1, vanTN, LlmA 23S ribosomal RNA methyltransferase, apmA, Streptomyces rishiriensis parY mutant conferring resistance to aminocoumarin, TaeA, msrA, ceoB, Escherichia coli nfsA mutations conferring resistance to nitrofurantoin, smeE, efrA, baeS, salA, vanRN, macA, bacA, MexF, smeS, AAC(3)-Ia, basS, LRA-13, MexL, TriA, rphA, norA, PEDO-1, D-Ala-D-Ala ligase, vanYB, vanTrL, Mycobacterium tuberculosis gidB mutation conferring resistance to streptomycin, mecR1, Erm(31), vanWI, Haemophilus influenzae PBP3 conferring resistance to beta-lactam antibiotics, VatI, vanRB, vanHF, tetQ, Escherichia coli EF-Tu mutants conferring resistance to Pulvomycin, AAC(2')-Id, oleC, vanSL, FEZ-1, tetW, AAC(6')-Iz, Neisseria meningititis PBP2 conferring resistance to beta-lactam, Pseudomonas aeruginosa catB7, vanSA, AAC(6')-Ir, CRP, efmA, AAC(6')-Iak, MexS, vanRM, ACC-4, cfrC, vanRE, nalC, AAC(3)-IIIa, vatD, LRA-8, vanRC, tet(W/N/W), Rm3, adeN, tet(V), tetB(46), vanVB, AAC(6')-Ie-APH(2'')-Ia, mgrA, bcrA, MexB, lmrC, mdtF, vanXYC, SAT-4, otr(A), tet32, VIM-30, adeS, ErmN, QnrB11, LRA-19, adeF, vanZA, Escherichia coli soxR with mutation conferring antibiotic resistance, tetA(48), mdtN, Erm(42), Planobispora rosea EF-Tu mutants conferring resistance to inhibitor GE2270A, Staphylococcus aureus pgsA mutations conferring resistance to daptomycin, tet36, Mycoplasma hominis parC conferring resistance to fluoroquinolone, acrB, cmeR, vanHA, bcrB, Erm(34), eptA, MCR-1.10, Staphylococcus mupA conferring resistance to mupirocin, QnrB44, AAC(6')-Isa, OXA-665, VIM-38, Escherichia coli gyrA conferring resistance to fluoroquinolones, vatE, vanTG, Escherichia coli marR mutant conferring antibiotic resistance, tetA(P), Mycobacterium tuberculosis kasA mutant conferring resistance to isoniazid, AcrS, farA, vanHB, OXA-61, vanRL, MSI-1, chrB, blaR1, LRA-9, SAT-2, mdsB, Pseudomonas aeruginosa parE conferring resistance to fluoroquinolones, tetB(60), vatA, mdsA, vanSG, mtrC, msbA, ErmB, BahA, MIR-11, vanKI, mdtA, arlS, QnrB64, sdiA, AxyY, ErmT, Pseudomonas mutant PhoQ conferring resistance to colistin, QnrB9, AAC(6')-Ih, amrB, AAC(6')-Iy, adeG, vanSM, L1 beta-lactamase, QnrS8, tlrB conferring tylosin resistance, Staphylococcus mupB conferring resistance to mupirocin, SPG-1, catB2, nalD, mtrD, LpeA, rgt1438, tet(K), AAC(6')-Ij, vanF, vanN, vanWB, Escherichia coli acrR with mutation conferring multidrug antibiotic resistance, vanM, QnrD2, AIM-1, vatC, LRA-17, vanTC, dfrA8, vanYG1, Pseudomonas mutant PhoP conferring resistance to colistin, tetR, mefE, Mycobacterium tuberculosis folC with mutation conferring resistance to para-aminosalicylic acid, vanZF, ceoA, Escherichia coli EF-Tu mutants conferring resistance to Enacyloxin IIa, oleD, vatH, QnrC, mef(B), Agrobacterium fabrum chloramphenicol acetyltransferase, Mrx, MexC, cmeA, Staphylococcus aureus cls conferring resistance to daptomycin, APH(3')-Ia, vanA, TriC, Salmonella serovars soxS with mutation conferring antibiotic resistance, FOX-8, tetB(P), mdtE, SMB-1, tetO, vatB, ErmF, Mycobacterium tuberculosis pncA mutations conferring resistance to pyrazinamide, Staphylococcus intermedius chloramphenicol acetyltransferase, GOB-1, catB3, AAC(3)-VIIIa, Lactobacillus reuteri cat-TC, acrD, vanXF, AAC(6')-Iad, CMY-19, QnrVC3, vanWG, Sed-1, catIII, AAC(3)-IIc, ANT(3'')-IIa, mefA, QnrB48, catB8, vanG, QnrB66, mexQ, MexV, MCR-1, AAC(6')-33, AAC(6')-Iaj, Clostridium butyricum catB, cmrA, vanXYL, QnrVC7, Pseudomonas aeruginosa catB6, ErmG, Bacillus subtilis pgsA with mutation conferring resistance to daptomycin, QnrB4, tap, Mycobacterium leprae gyrB conferring resistance to fluoroquinolone, cfrA, vanL, rpoB2, Escherichia coli PtsI with mutation conferring resistance to fosfomycin, pmrA, QnrB13 |
| *Faecalibacterium sp.* | unknown | unknown |
| *Barnesiella viscericola* | hpt, acpXL, cpsI, adsA, kpsF, wbcA, wcbT, iroN, scpB, fcl, cpsO, waaA, iraB, pvdM, adeF, neuA, pdxJ, CBU_1434, bprB, wcaH, orfM, flmH, phoP, chuA, chuU, fepC, lpxB, flgJ, rtxB, clbG, aslA, bfmS, cpsJ, lsgC, motD, hlyB, phoR, fimE, relA, Cj1437c, msbA, pgaC, ompA, fbpC, hitC, lidL, hspX, wza, wzc, wbkA, wbpZ, rffG, hasC, algD, oatA, algU, pvdS, nagL, nagI, mgtC, vpdC, Cj1435c, yhxB/manB, adeG, exsA, wcaJ, manAoAg, CBU_1566, pilG, fleQ, mntB, algB, cylA, farA, pdxA, hlyA, speB, gnd, bauE, mucD, mip, kfoC, algI, fepA, flhG, wbkC, CBU_1594, wecA, waaF, cylI, bplB, pvdO, coxFIC1, tviC, nanH, bplA, fimB, clbM, lpg1661, manC, lgtC, cap8N, pdgA, pchR, cap8J, pchD, phzH, fleS/flrB, mgtB, rfaE, colA, fyuA, bsc1, lpg2936, iap/cwhA, pvdH, lbtC, chpD, eptC, allB, entE, htrB, icaA, irtA, plcD, mtrD, mtrC, galE, shuU, yscN, pscN, ureG, gmhB, algR, algZ, mtrE, shuA, cpsA, mntA, psaA, ybtQ, ybtP, cylF, lplA1, bfmR, gtrB, gtcA, ssaN, algC, inlJ, lsgF, adeH, clbF, ibeA, cps4I, wbcG, ricA, gmd, mucP, cadF, wcbL, acrB, CBU_2076, bsh, htpB, feoB, cyaB, clpC, nanI, gluP, legS2, pilR, nagJ, lpxK, chuV, recN, sodB, lpxD, lpxC, lpxA, eccA1, rfpB, cap8E, cap8F, cap8G, chuW, lpg2370, lap, farB, cps4G, cps4F, csgD, bplH, tviE, lipF, vipD, AHA_1389, kdtB, cpsM, Cj1432c, wbbO, iraA, lgtA, ctrD, kdsB, manB, hxuC, caf1R, AHA_3493, bopD, lirB | PEDO-1, arnA, PmrF, Pseudomonas aeruginosa emrE, Staphylococcus aureus fusE with mutation conferring resistance to fusidic acid, tetM, mexM, tetT, mtrR, sdiA, Staphylococcus mupA conferring resistance to mupirocin, adeR, lmrC, optrA, vanRB, bacA, vgaE, vanSD, efrB, tlrC, salA, YojI, lsaE, Mycoplasma hominis parC conferring resistance to fluoroquinolone, ugd, Bifidobacterium ileS conferring resistance to mupirocin, patB, CfxA4, vanHB, tet37, hmrM, rosB, tet(W/N/W), opcM, mexN, Klebsiella pneumoniae acrA, emrY, Erm(41), vanRO, lsaB, tetB(46), TriB, clbC, mecD, vanC, vanTN, Bacillus clausii chloramphenicol acetyltransferase, sul1, Escherichia coli EF-Tu mutants conferring resistance to kirromycin, Haemophilus influenzae PBP3 conferring resistance to beta-lactam antibiotics, vanHF, vatE, mepA, tsnR, Staphylococcus mupB conferring resistance to mupirocin, tet(Z), oleB, tet(D), apmA, Streptomyces rishiriensis parY mutant conferring resistance to aminocoumarin, evgS, catB10, MexZ, otr(B), MCR-6.1, mel, tetQ, Enterococcus faecium cls conferring resistance to daptomycin, AcrF, MexH, efrA, Mycobacterium tuberculosis thyA with mutation conferring resistance to para-aminosalicylic acid, dfrA8, Escherichia coli soxS with mutation conferring antibiotic resistance, MexL, macB, carA, mtrE, TolC, emrK, tet(Y), tet36, Bifidobacterium adolescentis rpoB mutants conferring resistance to rifampicin, abcA, AcrS, novA, vanHD, vanSO, GOB-1, LRA-12, mdtP, adeF, Chlamydia trachomatis intrinsic murA conferring resistance to fosfomycin, vanG, vatH, MexV, pgpB, tet(43), vanSB, vanRD, bcrA, emrB, mexP, arlR, TriA, mdtB, FosC2, evgA, smeS, norA, mdsB, otrC, vanSE, vanO, VatI, farB, AIM-1, Escherichia coli nfsA mutations conferring resistance to nitrofurantoin, gadW, vanRA, lsaA, msrC, AxyX, OprZ, marA, vanRI, cdeA, srmB, adeI, adeN, tet(35), SPG-1, vanTE |
| *Bifidobacterium kashiwanohense* | ugd, bopD, rtxB, kfiC, cpsJ, wbkA, wbbM, cpsG, cpsO, lap, irtB, hitC, essC, mbtJ, lytC, tcnA, gtrB, wzm, wzt, pspA, wcbQ, coxH2, pitA, bplF, cap8D, iap/cwhA, wzb, rffG, farB, fbpC, pebA, neuA/flmD, cpsE | ugd, vmlR, PmrF, vanJ, vanUG, tetA(60), poxtA, salA, msrA, efmA, efpA, vgaB |
| *Bifidobacterium adolescentis* | srtC, coxFIC1, srtD, hitC, feoB, bopD, kfoC, cpsG, lap, bprB, ami, clbM, bspR4, basI, clbJ, tagT, prn, clpC, iroC, lytC, ctrD, mucD, cgs, pilC, wzt, Cj1437c, cylB, cyaB, cpsD, xcpA/pilD, msbA, bfmS | nalC, tlrC, AAC(6')-Iv, efrA, baeR, hmrM, Staphylococcus aureus rpoB mutants conferring resistance to rifampicin, tetW, patB, bcrA, Streptomyces rishiriensis parY mutant conferring resistance to aminocoumarin, vgaB, otrC, Bifidobacterium ileS conferring resistance to mupirocin, lmrB, vanHF, Pseudomonas aeruginosa CpxR, efrB, cmx, tetA(60), tetA(46), Staphylococcus mupA conferring resistance to mupirocin, Staphylococcus mupB conferring resistance to mupirocin, cdeA, vanSD |
| *[Eubacterium] rectale* | algZ, algR, essC, phoR, phoP, ppkA, cylA, kpsF, fleN, cheD, chpD, bopD, cpsJ, pchR, wbbM, bsh, ctrD, allS, feoB, clbM, CBU_1594, lap, capA, xcpS, exsA, Cj1437c, lipF, srtB, fliB, cheY, bfmS, bfmR, wcsS, lsgC, cpsL, Cj1137c, wcsT, algC, rfbM, algI, prt, wbkA, pgaC, bplG, bplF, fsrA, hlyB, lpg2434, algU, virD4, AHA_1389, flmH, sipA, hitC, lirB, relA, chuW, mucD, fbpB, fbpC, pvdN, wzt, mf2, acfB, wcbQ, cylR2, bplA, coxFIC1, fimB, flpF, fliC, mucP, cpsB, cpsA, pdgA, fliA, cheW-2, cheA, cheB, flhF, flhA, flhB, fliR, yscS, fliP, fliO, fliN, fliM, motD, flgD, bscN, yscL, fliG, fliF, fliE, flgC, mgtB, tsr, ascN, pvdH, htpB, bauE, flhB2, acpXL, cdpA, Cj1436c, cgs, phzH, lfgK, fliW, fliS, cpsE, cps4B, cpsC, cpsD, bsc3, cps4H, wbbN, licD, bsc1, rffG, lgtF, cpsI, hddC, cps4D, srtG2, ylxH, bprC, srtG1, clbG, cylG, cylI, fabZ, msbA, chpA, pilG, hlyA, CBU_1566, flhG, lspA, fimE, kdtB, fbpA, cheR, mycP3, iap/cwhA, wlaN, EF3023, nanJ, nagK, pebA, Cj1419c, hspX, afaG-VII, mshG, pilT, xcpR, clpP, fepC, mntB, cps4E, cap8C, licC, licA, tlpB, cpsN, fleR/flrC, pilS, ybtP, wbcG, pseC, flaA, lfgL, flgK, lpg2370, pilH, mbtJ, lafS, fctA, Cj1136, kfiC, wzm, kfoC, ptmB, wzb, mtrD, cpsG, bsaN, flaB, fss3, bprB, flgG, ptxR, pkn5, shuU, plcD, ricA, cpsF, cpsH, pdxA, clbP, manC, neuD, cpsM, srtC, allB, rtxA, galF, wbcA, ybtA, pscL, flgB, cyaB, mshO, tapD, pilE, fliD, maf4, nueA, nueB, pseG, fleI/flag, coxH2, hasC, flgE, pomA2, fliQ, pppA, pvdL, gtrB, manB, cps4C, Cj1434c, entA, AHA_3493, lmb, acrB, luxS, yhxB/manB, orfM, lytC, iga, cheV, cdsN, hlyD, pilB, pilC, phzE1, yscN, galE, CBU_1434, wbkC, AHA_1829, clbB, scpA, clpE, clbL, lpg2936, cps4A, cylM, cylB, mf3, fss1, IlpA, entE, pilE3, fsrC, zmpC, allC, pilW, pspA, scpB, feoA, spa47, shuV, fhaB, clbD, cap8J, recN, rtxD, wcaH, irtA, mgtC, cbpA, toxA, lspG, vipD, ddhB, ddhA, gmhA, wcbN, wcbL, neuA, bepA, flgJ, fimT, bplB, lpsB/lpcC, algA, Cj1417c, wbpB, Cj1135, coxU2, lapB, mbtE, algB, eccA3, cps4F, tcpC, clpC, wbcJ, lpxE, Cj1432c, cap8D, clbF, pvdI, pgaB, neuC, neuB, pilR, gtcA, amoA, hysA, aslA, lgtC, ybtE, pvdS, fha1, gbs0628, xcpT, rfaD, wcbK, lgtA, wbaP/rfbP, pvdJ, virB4, fleQ/flrC, bapC, pmlR/bspR1, Cj1438c, enhC, hldE, bplC, ybtQ, entF, flaH, cpsO, ideR, glf, ebpA, coxH3, tagT, cpsK, clbN, cheB-2, icaA, inlF, spaD, adeF, eccA1, prsA2, wbpL, wcbJ, colA, inlA, cap8N, srtD, lsgE, fsrB, fliI, ecbA, rtxB, cysC, pseE/maf5, brkB, oatA, ybtS, srtC-1/srtB, gmhA2, rfpB, ugd, gluE, paa, hgpC, clbJ, iroC, tcnA, tviC, cps4I, rfaE, kdsA, tapT, pilD, basF, lpg2628, rrgB, gbs0631, isdE, ssaN, bspR2, csgD, icaR, ompA, gnd, tlpC, mntA, lsgF, fleS/flrB, cap8B, cba, kpsT, spaA, spaC, maf-2, flmD, pseB, pseD/maf2, vscL, cheW, mshM, inlJ, flgL, pilA, irp1, spvR, wbpZ | vanRB, vanSB, evgA, lsaA, tsnR, tet(D), Escherichia coli soxS with mutation conferring antibiotic resistance, Bifidobacterium ileS conferring resistance to mupirocin, PmrF, gadX, efrB, NmcR, ACT-10, mepA, tet(35), Bacillus subtilis pgsA with mutation conferring resistance to daptomycin, marA, evgS, vanRC, basS, vanSD, mecI, VatI, hmrM, Chlamydia trachomatis intrinsic murA conferring resistance to fosfomycin, vanHD, srmB, Escherichia coli marR mutant conferring antibiotic resistance, Pseudomonas aeruginosa soxR, abcA, lmrC, vanRA, vanO, BJP-1, salA, golS, bcrA, AAC(6')-Isa, vanRF, oleB, adeL, ramA, vgaE, mecB, MdtK, tetX, vanSO, bacA, Mycoplasma hominis parC conferring resistance to fluoroquinolone, Streptomyces rishiriensis parY mutant conferring resistance to aminocoumarin, vgaB, mel, Staphylococcus aureus fusA with mutation conferring resistance to fusidic acid, mecR1, vanZF, otr(A), LlmA 23S ribosomal RNA methyltransferase, smeR, Staphylococcus mupA conferring resistance to mupirocin, ErmS, vanRD, tet37, MexR, poxtA, lsaB, vanRM, oleC, vanTE, tetO, mdtA, macA, vmlR, vgaD, tetA(48), adeR, AAC(6')-Ix, AAC(3)-Ia, vanHM, vgaALC, tlrC, ACT-22, vanRN, optrA, rphA, AAC(6')-Ip, vanE, vatB, vanUG, msbA, lmrD, rosB, kdpE, cpxA, AcrF, Mycobacterium tuberculosis gidB mutation conferring resistance to streptomycin, dfrA10, lsaE, PmpM, vanYG1, otrC, Enterococcus faecalis cls with mutation conferring resistance to daptomycin, Pseudomonas aeruginosa catB6, efmA, Mycobacterium tuberculosis rpoB mutants conferring resistance to rifampicin, arlR, Staphylococcus mupB conferring resistance to mupirocin, ACC-2, TaeA, msrC, LRA-8, YojI, gadW, lsaC, tetT, vanHF, AAC(6')-Ie-APH(2'')-Ia, tetQ, vgaC, vanHO, mepR, vanSG, vanSF, vanSA, Escherichia coli EF-Tu mutants conferring resistance to Enacyloxin IIa, SAT-4, vanRI, smeE, clbC, blaR1, vanSM, tet(W/N/W), baeR, smeS, patB, AcrS, arnA, mecC, Mycobacterium tuberculosis kasA mutant conferring resistance to isoniazid, Escherichia coli fabI mutations conferring resistance to isoniazid and triclosan, cdeA, efrA, cmrA, vanRO, dfrA24, vgaA, tetS, tetB(P), Staphylococcus intermedius chloramphenicol acetyltransferase, vanZA, carA, tetA(46), apmA, vanSE, ANT(4')-IIa, vanWB, vanSC, Pseudomonas aeruginosa catB7, rphB, MexS, tetM, mecA, vatD, adeS, tetB(46), macB, MSI-1, bcrC, catB3, Enterococcus faecium cls conferring resistance to daptomycin, Laribacter hongkongensis ampC beta-lactamase, MuxB, ErmH, MCR-8, bmr, vanTN, catB10, MexZ, tcr3, Neisseria meningititis PBP2 conferring resistance to beta-lactam, Mycobacterium tuberculosis embR mutant conferring resistance to ethambutol, tetA(60), OXA-56, mecD, tet36, vanXYN, vanM, nalC, CAU-1, Pseudomonas aeruginosa CpxR, Bifidobacterium adolescentis rpoB mutants conferring resistance to rifampicin, ANT(4')-IIb, cfrA, vanRL, arlS, QnrVC3, vanSN, MuxA, baeS, adeN, ErmN, CMY-29, Streptococcus pneumoniae PBP2x conferring resistance to amoxicillin, Staphylococcus aureus rpoC conferring resistance to daptomycin, ugd, tetW, vanYB, vanRE, dfrA8, blaI, OXA-454, ErmD, Enterococcus faecium EF-Tu mutants conferring resistance to GE2270A, Staphylococcus aureus murA with mutation conferring resistance to fosfomycin, vanRG, msrA, sul1, clbB, sdiA, cmeR, tap, mtrR, bcrB, vanSL, OXA-665, vanWG, AAC(6')-IIb, EdeQ, Escherichia coli soxR with mutation conferring antibiotic resistance, Campylobacter coli chloramphenicol acetyltransferase, eatAv, vatA, vanXYL |
| *Coprococcus catus* | plcD, clbA, phzH, fliI, algU, srtC-1/srtB, pilB, fss1, pilC, pilT, bopD, msbA, phoR, cbpG, cylA, bfmR, bauE, clbM, exsA, fepC, isdF, isdE, fimB, AHA_1389, fbpC, virD4, flpF, flpH, srtC1, sipA, coxFIC1, hitC, eccA3, cylR2, flmH, relA, coxH2, clbL, hlyB, EF3023, algZ, pvdM, cheY, mgtB, manB, clpC, phoP, bfmS, CBU_1566, hlyA, barB, flhF, acpXL, ppkA, cylB, ideR, fliB, gtrB, algR, hspX, pchR, pspA, cbpA, licA, Cj1138, wzb, feoB, licC, cylG, pdgA, srtG1, mbtJ, iroC, srtG2, ascN, licD, fbpB, mntB, ecbA, allB, toxA, nagK, lafS, Cj1437c, feoA, phzE1, clbD, allS, lap, pvdS, tlpB, lpg2359, cylI, galE, aslA, cdpA, clbP, lpg1449, wbbM, allD, wzm, lytC, hasC, chpD, mgtC, ybtE, inlJ, wlaN, cps4H, gtcA, luxS, rtxB, wbpB, pilH, cbpD, iap/cwhA, cylS, cylM, kfiC, bplF, spaG, ybtQ, lirB, algI, cyaB, Cj1419c, orfM, Cj1432c, chuV, cpsA, rtxA, capA, phzD1, shuV, scpA, brkB, fleR, per, cps4J, clbB, iraA, bprC, vgrG-3, entE, cpsJ, fabZ, csgD, mshM, cps4A, rffG, cheB, lmb, icaR, flhG, ybtP, nagH, glf, wbaP/rfbP, tagT, clbF, clbG, ylxH, mf3, lpg2936, ebpA, cheA, irtB, pilR, pvdN, llsG, algW, ebpB, rrgA, inlF, cap8D, adsA, essC, irtA, fleR/flrC, hasB, bexA, spaD, eccA1, recN, clpE, clpP, hddA, fleQ/flrC, flgR, Cj1434c, yhxB/manB, fleS/flrB, srtC4, flpG, gnd, fbpA, pvdH, wbkC, cna, mucD, lepA, p97, lspE, pilE3, tapD, pilE, hldE, IlpA, fleQ, chuU, dep/capD, ptxR, afaG-VII, bplC, bsc1, wzt, fliA, cpsL, cpsH, scpB, phzF1, bscN, yscN, fimE, mycP3, AHA_3493, C2I, fleN, srtC, pilA, ebpC, cps4B, cpsC, cpsD, cpsG, wbbN, pppA, flgJ, kdtB, pvdL, pilG, bsc3, cdsN, rfaE, CBU_1434, wcbQ, mucP, cpsB, wbpZ, wbcA, prt, cpsE, pdxA, bplA, lidL, lspA, allR, pvcC, Cj1436c, bsh, entA, hddC, gmhA2, rfaD, htpB, cap8J, ctrD, ugd, lytB, wcsT, bplH, wbbO, cap8M, rpoN, clbN, chuW, nagJ, tapT, sfaX, lafK, flaB, fsrA, inlA, entB, cpsI, pilW, lgtA, waaF, rfaF, fbaB, mntA, bprB, ddhA, CBU_2076, lpeA, nanJ, ibeA, rrgB, pkn5, fha1, ssaN, psaA, vscN, gbs0628, cps4I, cpsM, wbcG, lsgC, bplG, pce, tsr, ybtS, pchA, pebA, EF0818, bplL, lpxE, kdsA, fsrC, tcpC, chpA | vgaB, vanSM, srmB, vanXYG, arlR, patA, vgaE, MdtK, Salmonella serovars soxS with mutation conferring antibiotic resistance, eatAv, msrA, cdeA, vanHD, efrB, TaeA, lsaA, vanSD, Escherichia coli soxS with mutation conferring antibiotic resistance, tap, lsaB, Pseudomonas aeruginosa CpxR, evgS, vmlR, vanSE, vanRM, lsaE, optrA, vanRO, salA, cpxA, tlrC, vanTN, lmrC, MuxA, NmcR, PmrF, vanZA, vanHM, efrA, evgA, vgaD, macB, golS, ramA, mepA, Bifidobacterium ileS conferring resistance to mupirocin, otrC, vanSC, otr(A), Bacillus clausii chloramphenicol acetyltransferase, adeL, vanRA, Escherichia coli parC conferring resistance to fluoroquinolone, sul1, tetX, carA, tsnR, patB, gadX, ACT-37, Erm(37), marA, hmrM, vanSB, nalD, tetW, tetA(46), smeS, vanSO, kdpE, vanSA, mtrR, gadW, msrC, vanRF, qacB, vanRB, vanB, vanSF, bcrA, msbA, vanUG, Escherichia coli nfsA mutations conferring resistance to nitrofurantoin, vgaA, Haemophilus influenzae PBP3 conferring resistance to beta-lactam antibiotics, sul4, poxtA, lsaC, mepR, bcrC, Erm(30), APH(2'')-IIa, tetB(46), mecD, tet(35), Enterococcus faecalis cls with mutation conferring resistance to daptomycin, adeS, tet37, LlmA 23S ribosomal RNA methyltransferase, tetB(60), Streptomyces rishiriensis parY mutant conferring resistance to aminocoumarin, Mycoplasma hominis parC conferring resistance to fluoroquinolone, lmrD, Escherichia coli EF-Tu mutants conferring resistance to Pulvomycin, norA, abeM, PmpM, LRA-17, vanXYL, MexR, mgrA, arlS, DHA-5, vanRE, msrE, AAC(6')-I30, smeR, MexZ, vanHF, rphA, oleB, abcA, tetA(60), Mycobacterium tuberculosis gidB mutation conferring resistance to streptomycin, ugd, vanRD, SPG-1, tetA(48), Enterococcus faecium cls conferring resistance to daptomycin, Chlamydia trachomatis intrinsic murA conferring resistance to fosfomycin, bacA, vanWB, vanHA, MexT, Enterococcus faecium EF-Tu mutants conferring resistance to GE2270A, mecB, arnA, vgaC, Staphylococcus aureus fusA with mutation conferring resistance to fusidic acid, vanC, oleC, Escherichia coli EF-Tu mutants conferring resistance to kirromycin, CRP, tet36, Clostridium perfringens mprF, tetQ, emrR, AcrS, AAC(6')-Isa, vgaALC, tetM, dfrA19, AIM-1, Staphylococcus mupB conferring resistance to mupirocin, cfrA, vanZF, Neisseria meningititis PBP2 conferring resistance to beta-lactam, MexS, Mycobacterium tuberculosis kasA mutant conferring resistance to isoniazid, YojI, mel, OXA-209, baeR, LRA-9, vanSN, vanYF, cmeR, adeN, vanRI, baeS, OXA-54, mdtG, mecR1, apmA, Bifidobacterium adolescentis rpoB mutants conferring resistance to rifampicin, vanHB, tet(D), tetS, THIN-B, CPS-1, tetR, EdeQ, Escherichia coli acrR with mutation conferring multidrug antibiotic resistance, LRA-19, mecA, adeR, mtrA, vanRN, tet(W/N/W), clbC, Staphylococcus mupA conferring resistance to mupirocin, clbA, basS, vanRG, AAC(3)-Ia, novA, vanYB, vanSG, pgpB, vanSL |
| *Phoenicibacter massiliensis* | fbpC, fss3, fctA, srtC1, exsA, iap/cwhA, allB, clpV/tssH, fss1, AHA_1389, bopD | vgaA, tet(D), tetR, efpA, vanUG |
| *Dialister sp. Marseille-P5638* | licD, per, gmhA, wcbN, hddC, lgtA, wzt, allS, fepC, chuU, chuT, fbpC, bfmR, farB, Cj1437c, relA, essC, clpC, bsc1, hitC, mucP, cpsA, ppkA, pppA, wbkC, mgtB, Cj1436c, pebA, fbpB, lytC, eptC, tapD, fepA, chuW, ideR, ugd, hasC, rffG, gtrB, ptxR, clpP, cpsG, rfaE, pilQ, lpxE, lgtC, cpsO, lspG, yapE, lap, icl, cdpA, entE, sodB, pvdD, cap8J, pvdH, IlpA, opsX/rfaC, lsgC, tviC, acrB, mtrC, allB, phzF1, kdtB, algC, eccA1, CBU_1594, fliA, htrB, kpsF, kdsA, kdsB, lpxK, waaA, msbA, lpxB, hlyA, chuA, pgaC, pce, ybtS, phzE1, lbtC, algZ, algR, msrA/B(pilB), icaR, adeF, mtrD, phtA, icaB, clbG, cylG, cylI, flmH, cylA, adeH, cpsJ, acrA, phoR, pvdN, hlyB, iroN, mgtC, CBU_1434, flpC, CBU_1566, flhF, phoP, ricA, lipF, lbtB, lpxD, mtrE, algW, cpsB, iap/cwhA, sipA, clbL, coxH2, manB, flpG, flpF, flpH, dep/capD, cdsN, spa47, cps4I, wecA, Cj1417c, vasH/clpV, wzb, cpsI, cyaB, iroC, lmb, mntB, chuV, lpxC, fabZ, lpxA, lidL, clbM, xcpR, pilT, mshG, bsc4, gmhB, exsA, feoB, feoA, lasA, AHA_1389, ompA, glf, cpsE, bsc3, waaF, cps4H, wbaP/rfbP, orfM, lspA, napA, recN, clbF, flhG, isdE, wcbT, fepB, fleN, pchD, fliB, bfmS, csgD, ascN, algB, bplB, wcbR, clbB, rfaF, gtcA, spvR, CBU_2076, wcbL, galE, wbbM, lpnE, bprB, afaG-VII, clbP, adeG, acpXL, fimE, lspF, lspE, fhaC, fhaB, exeG, cyaD, wzm, inlA, lirB, bauE, bspR2, boaB, shdA, badA/vomp/brp, ddhA, ddhB, prt, wbcA, fbpA, clpE, algA, rfpB, lsgE, cpsM, waaV, prn, pscN, bplC, clbH, mntA, kdtA, hspX, ureG, flpB, exeD, gspG, bimA, rtxA, shuU, bauA, shuT, chpA, fleS/flrB, tcfA, pilH, yhxB/manB, htpB | efrB, adeL, vmlR, lsaB, evgS, cmlA5, patB, LRA-8, tetA(46), vgaB, clbC, arnA, rphA, NmcR, mel, MCR-4, CRP, ugd, vgaD, oleC, adeN, pgpB, Haemophilus influenzae PBP3 conferring resistance to beta-lactam antibiotics, gadW, apmA, srmB, mefA, mdtB, TriA, vanTN, vanWG, oqxB, optrA, Streptomyces rishiriensis parY mutant conferring resistance to aminocoumarin, Bifidobacterium ileS conferring resistance to mupirocin, tsnR, lrfA, tet36, arlR, cmeR, mexX, mexQ, otrC, lsaE, PmrF, Mycobacterium tuberculosis kasA mutant conferring resistance to isoniazid, bacA, tet(35), Pseudomonas aeruginosa soxR, tetB(46), MexV, mtrE, golS, Klebsiella pneumoniae acrA, LpeB, vanHO, vanSD, salA, Escherichia coli parC conferring resistance to fluoroquinolone, PmpM, tet37, amrA, Pseudomonas aeruginosa emrE, vanSB, vanRO, vatF, tet(L), bmr, opcM, Chlamydia trachomatis intrinsic murA conferring resistance to fosfomycin, Erm(42), Pseudomonas aeruginosa CpxR, sul1, vanRL, Mycobacterium tuberculosis pncA mutations conferring resistance to pyrazinamide, vatB, tetM, lsaC, mepR, emrK, Type A NfxB, msrC, vgaE, msrE, catB9, blt, rosA, MdtK, Escherichia coli marR mutant conferring antibiotic resistance, tet(33), Staphylococcus mupB conferring resistance to mupirocin, msbA, vgaA, YojI, vanN, tet(D), dfrA7, mecC, abeM, macB, mtrC, Neisseria meningititis PBP2 conferring resistance to beta-lactam, vanHF, lmrD, Escherichia coli EF-Tu mutants conferring resistance to kirromycin, vanSO, vanUG, emrB, AcrE, Enterococcus faecium EF-Tu mutants conferring resistance to GE2270A, vgaALC, ICR-Mc, mexP, mecA, FosA2, IMI-1, lmrC, mtrD, ceoA, sdiA, sul4, tlrC, ramA, FOX-9, lsaA, abcA, mexN, dfrA10, vanRF, emrR, Haemophilus parainfluenzae parC conferring resistance to fluoroquinolones, mtrR, MexZ, mdtC, mepA, norA, SLB-1, pp-flo, MuxC, evgA, Bacillus clausii chloramphenicol acetyltransferase, vanTE, cmrA, OpmH, L1 beta-lactamase, Streptomyces lividans cmlR, efrA, carA, MexJ, vanSM, arlS, msrA, otr(A), poxtA, tetW, MexD, spd, baeR, Escherichia coli soxR with mutation conferring antibiotic resistance, vanHB, vanHM, amrB, basS, vanRE, mtrA, oleB, Bifidobacterium adolescentis rpoB mutants conferring resistance to rifampicin, MCR-1, tetA(60), ErmH, OpmB, cmx, mdtE, acrB, AcrS, Escherichia coli nfsA mutations conferring resistance to nitrofurantoin, tetQ, smeR, vanSG, MexH, ErmO, iri, vanRB, SFB-1 |
| *Bacteroides heparinolyticus* | kpsD, cap8M, neuD, cylG, flmH, ybtE, per, iap/cwhA, mucD, cloSI, algB, mip, clbP, chuW, cpsJ, cpsO, rffG, algU, phzG1, algZ, adeF, fbpC, aslA, fcl, phoR, mtrD, fepA, cap8E, ddhA, Cj1437c, manAoAg, yhxB/manB, hlyB, tviC, icaB, tviB, adeG, wbcC, prt, cpsG, wbbN, wbkA, lgtC, cpsI, prsA2, cps4L, bprB, pilR, wcsT, lsgC, neuB, hpt, AHA_1389 | Staphylococcus intermedius chloramphenicol acetyltransferase, catB8, evgA, CMY-11, Chlamydia trachomatis intrinsic murA conferring resistance to fosfomycin, PmrF, mdsA, OpmB, vgaE, vanSA, macB, mdtF, patB, abcA, DHA-18, lsaE, vanSE, lsaA, MexW, MexD, tet(35), cpxA, lmrD, evgS, baeS, apmA, PmpM |
| *Ruminococcus bicirculans* | cap8D, cheA, algZ, cdpA, clbI, cylG, cylI, cylZ, lpg2628, fbpC, clbL, hitC, phoP, phoR, AHA_3493, bfmR, bfmS, fliA, cheW, fliB, feoB, feoA, ideR, wecA, kdtB, pvdH, cylR2, allB, flpH, flpG, fleN, ascN, yscN, galE, cpsB, lap, pkn5, flmH, AHA_1389, srtC4, spaD, lytC, virD4, Cj1437c, algC, wcbQ, pdgA, chpD, hlyA, msbA, clbM, wcaH, mgtB, essC, pfbA, cyaB, clpC, hlyB, eccA1, algU, capA, cpsC, CBU_1566, yhxB/manB, chuW, relA, mucP, cpsA, nanI, orfM, flhF, pvdF, cps4C, cap8B, cpsE, wbkA, lsgC, wcsT, wcsS, cpsL, bplA, ast, sipA, bauE, cylA, wzm, algR, bsc1, cpsJ, kfiC, lsgF, ybtP, wzt, wbbM, cpsK, ybtS, phzE1, pvdN, iap/cwhA, allS, kfoC, fsrB, rcsB, cap8P, acpXL, fepC, Cj1436c, phzH, plcA, lasA, rtxB, hspX, pebA, ptmA, farB, entE, Cj1438c, wbcA, rffG, exsA, coxH2, CBU_1434, wbpL, wzt2, mntB, mntA, lmb, inlF, vscN, algI, clpP, lirB, ricA, clbB, fabZ, cgs, htpB, mucD, cheW-2, cheB, cheR-3, bepA, clbP, rfaE, tviC, ugd, lpnE, bopD, cps4A, hddC, shuV, pilT, fimB, fleQ/flrC, ureA, ureB, ureE, ureG, ureH, irtA, plcD, flhG, lpg2936, fsrA, fleS/flrB, wbkC, pppA, ppkA, ybtA, gtrB, cap8N, plc, fss1, lidL, icaR, gbs0628, srtD, bprB, cap8C, inlA, pilD, pspA, cpsN, cpsG, cpsM, srtB, adeF, cheD, wbcG, rfpB, neuD, cap8M, cap8J, kdsA, pilE, cheB-2, fbpA, gtcA, ptxR, pilC, srtG2, wcbM, cheY, afaG-VII, ybtQ, clpE, fimE, espX6, bexA, lspA, bsh, clbF, per, pilS, enhC, vpdB, clbA, pchG, pchF, irp2, ybtE, pchC, gspE, cna, fbpB, mycP3, recN, pce, ecbA, toxA, ureF, pgaB, vpdC, srtC, lpg2370, inlJ, sfaX, lpxE, coxFIC1, cdsN, pscN, licA, clbG, chpA, glf, CBU_0270, hmw3, tagT, fleR/flrC, p97, lapB, eccA3, flpF, cps4B, spa47, clbH, mip, lpg2434, amoA, clbJ, CBU_1594, algW, lgtC, shuU, wzb, vipD, chuV, srtE, wbpB, cpsD, lpeA, mf3, scpA, Cj1417c, pgaC, pilH, Cj1434c, iroE, rtxA, tapB, lspG, pilV, pilM, pvdL, chuU, luxS, ssaN, cadF, pchR, IlpA, srtC1, vipF, btpB, kpsT, fhaB, nagK, tviE, hldE, cps4K, virB4, iga1, fliI, hasB, mshG, pchH, bplF, algD, AHA_1829, virB4-1, fbaB, fsrC, pchD, wcbK, gmd, manC, Cj1416c, licC, iroC, mtrD, iutA, cpsO, fss3, bplC, bprC | arlR, baeS, Chlamydia trachomatis intrinsic murA conferring resistance to fosfomycin, oleI, Streptococcus pneumoniae PBP2x conferring resistance to amoxicillin, ErmT, YojI, Planobispora rosea EF-Tu mutants conferring resistance to inhibitor GE2270A, otrC, macB, vanZA, smeR, adeS, rphA, Escherichia coli soxS with mutation conferring antibiotic resistance, vgaB, poxtA, mecA, cdeA, MdtK, PmpM, vanSO, evgS, msbA, vanKI, vanTN, golS, tetQ, tet36, Bifidobacterium adolescentis rpoB mutants conferring resistance to rifampicin, Staphylococcus mupB conferring resistance to mupirocin, TaeA, vgaALC, vgaA, LlmA 23S ribosomal RNA methyltransferase, mecR1, Rm3, SAT-2, Bifidobacterium ileS conferring resistance to mupirocin, macA, apmA, tetA(46), blaR1, mecI, oleC, vanRO, PmrF, vatH, oleB, vanXYN, AAC(6')-30/AAC(6')-Ib' fusion protein, mel, vanXYE, NmcR, vanSD, msrA, carA, tetA(60), salA, patB, tsnR, tet44, vanTE, vmlR, tetS, RlmA(II), emeA, AAC(3)-Ia, Shigella flexneri parC conferring resistance to fluoroquinolones, Mycobacterium leprae gyrB conferring resistance to fluoroquinolone, vanB, tetB(P), vanXYC, lsaE, Neisseria meningititis PBP2 conferring resistance to beta-lactam, tetA(48), optrA, arlS, tlrC, Erm(30), tet(D), Escherichia coli EF-Tu mutants conferring resistance to kirromycin, abcA, bcrC, msrC, vatD, srmB, rosB, evgA, patA, MOX-5, ugd, arnA, lsaC, vgaE, Enterococcus faecium cls conferring resistance to daptomycin, vanSF, AAC(6')-Ib, marA, Bacillus subtilis pgsA with mutation conferring resistance to daptomycin, AcrS, nalC, cfrC, adeL, bacA, otr(A), vanRM, BJP-1, Escherichia coli gyrA conferring resistance to fluoroquinolones, Streptomyces rishiriensis parY mutant conferring resistance to aminocoumarin, hmrM, ramA, kamB, vanHO, novA, catB10, catB9, mtrA, adeN, vanXYG, Pseudomonas aeruginosa parE conferring resistance to fluoroquinolones, rphB, vanRF, QnrS8, Pseudomonas aeruginosa CpxR, mepA, vanHF, tetO, nalD, QnrB27, tetB(46), gadW, vatC, vatA, vanUG, lsaB, vanN, clbC, vgaD, lsaA, vanHM, tet(W/N/W), AAC(6')-Ic, Staphylococcus aureus fusA with mutation conferring resistance to fusidic acid, cpxA, tet(Y), EdeQ, ADC-20, Mycobacterium tuberculosis kasA mutant conferring resistance to isoniazid, vanSE, myrA, mepR, vanSN, SAT-4, vanSC, efrB, Escherichia coli UhpA with mutation conferring resistance to fosfomycin, vatE, tet37, adeR, efrA, tetB(60), mgrA, MexZ, vanSB, vanZF, vanE, Mycobacterium tuberculosis rpsL mutations conferring resistance to Streptomycin, Pseudomonas aeruginosa catB7, AAC(6')-Iad, vanD, vanRB, tlrB conferring tylosin resistance, mtrR |
| *Coprococcus sp.ART55/1* | clbI, cylG, cylI, cylZ, lpg2628, fbpC, clbL, hitC, phoP, phoR, AHA_3493, bfmR, bfmS, fliA, virD4, sipA, clbM, algR, algZ, algU, ybtP, wzm, wzt, wbbM, cpsE, chpD, cpsA, wbkA, cpsN, cpsG, cpsM, fimB, AHA_1389, fss1, srtB, relA, ugd, clbF, cdpA, allS, pilS, pvdN, iap/cwhA, clbA, pchG, pchF, irp2, ybtE, pchC, ideR, ybtQ, fepC, fbpB, coxH2, recN, rtxB, lasA, flmH, coxFIC1, bplA, exsA, feoB, cdsN, pscN, licA, pvdH, clbG, acpXL, chpA, cheA, algI, glf, cpsJ, galE, bopD, shuV, gtcA, CBU_0270, pdgA, Cj1436c, cgs, hmw3, gtrB, yhxB/manB, toxA, flhG, ybtS, phzE1, cyaB, tagT, pilD, fleR/flrC, lirB, p97, ppkA, lapB, lap, chuW, eccA3, flpF, cps4B, spa47, orfM, cheY, clbH, wcsS, mip, fleS/flrB, fliB, hspX, lpg2434, amoA, pebA, clbJ, mgtB, CBU_1594, lytC, bsc1, essC, bauE, algW, lgtC, mntA, shuU, msbA, wzt2, wzb, vipD, hddC, wcbQ, srtE, wbpB, lpg2370, cap8J, rffG, cpsD, cps4C, mntB, lpeA, pkn5, fsrA, inlA, mf3, cylR2, lspA, vpdC, feoA, scpA, kdtB, clpE, flhF, CBU_1434, mucP, cpsB, cps4A, pppA, wbkC, fbpA, allB, bsh, hlyB, clpC, Cj1417c, eccA1, kfoC, fimE, phzH, cna, pgaC, pilH, Cj1434c, bprB, iroE, wbpL, rtxA, hlyA, CBU_1566, lpg2936, tapB, pilT, pilC, lspG, pilV, pilM, ptxR, inlJ, cylA, pvdL, chuU, luxS, clpP, ssaN, Cj1437c, htpB, cadF, lpnE, algC, kfiC, pchR, IlpA, iga1, entE, cpsC, fliI, mshG, pilE, gspE, inlF, clbB, algD, irtA, cpsL, Cj1416c, licC, Cj1438c, mtrD, mucD, iutA, ecbA, rfaE, fleN, bexA | mecI, vanUG, mecB, mecR1, mepA, vgaE, evgA, evgS, lsaA, cpxA, lmrC, kdpE, cdeA, vanSL, baeR, msrA, vanVB, lsaB, vanZA, Escherichia coli soxS with mutation conferring antibiotic resistance, AAC(6')-Ir, dfrA19, ugd, lsaC, LlmA 23S ribosomal RNA methyltransferase, vgaA, oleC, vgaD, vanSF, carA, SAT-4, poxtA, srmB, tetT, Staphylococcus mupA conferring resistance to mupirocin, vgaB, lsaE, rphA, otr(A), vanHO, baeS, MdtK, vanSD, Mycobacterium tuberculosis kasA mutant conferring resistance to isoniazid, tetR, adeR, vanSO, TaeA, PmrF, gadW, ramA, adeL, NmcR, msrC, tlrC, tet37, patA, vanHM, golS, Erm(30), vanN, salA, Bifidobacterium ileS conferring resistance to mupirocin, Mycoplasma hominis parC conferring resistance to fluoroquinolone, Escherichia coli parE conferring resistance to fluoroquinolones, msbA, arlR, patB, mel, vanTE, PEDO-2, Chlamydia trachomatis intrinsic murA conferring resistance to fosfomycin, tetA(60), PmpM, vanSB, efrB, vanRO, vanSA, rphB, tetB(60), vmlR, optrA, tet(D), Bifidobacterium adolescentis rpoB mutants conferring resistance to rifampicin, gadX, apmA, tsnR, oleB, vanSN, MexL, msrE, AcrS, Neisseria meningititis PBP2 conferring resistance to beta-lactam, mecD, bacA, Streptomyces rishiriensis parY mutant conferring resistance to aminocoumarin, clbA, arnA, Mycobacterium tuberculosis pncA mutations conferring resistance to pyrazinamide, cmx, adeS, vanRB, Enterococcus faecalis cls with mutation conferring resistance to daptomycin, macA, sul1, Escherichia coli EF-Tu mutants conferring resistance to kirromycin, macB, vanZF, vanSM, tet(35), SMB-1, adeN, CARB-12, dfrA10, MexZ, OXA-2, mtrA, vanRC, marA, catB9, abeM, mecA, Streptococcus pneumoniae PBP2x conferring resistance to amoxicillin, vanTN, vanWG, MexT, vanRL, basS, blaI, abcA, tetX, tetB(46), hmrM, vanC, smeR, CPS-1, vanRA, AxyY, efrA, Streptococcus pneumoniae PBP1a conferring resistance to amoxicillin, vanRM, CMY-12 |
| *Desulfovibrio piger* | lap, nueA, cap8J, pebA, fbpC, clbM, cylG, htrB, msbA, allS, algD, fleQ/flrC, chpD, neuB, ddhA, ddhB, kfiC, pilR, fleS, Cj1437c, kdsB, algB, clpP, fleQ, prsA2, icmL/dotI, icmC/dotE, icmE, wbkC, fliA, bauE, shuV, hitC, fimE, eptC, chuW, allB, bplC, iap/cwhA, icmT, dotB, dotC, dotD, dotA, licB, phoR, mgtC, IlpA, icmO/dotL, icmP/dotM, bapC, icmB/dotO, icmK/dotH, cdpA, AHA_1389, ptxR, kdtB, clbB, wbbO, cap8M, kdsA, fepC, wbpZ, clpC, cheA, chuT, feoB, iroB, fleS/flrB, adeF, dep/capD, tviC, lpg2936, coxH2, ybtE, CBU_1434, phoP, coxFIC1, mip, hlyA, vscO, ascR, farB, pdgA, fimB, lidL, mucP, sipA, wcsT, mntA, lplA1, pchI, adeH, wbcG, ascN, plcD, wcsS, CBU_1566, katA, mtrD, bfmR, wcbT, mucD, CBU_1594, algI, flmH, bfmS, hspX, cpsB, bplF, mgtB, fleN, cylA, cadF, lspA, fleR/flrC, vecA, sopB/sigD, phzH, bplH, acrB, acrA, cylI, acpXL, pvdH, clbJ, rfaF, icmO, rck, tviB, lirB, mtrC, bopD, ipaJ, bicP, tagR, bcrD, lgtF, chuV, fabZ, aopN, vscT, flhG, bprB, msrA/B(pilB), galE, ppkA, fhaB, wbkA, prn, flgR, icmK, cheV, lafK, vscQ, bscN, vscL, vscJ, rfaE, pilS, icmB, tlpB, rfaD, exsA, phtA, vscU, ascT, bscS, ascY, lbtC, scc4, oatA, Cj1432c, hlyD, cyaB, slc1, orfM, lpxD, lpxA, vgrG-3, rffG, fliF, fliE, flgC, flgB, fliR, acrH, ascC, cylF, luxS, wbcA, hasC, gmhB, rpoN, wcbM, manC, htpB, capA, kpsD, fbpA, relA, gnd, recN, pgm, flhF, pvdI, pipB2, hlyB, clbG, pchD, cpsA, clbL, farA, fliM, rtxD, lsgC, algC, shuT, shuU, gmhA, tagS, tagT, fliG, bplD, mntB, lpeA, yapC, Cj1436c, pchA, phzE1, licC, cap8N, vscN, gtrB, tagQ, ascJ, tapF, lpnE, algA, feoA, wzm, wzt, ccmE, ccmF, yscQ, pchC, clbA, ssaN, cpsG, wecA, entE, virD4, virB4-t1, adsA, yscD, essC, adeG, fcl, gmd, ureG, manB, p97, bsc1, pchF, clbK, inlA, bfpH, mbtB, pilE, ugd, cpsI, cpsE, virB4, yscS, bopN, sfaX, entF, sycN, ccmC, wbaP/rfbP, pseB, irp1, escF, clpB/vasG, pilQ, pilN, pilL, virB11, cpsO, icmL.1, shdA, etpA, fleR, etpB, pipB | apmA, vgaD, otr(A), hmrM, optrA, NmcR, ugd, gadW, vanHD, evgA, basS, vanHB, rosB, vanRO, tet36, tet(59), msrC, tetB(60), YojI, lsaA, MCR-6.1, tetA(48), vanSB, tet(35), tlrC, adeL, MexS, salA, vanRF, vanRM, adeR, oleD, vanSA, MexV, sul1, vgaB, arnA, AAC(3)-Ia, oleB, Streptococcus pneumoniae PBP2x conferring resistance to amoxicillin, patB, Pseudomonas aeruginosa soxR, carA, Streptomyces rishiriensis parY mutant conferring resistance to aminocoumarin, Escherichia coli parC conferring resistance to fluoroquinolone, qacH, tet(Z), abcA, adeH, rpoB2, vgaE, adeF, vanB, cfrC, tetB(46), Enterococcus faecalis cls with mutation conferring resistance to daptomycin, poxtA, efrB, MexB, rosA, vanHM, tsnR, tetM, vanTN, Erm(43), LlmA 23S ribosomal RNA methyltransferase, Escherichia coli fabI mutations conferring resistance to isoniazid and triclosan, vanRD, emrY, mel, vanSC, lsaE, vanHF, vanRB, evgS, Mycobacterium tuberculosis kasA mutant conferring resistance to isoniazid, MexC, APH(7'')-Ia, Streptomyces lividans cmlR, PmrF, Staphylococcus aureus pgsA mutations conferring resistance to daptomycin, Escherichia coli emrE, CfxA4, vanRC, vanSO, mepR, vanRA, vanSL, Escherichia coli nfsA mutations conferring resistance to nitrofurantoin, marA, rphA, emrR, vanRG, pmrA, srmB, OprM, rphB, vanI, vatH, Staphylococcus aureus mprF with mutation conferring resistance to daptomycin, smeF, cpxA, abeM, lsaC, mdtP, QnrB15, msrA, msbA, Mycobacterium tuberculosis rpsL mutations conferring resistance to Streptomycin, Escherichia coli EF-Tu mutants conferring resistance to kirromycin, msrE, Escherichia coli acrA, bcr-1, vgaA, emeA, tet(31), blt, macB, tetS, vmlR, nalD, tetQ, ykkC, AxyY, mexY, TriB, adeS, oleC, Bifidobacterium ileS conferring resistance to mupirocin, tetT, Staphylococcus aureus rpoB mutants conferring resistance to rifampicin, amrA, Pseudomonas aeruginosa catB7, vanSM, vanSG, bacA, tet(J), mepA, mecC, Pseudomonas aeruginosa emrE, Staphylococcus aureus fusE with mutation conferring resistance to fusidic acid, Staphylococcus mupA conferring resistance to mupirocin, acrB, mdtA, TriA, MuxB, cphA4, sul3, Escherichia coli marR mutant conferring antibiotic resistance, MdtK, Escherichia coli UhpA with mutation conferring resistance to fosfomycin, fexA, tet32, patA, Staphylococcus mupB conferring resistance to mupirocin, vanD, Chlamydia trachomatis intrinsic murA conferring resistance to fosfomycin, smeR, Bifidobacterium adolescentis rpoB mutants conferring resistance to rifampicin, MexW, novA, vatE, QnrS5, CfxA3, lmrC, kdpE |
| *[Eubacterium] sulci* | isdF, pvdE, hitC, pebA, cps4D, clbF, htpB, wecA, fliA, fleQ, cpsO, AHA_1389, entE, escN, icaB, allB, bplC, phoR, wbkC, fbaB, allD, bplA, tviB, mf3, pce, ybtQ, algC, pvdL, fleQ/flrC, msbA, bepA, flgR, rtxB, clpE, tagT, mbtM, bfmR, flhF, fbpA, pvdM, kdsA, fleN, hlyB, yhxB/manB, algI, cna, bsc1, coxH2, flpF, CBU_1434, fimE, irtA, mgtB, ppkA, feoB, ctrD, pvdI, wcbQ, fbpC, rrgB, chuW, recN, chuV, fss1, lpg2936, bauE, barA, srtC-2/srtC, phzH, CBU_1566, clpC, lap, lpxD, hlyA, lytC, mucD, cpsA, ideR, cap8M, rpoN, chuU, spa47, acm, phoP, vscN, fleS/flrB, cylG, essC, cpsJ, neuD, lpg2359, clbD, plcD, nanA, cpa, bfmS, clbP, lspA, tcpJ, lafK, cpsG, ybtP, fabZ, per, lirB, Cj1436c, IlpA, cpsB, relA, clbB, psaA, mntA, algU, cylR2, barB, bexA, Cj1437c, cylA, toxA, fepC, lpeA, cyaB, ricA, cap8D, iroC, irtB, feoA, efaA, allS, eccA1, pavA, clbM, wzt, cpsC, pppA, pvdJ, pilR, phzF1, shuU, pvdN, bplE, orfM, fliI, acpXL, srtG2, clpP, flmH, lmb, lfiI, msrA/B(pilB), kdtB, cps4A, cylI, algZ, coxFIC1, ybtE, eccCb5, bplB, fepD, sipA, sdrE, fleR, kfoC | YojI, salA, patB, PmrF, tetX, tlrC, tet(35), vanSD, arnA, otr(A), vanTG, mepR, TaeA, vanHM, evgS, MdtK, msbA, optrA, tetA(48), iri, srmB, arlR, Bifidobacterium adolescentis rpoB mutants conferring resistance to rifampicin, vgaB, efrB, clbC, vanTN, tetO, tetT, hmrM, tsnR, Streptomyces rishiriensis parY mutant conferring resistance to aminocoumarin, vgaE, evgA, Bifidobacterium ileS conferring resistance to mupirocin, Neisseria meningititis PBP2 conferring resistance to beta-lactam, tetQ, lmrC, tetB(P), novA, baeR, LRA-19, poxtA, vanKI, lmrD, vanXYG, msrC, tet44, efrA, Chlamydia trachomatis intrinsic murA conferring resistance to fosfomycin, Staphylococcus aureus fusE with mutation conferring resistance to fusidic acid, otrC, Pseudomonas aeruginosa CpxR, PmpM, vanSO, rphB, oleB, oleC, vatH, vmlR, bcrA, Enterococcus faecalis cls with mutation conferring resistance to daptomycin, bacA, AcrS, CMY-62, tetB(46), tetB(60), tetA(60), Staphylococcus mupA conferring resistance to mupirocin, lsaC, tetS, tetA(46), abcA, tetM, vanSE, ErmO, macB, lsaE, LRA-8, Agrobacterium fabrum chloramphenicol acetyltransferase, vgaALC, patA, mecD, Staphylococcus mupB conferring resistance to mupirocin, vanXD, vanSM, Staphylococcus aureus fusA with mutation conferring resistance to fusidic acid, vanTE, NmcR, Escherichia coli EF-Tu mutants conferring resistance to kirromycin, tet32, mepA, Escherichia coli parC conferring resistance to fluoroquinolone, vanSN, MSI-1, vanI, mecC, vanRC, lsaA, mel, Escherichia coli marR mutant conferring antibiotic resistance, vanC, carA, vanSG, vanYG1, pgpB, SPG-1, cdeA, dfrA10, tet37, vatE, dfrA5 |
| *Eubacterium] siraeum* | srtB, bfmS, cyaB, cylA, gmhA2, bfmR, bprC, coxH2, phoP, phoR, bsh, wecA, kdtB, flhF, clpC, lgtC, ricA, pdgA, lytC, luxS, msbA, essC, fbpC, allB, kdsA, lap, fliA, algI, glf, cdpA, rtxB, cheD, cgs, Cj1416c, chuV, pebA, tapC, exsA, phzH, bauE, ibeA, orfM, CBU_1566, lirB, hgpC, tagT, Cj1437c, wbkA, wbbO, cps4H, cpsN, cpsM, cpsJ, kfiC, kfoC, clbM, iroC, fimE, bplA, ami, fes, allS, recN, cylR2, coxFIC1, cps4A, mgtB, ugd, feoB, capA, irtB, lipF, pgaC, fepC, xcpS, pilT, bopD, chuW, bscN, yscN, pvdH, csgD, wbkC, brkB, acpXL, lasA, hitC, AHA_3493, chpD, algZ, mucD, fss1, ctrD, feoA, fleR/flrC, fimB, fleS/flrB, virD4, AHA_1389, pspA, yhxB/manB, fleN, cpsG, pvdM, clpE, aslA, bsc1, cylB, ureG, ybtP, tviC, wcaH, algR, ecbA, fliB, eccA1, lpnE, licD, papI, relA, fliP, nagL, icaR, spaG, clbL, lsgF, srtC1, gtrB, shuV, wzt, galE, ureB, fliC, flhB, fliG, mucP, pppA, clpP, hlyA, tapB, tsr, cloSI, fliI, ybtQ, fliQ, fliR, htpB, pgaB, virB4/cagE, cpsC, lafS, bsaN, lfgG, pchI, ylxH, lpg2936, flgE, mntB, gtcA, fliS, fbpA, wcaI, virB4, hasC, fliM, flhG, lspA, cap8M, flhA, tssH-5/clpV, pilH, mshE, hlyB, kpsT, mbtA, tapD, iap/cwhA, llsG, cylI, pilE, Cj1432c, pomA2, ideR, pchD, cheA, lspG, rtxE, nanA, phzD1, flmH, btpB, Cj1419c, wbcA, clbP, lsgC, pvdO, cap8J, spa47, esaA, pvdN, bepA, magA/wzy_K1, pfbA, rrgC, lpxA, clbI, cheV, ddhA, wzt2, cdsN, pvdS, pscN, allC, manB, srtG2, ascU, mf3, enhC, srtG1, aut, cylG, xcpR, flgC, nagK, chuT, flgL, hldE, kdsB, bsc3, fabZ, waaG, fliF, pce, ppkA, flgD, motD, wbpL, Cj1427c, CBU_1434, cpsA, ddhB, caf1R, fliD, tagAB-5, pavA, lpg2370, cheR, wzb, IlpA, cpsB, papX, flgG, pilG, pilC, inlF, bexA, flgB, cdsV, entF, clbB, cheB | arlS, lsaE, msbA, vanXYE, vanRG, ramA, CRP, AAC(3)-Ia, evgS, vanSD, vanKI, Chlamydia trachomatis intrinsic murA conferring resistance to fosfomycin, vatB, otrC, vgaB, otr(A), optrA, MexS, Bifidobacterium ileS conferring resistance to mupirocin, tet36, tet44, bcrA, tlrC, carA, tetA(48), tetA(46), oleB, lsaC, Erm(37), macB, lsaB, patB, PmrF, MdtK, lmrD, nalC, mecC, aadA14, efrB, marA, mepR, adeL, ugd, TaeA, tetB(46), LlmA 23S ribosomal RNA methyltransferase, Bacillus subtilis pgsA with mutation conferring resistance to daptomycin, lsaA, mecI, blaR1, Escherichia coli EF-Tu mutants conferring resistance to kirromycin, CAU-1, oleC, THIN-B, Erm(42), vanWB, vanUG, bacA, bcrB, arnA, Streptomyces rishiriensis parY mutant conferring resistance to aminocoumarin, tsnR, RlmA(II), Escherichia coli soxS with mutation conferring antibiotic resistance, poxtA, evgA, tetS, efrA, vgaALC, vanRB, plasmid-encoded cat (pp-cat), tlrB conferring tylosin resistance, mphL, vanSM, Pseudomonas aeruginosa parE conferring resistance to fluoroquinolones, Shigella flexneri parC conferring resistance to fluoroquinolones, YojI, srmB, LRA-9, adeN, salA, sul1, vanHO, Escherichia coli EF-Tu mutants conferring resistance to Enacyloxin IIa, vgaD, vanTmL, D-Ala-D-Ala ligase, vanZF, novA, AcrS, vanI, vgaE, hmrM, msrC, cdeA, vanTN, Bifidobacterium adolescentis rpoB mutants conferring resistance to rifampicin, lmrC, bcrC, mepA, patA, Pseudomonas aeruginosa CpxR, adeS, vmlR, baeR, vanXYC, SAT-2, vanSG, mtrA, LRA-8, adeR, abcA, tetA(60), tetB(60), vanRC, mel, msrA, tetO, Mycobacterium tuberculosis kasA mutant conferring resistance to isoniazid, macA, kdpE, tet(35), cipA, clbC, gadX, tetW, vanSB, ErmB, PDC-74, NmcR, apmA, mecB, mecA, vanRF, Pseudomonas aeruginosa catB6, Rm3, mecD, vatD, Escherichia coli gyrA conferring resistance to fluoroquinolones, AAC(6')-Ie-APH(2'')-Ia, nalD, cfrC, clbB, vanSC, vgaA, mgrA, vanRI, vanRM, vanSE, tet32, vanN, ACT-33, Erm(36), MexL, Staphylococcus aureus pgsA mutations conferring resistance to daptomycin, QnrB48, vanG, vanSN, emrR, golS, smeR, Staphylococcus mupB conferring resistance to mupirocin, tet37, msrE, vanHA, cpxA, Staphylococcus aureus gyrB conferring resistance to aminocoumarin |
| *Bifidobacterium pseudocatenulatum* | cdsD, Cj1437c, lspA, lgtA, clbL, cdpA, bopD, capA, hlyB, gspG, bprB, fbpC, AHA_1389, essC, cps4A, ppkA, oatA, lplA1, phzE1, srtA, wecA, relA, Cj1436c, orfM, clpC, sipA, algR, tapO, bfmR, clpE, cylA, farB, lap, clbB, wcbQ, clpP, iroC, psaA, wzb, iap/cwhA, wbkC, chuW, cylG, hlyA, coxH2, fimE, wbbN, bplH, eccA1, phzH, rfaE, IlpA, luxS, phoR, pebA, hitC, bfmS, mbtE, pvdN, ugd, wbbM, wzt, kfiC, lytC, msbA, Cj1434c, flhF, flpF, mgtB, allR, gnd, exsA, bsc1, entE, cpsB, amoA, shuV, cap8J, srtC-1/srtB, srtD, pilA, wcbM, lbtC, Cj1417c, wbbO, phoP, mntB, fepC, glf, ylxH, pvdH, recN, fhaB, chpA, manB, fimB, cpsA, flmH, clbD, fliA, clbM, rtxB, lsgC, cps4H, tagT, lpg2936, rffG, CBU_1566, cyaB, pilM, tapD, pilC, xcpR, pilT, coxFIC1, lirB, irtA, allB, cpsJ, rfaD, cpsE, srtC4, pppA, mucD, bexA, barA, cylR2, cap8B, ybtP, ebpB, srtC, rcsB, cpsG, cpsO, Cj1137c, cpsL, pvdL, fbpB, wzm, spaG, htpB, feoB, ideR, ssaN, tviE, gbs0630, gbs0628, mip, licD, galU, wbcA, EF3023, fss1, mbtJ, cna, ebpA, mycP1, esxA, mycP5, eccC3, algU, eccCa1, galE, gbs0631, bauE, pgm, Cj1416c, bsh, rrgB | patB, vanN, msrA, vgaA, Staphylococcus mupA conferring resistance to mupirocin, hmrM, vanRC, Erm(41), poxtA, tsnR, Streptomyces rishiriensis parY mutant conferring resistance to aminocoumarin, Shigella flexneri parC conferring resistance to fluoroquinolones, Streptococcus pneumoniae PBP1a conferring resistance to amoxicillin, mecC, evgA, sdiA, golS, vanKI, RbpA, oleB, evgS, abcA, tet(B), vanRO, otrC, mtrR, efrA, QepA1, MexS, vgaB, dfrA10, arnA, lsaA, tetX, lsaB, Escherichia coli EF-Tu mutants conferring resistance to kirromycin, macB, vmlR, vanSC, tcr3, lsaE, vanSB, vgaD, mef(B), Bifidobacterium ileS conferring resistance to mupirocin, tet(45), ugd, PmrF, carA, marA, mel, salA, tetQ, cfrA, bacA, novA, lsaC, apmA, norA, tetA(60), optrA, vanHM, mphC, arlR, Bifidobacterium adolescentis rpoB mutants conferring resistance to rifampicin, emrY, fexA, AcrS, Mycoplasma hominis parC conferring resistance to fluoroquinolone, PmpM, catB10, smeR, mdtM, srmB, vanSO, gadW, Borrelia burgdorferi murA with mutation conferring resistance to fosfomycin, baeR, mecA, vanHO, efmA, cmlA6, Staphylococcus aureus fusA with mutation conferring resistance to fusidic acid, vanTE, lmrB, tet(K), tetA(46), Chlamydia trachomatis intrinsic murA conferring resistance to fosfomycin, bcrA, Staphylococcus mupB conferring resistance to mupirocin, CRP, Clostridium perfringens mprF, lmrD, vanHD, vanUG, sul1, AAC(6')-Iak, tet32, GOB-1, vatC, tet(35), Escherichia coli acrR with mutation conferring multidrug antibiotic resistance, adeR, Escherichia coli murA with mutation conferring resistance to fosfomycin, Haemophilus influenzae PBP3 conferring resistance to beta-lactam antibiotics, tetA(48), vanHA, farB, Bartonella bacilliformis gyrB conferring resistance to aminocoumarin |
| *Ruminococcus champanellensis* | bfmR, feoA, tagT, relA, fliB, tlpB, ideR, algW, fss1, virD4, fimE, lap, phoP, rffG, phoR, coxH2, AHA_1389, spaD, srtB, srtC-1/srtB, clpE, clpP, pvdM, sipA, ppkA, pppA, wbkC, fliI, clbP, pdgA, bplF, pvdN, cylI, fabZ, acpXL, wzm, wzt, algR, caf1R, srtD, srtG2, cps4H, hlyB, hlyD, clbM, msbA, ylxH, bsc1, lspG, gspE, tapT, chuW, flhF, bfmS, hitC, mucD, cap8M, manC, gmd, wbkA, wbpZ, cgs, llsG, algI, cdpA, mgtB, galE, chpD, bauE, clbA, galU, wbcA, prt, glf, cpsG, spaG, feoB, fsrA, cpsE, vscN, lasA, fbpC, hddC, mucP, yhxB/manB, Cj1437c, phzE1, ybtS, rtxB, inlA, cps4B, cpsD, cps4C, cylA, fsrB, htpB, clpC, lidL, CBU_1566, flmH, entE, cap8J, clbN, pchD, fliA, hlyA, cylR2, mf3, bplA, bopD, mgtC, fleR/flrC, algZ, fbpB, aslA, pvdL, Cj1416c, cyaB, pebA, irtA, wcbQ, scpA, bsh, bexA, coxU1, cpsA, cpsB, iap/cwhA, srtG1, ricA, algU, yscN, kdsA, recN, pvdH, esxA, essC, cps4K, hasC, irtB, cylG, phzH, toxA, EF3023, cheY, kpsT, lgtF, pspA, gtcA, exsA, sdrD, cylB, chuV, wcbK, sspA, allS, lytC, CBU_1434, fleN, fepC, lirB, lspA, eccA3, cpsJ, cpsC, Cj1137c, licD, coxFIC1, algC, lafS, allB, icaB, per, capA, fsrC, exeE, pvdD, pchE, tapC, pilD, orfM, colA, gtrB, pilE3, pgaB, fimB, tagAB-5, virB4, bplL, shuV, cap8D, pkn5, clpB/vasG | Bifidobacterium adolescentis rpoB mutants conferring resistance to rifampicin, baeR, optrA, Streptomyces rishiriensis parY mutant conferring resistance to aminocoumarin, evgS, vanUG, vanSD, clbC, arnA, CMY-10, Mycobacterium tuberculosis kasA mutant conferring resistance to isoniazid, vanO, lsaB, vanHO, vanRD, evgA, vanSM, gadW, vanSO, vanTN, tet37, poxtA, cdeA, Chlamydia trachomatis intrinsic murA conferring resistance to fosfomycin, carA, mepR, tsnR, vgaE, vanXYE, msrC, cpxA, Enterococcus faecalis cls with mutation conferring resistance to daptomycin, gadX, macB, arlR, Staphylococcus aureus fusA with mutation conferring resistance to fusidic acid, vanRO, vanSB, tetA(60), vatB, patB, tetB(46), Shigella flexneri parC conferring resistance to fluoroquinolones, bcrA, vanXYL, lsaA, abcA, MexL, arlS, vmlR, salA, ramA, TaeA, apmA, vgaB, lsaE, adeR, Staphylococcus mupB conferring resistance to mupirocin, tet(35), vanSF, lsaC, tetB(P), Bifidobacterium ileS conferring resistance to mupirocin, cfrA, PmpM, vanTrL, vanWI, SPG-1, mel, srmB, vanHA, smeR, tetA(46), otrC, oleB, adeL, Escherichia coli acrR with mutation conferring multidrug antibiotic resistance, tet(W/N/W), mepA, tetS, vanRF, tet32, tetT, vanSL, tet44, vatH, tet36, tetM, tetQ, vgaALC, AcrS, AAC(6')-Iu, vanXYG, Mycobacterium tuberculosis gidB mutation conferring resistance to streptomycin, vanXYN, lmrC, NmcR, ACT-1, golS, Escherichia coli EF-Tu mutants conferring resistance to Enacyloxin IIa, AAC(6')-Ie-APH(2'')-Ia, marA, pgpB, Escherichia coli fabI mutations conferring resistance to isoniazid and triclosan, msrE, vgaA, tetA(48), tet(D), MdtK, PmrF, vanZF, mefA, ErmG, Pseudomonas aeruginosa parE conferring resistance to fluoroquinolones, vanSE, YojI, tlrC, adeS, Escherichia coli EF-Tu mutants conferring resistance to kirromycin, QnrS5, msrA, tetO, rpoB2, ErmT, VatI, MexR |
| *[Ruminococcus] torques* | msbA, phoR, cpsJ, pspA, cylG, luxS, wbkC, nagJ, lytC, lpg2936, recN, lpg2359, mgtB, srtD, virD4, flgJ, AHA_1389, hitC, kpsT, bopD, kdsA, pvdN, fliA, eccA3, htpB, bfmR, fleS/flrB, fbpC, pavA, spa, fbpB, allS, hasC, cpsC, srtG1, cpsA, cps4B, cpsD, ybtQ, hlyB, fsrC, algR, wcsT, gtrB, vpdC, cheA, cdsN, bscN, acfB, bauE, pscN, clbM, EF3023, nagK, manB, lirB, lap, allB, exsA, wcaH, sipA, pvdH, algU, bsc1, coxH2, ddhA, algZ, phoP, srtB, fimB, clpP, rpoN, clbF, wbpL, fss1, brkB, fepC, iraB, shuU, Cj1436c, kdtB, ylxH, chpD, phzH, CBU_1566, hlyA, cylA, flhF, acpXL, fsrB, CBU_1434, gbs0628, lspA, fleN, Cj1419c, ppkA, cdpA, bfmS, hddC, Cj1417c, ricA, galE, mucD, wzx, yhxB/manB, cpsE, cyaB, prt, wbcA, gtcA, lytB, cap8D, wzm, wzt, bplF, bplG, icaA, glf, algI, IlpA, bplA, cps4D, cap8C, icaR, cylF, lplA1, lipF, rfpB, orfM, wcbQ, essC, flmH, clpC, ideR, plcD, flhG, pce, allR, ibeA, cba, mycP3, pebA, mf3, mucP, cpsB, phzE1, toxA, clbB, cap8J, pvdM, chuW, pilS, fabZ, wzb, fleR, srtG2, Cj1437c, rrgA, clbP, manC, wbpB, relA, papX, flpF, iap/cwhA, AHA_3493, inlF, fctA, fctB, pdgA, clpE, yscN, CBU_2076, wbkA, licD, cheY, fimE, cpsG, cpsL, wbcG, cps4A, cpsF, cps4I, cpsI, wbaP/rfbP, cps4F, icaB, rfaE, coxFIC1, pilH, algW, clbL, cpsO, pgaC, bplH, bplC, bplB, bplD, bplE, allD, rffG, srtC1, llsG, sfaX, ybtP, pitB, lsgC, wbbO, cylR2, cylI, adsA, licC, wcaJ, flgS, fsrA, ast, lpxE, pilB, srtC, ybtS, vscN, fleR/flrC, mgtC, ptxR, hspX, feoB, clbA, cps4H, lpg2628, nagI, pilR, pppA, ybtE, mbtB, clbJ, lapB, feoA, fliB, spaA, srtC-1/srtB, gmhA, vasH/clpV, pvdD, pvdL, wcbJ, cps4C, lsgF, lpsB/lpcC, lafS, nanJ, psaA, mntA, mntB, licA, Cj1434c, clbH, ureG, bsh, gnd, nagH, algB, pdxA, algC, wcbR, lmb, clbD, pchD, ctrD, chpA, lasA, phzD1, ugd, fliI, lgtC, Cj1416c, rtxE, irtB, cheB, spaD, cpsM, bepA, chuY, ascN, capA, CBU_1594, ebpC, srtC-2/srtC, allC, bauB, bauD, pfbA, cpa, scpA, eccA1, ssaN, lgtF, cps4E, tapT, tviB, legS2, sodCI, cap8M, bplL, pchR, pilM, chuV, Cj1137c, lafK, hldE, tsr, rtxB, per, neuD, bsc3, cps4K, cps4L, wcbL, wcbN, ddhB, barB, ebpA, rrgB, cheD, lafB, ideS/mac, rrgC, lidL, rtxA, neuC, flpG, ecbA, cheR, cbpD, iroC, entE, flgR, bprB, plcA, kfiC, wcbT, cheB-2, pspC/cbpA, cna, afaF-VII, lpg2370, essB, esaA, fbpA, mbtJ, pilT, pilC, cps4G, cap8L, isdF, isdE, rfbM, phnB, lpeA, ccmA, chuU, fbp54, wbpZ, pilX, inlA, mf2, wcbM, pilW, exeA, hmw1, tcpN/toxT, cgs, Cj1438c, xcpT, pilE, sodC, tviC, kfoC, wcbK, lsgA, ebpB, p30/p32, mshM, fhaB, esp, capB, gmhB, waaF, coxU1, pmm, cylB, fliC, fliF, bsaN, fss3, cps4J, pvdJ, pchC, inlB, pvdI, irtA, spvR, iga, scpB, shuV, kpsF, zmpC, ybtA, cbpA, srtE, srtC4, nanI, wlaN, flhB, wcbH, inlJ, tlpA, pkn5, hgpC, fha1, vasH, bapC, tagT, cheW, entF, lfgK, barA, futC, yaxA, bprC, rcsB, caf1R, ybtU, pchF, irp2, clbG, bexA, afaG-VII, fss2, gluP, cap8E, gbs0632, hasB, waaV, wbbN, pitA, prsA2, Cj1138, spaG | vgaA, vanSL, YojI, clbB, arnA, dfrA1, poxtA, golS, srmB, tet(35), vanHF, vanRO, arlS, vgaALC, FosA2, evgS, Haemophilus influenzae PBP3 conferring resistance to beta-lactam antibiotics, adeL, msrE, NmcR, salA, tetQ, Pseudomonas aeruginosa soxR, lsaC, vatB, PmrF, tsnR, evgA, vanSD, Chlamydia trachomatis intrinsic murA conferring resistance to fosfomycin, Pseudomonas aeruginosa CpxR, vanWG, mecB, mecC, bcrA, PmpM, hmrM, nalC, msrA, bacA, vmlR, vanSO, oleB, Mycobacterium tuberculosis gidB mutation conferring resistance to streptomycin, Streptomyces rishiriensis parY mutant conferring resistance to aminocoumarin, tlrC, msrC, AAC(6')-34, nalD, tetM, mepA, cdeA, MexT, ErmS, vgaE, tetA(46), patB, mel, TaeA, tet(W/N/W), Bifidobacterium ileS conferring resistance to mupirocin, Neisseria meningititis PBP2 conferring resistance to beta-lactam, apmA, Escherichia coli soxS with mutation conferring antibiotic resistance, vanHA, Mycoplasma hominis parC conferring resistance to fluoroquinolone, vanHB, gadX, AIM-1, vanTN, optrA, oleC, Escherichia coli EF-Tu mutants conferring resistance to kirromycin, adeS, MexZ, LlmA 23S ribosomal RNA methyltransferase, vatH, mepR, tetT, lmrD, msbA, vgaB, basS, Escherichia coli UhpA with mutation conferring resistance to fosfomycin, AcrS, FosK, sul1, ramA, tet37, lmrC, Escherichia coli acrR with mutation conferring multidrug antibiotic resistance, eatAv, Enterococcus faecium cls conferring resistance to daptomycin, vanSB, norA, vanHM, SPG-1, vanO, Bifidobacterium adolescentis rpoB mutants conferring resistance to rifampicin, vanZA, catB9, vanSC, AAC(6')-Ib8, mtrA, pgpB, LRA-13, lsaE, gadW, rosB, fusC, lsaB, macB, abeM, cpxA, bcrC, vanZF, vanXYE, MdtK, baeR, emrR, otrC, vgaD, mecI, vanWB, mtrR, ANT(4')-Ia, vatA, AAC(6')-Iu, kdpE, MexR, Enterococcus faecalis cls with mutation conferring resistance to daptomycin, vanRA, Mycobacterium tuberculosis kasA mutant conferring resistance to isoniazid, lsaA, tetA(60), vanRB, Erm(38), vanUG, tetA(48), vanRD, tet36, Staphylococcus aureus murA with mutation conferring resistance to fosfomycin, Mycobacterium tuberculosis folC with mutation conferring resistance to para-aminosalicylic acid, sul2, vatC, abcA, carA, tet(D), vanHO, Pseudomonas aeruginosa catB7, vanTC, mgrA, dfrA10, mecA, smeS, efrB, Salmonella serovars soxS with mutation conferring antibiotic resistance, arlR, otr(A), marA, KHM-1, mphC, SAT-4, vanRE, MSI-1, vanSA, smeR, Staphylococcus mupB conferring resistance to mupirocin, CRP, LRA-2, MexS, efrA, vanTG, vanRF, otr(B), Mycobacterium tuberculosis pncA mutations conferring resistance to pyrazinamide, mecD, tetB(P), novA, AAC(6')-Isa, ugd, Rm3, Escherichia coli EF-Tu mutants conferring resistance to Enacyloxin IIa, GOB-1, LRA-8, vanTmL, EXO-1, Staphylococcus aureus pgsA mutations conferring resistance to daptomycin, AAC(6')-Ie-APH(2'')-Ia, vanRM, Streptococcus pneumoniae PBP2x conferring resistance to amoxicillin, baeS, tetB(46), vanSF, SAT-2, vanHD, MexH, vanSG, vanF, tetO, tetX, ANT(4')-IIa, vanSM, tet(42), patA, adeR, clbC, vanRI, Mycobacterium tuberculosis inhA mutations conferring resistance to isoniazid, vanI, ErmH, Escherichia coli fabI mutations conferring resistance to isoniazid and triclosan, tet44, cmeR, dfrA8, vanXYG, vanL, TUS-1, catII, aad(6), OXA-397, vanE, rphA, Enterococcus faecium EF-Tu mutants conferring resistance to GE2270A, tetB(60), CAU-1, vanSE, vanTE, spd, Staphylococcus mupA conferring resistance to mupirocin, vanRL, rpoB2, MexL, sul4, vatE, blaR1, OXA-240, OXA-74, Escherichia coli nfsA mutations conferring resistance to nitrofurantoin, Staphylococcus aureus fusA with mutation conferring resistance to fusidic acid, rphB, adeN, vatF, cfrC, catS, catB8, SMB-1, vanRG, Bacillus subtilis pgsA with mutation conferring resistance to daptomycin, tet32, tetS, vanRN, vanRC, Sed-1, blaI, Escherichia coli marR mutant conferring antibiotic resistance, dfrA17, PEDO-2, Pseudomonas aeruginosa emrE, cfrA, iri, OXA-16, OXA-11, ErmO, Escherichia coli parC conferring resistance to fluoroquinolone, AAC(3)-Ic, vanVB, AAC(3)-IIa, VatI, TEM-144, npmA, Mycobacterium tuberculosis ndh with mutation conferring resistance to isoniazid, catB3, Enterococcus faecalis YybT with mutation conferring daptomycin resistance, vanSN, emrB, catB2, mecR1, LRA-9, OXA-162 |
| *Bifidobacterium breve* | phoP, tagT, phoR, bopD, msbA, mgtC, coxFIC1, inlF, wbaP/rfbP, clbM, cylA, allS, icaR, clpB, clbA, cap8J, fimB, bca, waaV, pgaC, algR, hitC, wecA, lirB, fbpC, hlyB, sodB, luxS, farB, pebA, iap/cwhA, rffG, wbkC, htpB, esxA, clpC, ylxH, pvdH, recN, lap, cna, coxH2, csgD, ybtP, bepA, btpA, ppkA, yhxB/manB, pvdM, exsA, fepC, prgB/asc10, EF0149, bfmR | evgS, lsaA, Rm3, basS, Pseudomonas aeruginosa CpxR, dfrF, patB, vgaE, oleB, mepR, adeN, srmB, mepA, lsaB, NmcR, tet(V), MexL, vanUG, catB9, lsaE, YojI, sul1, arlR, tetA(48), vgaB, vmlR, vanTC, carA, vanD, vanC, vanN, tetM, tetQ, tetB(P), fexA, salA, vanSB, otrC, Bifidobacterium ileS conferring resistance to mupirocin, poxtA, arlS, vanRI, marA, vanRM, apmA, golS, baeR, efmA |

**Table S11. Area under the ROC curves of metabolic biomarkers**

| **Metabolite** | **AUC** |
| --- | --- |
| alpha-ketoglutarate | 0.8 |
| Aminopterin | 0.7663 |
| N1-Methyl-2-pyridone-5-carboxamide | 0.7368 |
| 2-Isopropylmalic acid | 0.7326 |
| 3b-Hydroxy-5-cholenoic acid | 0.7305 |
| N-Acetyl-D-lactosamine | 0.7284 |
| N-Acetyl-L-Histidine | 0.7242 |
| D-Glucono-1,5-lactone | 0.7242 |
| Phenol | 0.7242 |
| 6k-PGF1alpha-d4 | 0.72 |
| Betaine | 0.7179 |
| L-Glutamate | 0.7095 |
| （S）-2-aminobutyric acid | 0.7095 |
| N6,N6,N6-Trimethyl-L-lysine | 0.7095 |
| Arg-Glu | 0.7095 |
| L-Pipecolic acid | 0.6989 |
| Promethazine | 0.6989 |
| Cytidine | 0.6968 |
| Uridine | 0.6863 |
| 11beta-Hydroxyprogesterone | 0.6568 |
| Perseitol | 0.6568 |
| 3-Hydorxy-3-methylglutaric acid | 0.6547 |
| Ramipril | 0.6547 |
| Taurocholate | 0.6547 |
| 9,10-DiHOME | 0.6526 |
| Ferulic acid | 0.6442 |
| 3-Aminosalicylic acid | 0.6337 |
| L-Glutamine | 0.6105 |
| Pyridoxine | 0.6063 |
| Dioscin | 0.5937 |
| Gly-Arg | 0.5537 |
| 1,3,5（10）-Estratrien-3,17 beta-diol 17-glucosiduronate | 0.5389 |
